# Long‐term risk of dementia following hospitalization due to physical diseases: A multicohort study

**DOI:** 10.1002/alz.12167

**Published:** 2020-09-04

**Authors:** Pyry N Sipilä, Joni V Lindbohm, Archana Singh‐Manoux, Martin J. Shipley, Tuomo Kiiskinen, Aki S Havulinna, Jussi Vahtera, Solja T Nyberg, Jaana Pentti, Mika Kivimäki

**Affiliations:** ^1^ Clinicum Department of Public Health University of Helsinki Helsinki Finland; ^2^ Helsinki Institute of Life Science University of Helsinki Helsinki Finland; ^3^ Department of Epidemiology and Public Health University College London London UK; ^4^ INSERM U1153 Epidemiology of Ageing and Neurodegenerative Diseases Université de Paris Paris France; ^5^ Institute for Molecular Medicine Finland (FIMM) University of Helsinki Helsinki Finland; ^6^ National Institute for Health and Welfare Helsinki Finland; ^7^ Department of Public Health University of Turku Turku Finland; ^8^ Turku University Hospital Turku Finland; ^9^ Finnish Institute of Occupational Health Helsinki Finland

**Keywords:** cohort studies, dementia, disease, hospitalization, risk factors

## Abstract

**Introduction:**

Conventional risk factors targeted by prevention (e.g., low education, smoking, and obesity) are associated with a 1.2‐ to 2‐fold increased risk of dementia. It is unclear whether having a physical disease is an equally important risk factor for dementia.

**Methods:**

In this exploratory multicohort study of 283,414 community‐dwelling participants, we examined 22 common hospital‐treated physical diseases as risk factors for dementia.

**Results:**

During a median follow‐up of 19 years, a total of 3416 participants developed dementia. Those who had erysipelas (hazard ratio = 1.82; 95% confidence interval = 1.53 to 2.17), hypothyroidism (1.94; 1.59 to 2.38), myocardial infarction (1.41; 1.20 to 1.64), ischemic heart disease (1.32; 1.18 to 1.49), cerebral infarction (2.44; 2.14 to 2.77), duodenal ulcers (1.88; 1.42 to 2.49), gastritis and duodenitis (1.82; 1.46 to 2.27), or osteoporosis (2.38; 1.75 to 3.23) were at a significantly increased risk of dementia. These associations were not explained by conventional risk factors or reverse causation.

**Discussion:**

In addition to conventional risk factors, several physical diseases may increase the long‐term risk of dementia.

## 1 INTRODUCTION

Because no treatment to stop or delay the progression of dementia is available, prevention remains the primary means to combat the disease.^1^, ^2^ The National Academies of Sciences, Engineering, and Medicine recommend three targets for dementia prevention: cognitive training, management of hypertension, and physical activity.^3^ The 2017 Lancet Commission,^1^ and more recently, the World Health Organization^4^ have highlighted further potentially modifiable risk factors, including low education, obesity, hearing loss, smoking, depression, social isolation, and diabetes. The associations between these risk factors and dementia are, however, relatively modest, with meta‐analyses reporting relative risk estimates of two or below.^1^, ^5^, ^6^, ^7^, ^8^, ^9^, ^10^, ^11^, ^12^

Many people with dementia are affected by multiple other physical diseases^13^, ^14^ that may also increase the risk of dementia, although they are not currently acknowledged as targets for dementia prevention. These include cardiovascular disease and some autoimmune, inflammatory, and infectious diseases.^15^, ^16^, ^17^, ^18^, ^19^ Genetic studies, supporting the role of these diseases in the development of dementia, have found shared genetic variants for lipid metabolism, the immune system, and Alzheimer's disease, a common form of dementia.^20^, ^21^ To date, however, few studies have systematically compared the magnitude of the dementia risk associated with conventional risk factors and physical diseases.

To address this limitation, we examined the association between 22 potentially dementia‐related physical diseases^22^ and incident all‐cause dementia before and after adjustment for conventional risk factors. Using a data‐driven approach, our aim was to generate new hypotheses for research on the prevention and etiology of dementia. To minimize ascertainment and reverse causation biases, we excluded the first 10 years of follow‐up in the sensitivity analyses.^10^

## 2 METHODS

### 2.1 Study population

This analysis is based on four prospective cohort studies linked to national health registries: the Finnish Public Sector study (FPS), the Health and Social Support study (HeSSup), and the Still Working study (STW) in Finland, and the Whitehall II study (WHII) in the UK. Of the 351,331 participants eligible for this study, we selected adults aged 18 years or older, who were successfully linked to hospitalization registers and free of known dementia at study entry: a total of 283,414 men and women (Figure A.1 and Methods A.1 in the Appendix). A local ethics committee approved data collection and analysis for all the studies (Methods A.1 in the Appendix).

**HIGHLIGHTS**

- Multicohort study of 22 physical diseases as long‐term risk factors for dementia
- Eight vascular, inflammatory, and bone diseases were linked to increased dementia risk
- The associations were as strong as those between standard risk factors and dementia

**RESEARCH IN CONTEXT**

1. **Systematic review**: The National Academies of Sciences, Engineering, and Medicine recommend cognitive training, management of hypertension, and physical activity for dementia prevention. Other major guidelines additionally recommend targeting low education, obesity, hearing loss, smoking, depression, social isolation, and diabetes. Although many physical diseases have been found to increase the risk of dementia, current guidelines recognize only three: hypertension, diabetes, and hearing loss.
2. **Interpretation**: This data‐driven multicohort study of 283,414 individuals followed up for a median of 19 years found eight physical diseases to be at least as strongly related to increased dementia risk as the conventional dementia risk factors. These diseases were erysipelas, hypothyroidism, myocardial infarction, chronic ischemic heart disease, cerebral infarction, duodenal ulcer, gastritis and duodenitis, and osteoporosis.
3. **Future directions**: Future research on underlying mechanisms would help evaluate whether these disease–dementia associations are causal.

### 2.2 Diagnoses of hospital‐treated diseases

**FIGURE 1:**
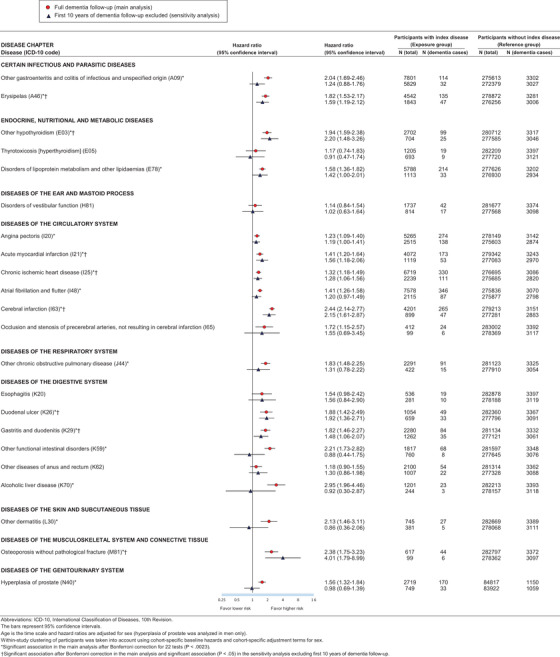
Hazard ratios for incident dementia by exposure to 22 hospital‐treated diseases in main analysis (full follow‐up) and in sensitivity analysis (the first 10 years of dementia follow‐up excluded). ICD‐10, International Classification of Diseases, 10th Revision. The bars represent 95% confidence intervals. Age is the time scale and hazard ratios are adjusted for sex (hyperplasia of prostate was analyzed in men only). Within‐study clustering of participants has been taken into account using cohort‐specific baseline hazards and cohort‐specific adjustment terms for sex. *Significant association in the main analysis after Bonferroni correction for 22 tests (*P* < .0023). ^†^Significant association after Bonferroni correction in the main analysis and significant association (*P* < .05) in the sensitivity analysis excluding first 10 years of dementia follow‐up.

We linked participants to the records of national health registers and retrieved both primary and secondary diagnoses from inpatient hospital discharge information using the International Classification of Diseases, 10th Revision (ICD‐10). The diagnosis codes from the 8th and 9th revisions (ICD‐8 and ICD‐9) were converted into the corresponding ICD‐10 codes (Table A.1 in the Appendix). To avoid false‐positive findings and publication bias, we systematically considered 32 physical diseases that were associated with an elevated 5‐year incidence of at least one type of dementia (or were on a disease trajectory later leading to dementia) in a hypothesis‐free Danish total population study with rigorous control for multiple testing.^22^ We excluded diseases in ICD‐10 Chapters V (Mental and behavioral disorders) and VI (Diseases of the nervous system) because their relation to dementia has already been studied extensively.^9^, ^23^, ^24^ We found sufficient case numbers in our data for 25 of these physical diseases (i.e., a minimum of three incident dementia cases that occurred more than 10 years after the diagnosis of the disease). Current prevention guidelines^1^, ^4^ acknowledge three of these diseases (type 1 and 2 diabetes and hypertension) as dementia risk factors, so we examined 22 other physical diseases as potential risk factors for dementia (Figure 1).

### 2.3 Covariates

We included the following covariates:^1^ age, sex, low education/socioeconomic status, hypertension, smoking, depression, physical inactivity, diabetes, marital status (a proxy for social isolation), obesity (available in all cohorts except STW), and apolipoprotein E (*APOE*) genotype (available in WHII). The definitions of these variables in each study are provided in Methods A.1 in the Appendix.

### 2.4 Ascertainment of incident dementia

We focused on all‐cause dementia because electronic health records may not be a reliable source of data to ascertain specific dementia subtypes. We compiled dementia diagnoses from hospital inpatient and outpatient records, medication reimbursement entitlements for the treatment of dementia, and causes of death, as available in each study between study baseline (1986 to 2005) and the end of follow‐up (2012 to 2017) (Methods A.1 in the Appendix). For anti‐dementia medication reimbursement entitlements, dementia diagnosis has to be made or confirmed in a neurology or geriatrics unit in secondary or tertiary health care or by a qualified neurologist or geriatrician (a prerequisite for reimbursement by the Finnish Social Insurance Institution).

In FPS, HeSSup, and STW, a diagnosis of dementia comprised ICD‐10 codes F00‐F03, F05.1, G30, G31.0, G31.1, and G31.8, whereas in WHII, it comprised ICD‐10 codes F00, F01, F03, G30, and G31. We defined the date of incident dementia as the first occurrence of dementia diagnosis, whether primary or secondary, in any of these information sources.

### 2.5 Statistical analysis

We computed hazard ratios for the associations of hospital‐treated physical diseases (index diseases) with dementia in separate Cox proportional hazards models. We used a one‐stage approach for a pooled analysis of individual‐level data from four cohort studies and took the within‐study clustering of participants into account using cohort‐specific baseline hazards and cohort‐specific adjustment terms for covariates.^25^ This means that individual‐level data from the four cohorts were analyzed in a single Cox model that was stratified by cohort. The model produced a single effect estimate for the index disease, but the effect estimates for adjusting variables (sex in all models and conventional dementia risk factors in further adjusted models) were allowed to vary from cohort to cohort. All Cox models were adjusted for age using age as the time scale.

Follow‐up for incident dementia started at study entry and continued until dementia diagnosis, death, or end of follow‐up, whichever came first. We modeled exposure to the index diseases using time‐dependent analysis whenever hospitalization from the index disease occurred after study entry (incident index disease).^26^ If hospitalization occurred before study entry (prevalent index disease), the participant contributed to the follow‐up as exposed from study entry. We also performed sensitivity analyses restricted to those free of the index disease at study entry and after excluding dementia cases that lacked information on the type of dementia. We examined the proportional hazards assumption using scaled Schoenfeld residuals (Methods A.2 and Figure A.2 in the Appendix). We analyzed early and late‐onset dementia as separate outcomes by splitting follow‐up at age 65 (those whose dementia onset was before age 65 vs those whose dementia onset was at or after age 65).

To reduce the risk of ascertainment bias and reverse causation, we repeated the analysis after excluding incident dementia cases that occurred during the first 10 years after initial hospitalization for the index disease (these biases mainly affect cases of dementia diagnosed early in the follow‐up).^10^, ^27^ Ascertainment bias arises when medical attention for one disease increases the probability of detecting another. Reverse causation bias occurs if the long preclinical phase of dementia increases the risk of the index disease.^17^, ^28^ For those not exposed to the index disease at study entry, we also excluded dementia cases occurring within the first 10 years of follow‐up (Figure A.3 in the Appendix).

We examined whether the index diseases were associated with dementia independently of each other by entering them into the same Cox model. Two diseases reflected coronary heart disease: myocardial infarction and chronic ischemic heart disease. To avoid over‐adjustment, these two diseases were not adjusted for each other.

We used the Fine‐Gray competing‐risks model to compute sub‐hazard ratios with death without dementia as the competing outcome event. The Fine‐Gray model was specified analogously with the Cox models. Age was the timescale, and the effect estimate for sex (the adjusting variable) was allowed to vary from cohort to cohort. We also adjusted the models for cohort and used robust standard errors that were clustered for cohort.

To compare the relative dementia risk related to physical diseases versus conventional potentially modifiable dementia risk factors (low education/socioeconomic status, hypertension, obesity, smoking, depression, physical inactivity, marital status, and diabetes), we computed the hazard ratios for these risk factors using the same modeling as for the index diseases. Because blood pressure and weight declines in preclinical dementia, we analyzed hypertension and obesity at middle age, excluding those aged 65 or older at baseline.^29^, ^30^ Analogously with the models for physical diseases, individual‐level data from the cohorts were analyzed in a single Cox model that produced a single effect estimate for the risk factor of interest and was stratified by cohort. Age was the timescale and the models were adjusted for sex, which was allowed to have different effect estimates for different cohorts.

To assess the effect of conventional dementia risk factors on the associations between physical diseases and dementia, we adjusted the Cox models for these factors, sex, and the statistically significant interactions between the adjusting variables (indicated by a likelihood‐ratio test with *P* < .05) in a subcohort with relevant data available (Table A.2 in the Appendix). One cohort (STW) was excluded from these analyses because no data on body mass index were available. As the main analysis, individual‐level data from the cohorts were analyzed in a single Cox model that was stratified by cohort. The model produced a single estimate for the disease of interest, but the effect estimates for adjusting variables (sex and the conventional potentially modifiable dementia risk factors) and the interactions between them were allowed to vary from cohort to cohort. The significant interactions were between sex and marital status, low education/socioeconomic status and physical inactivity, hypertension and marital status, obesity and smoking, and smoking and depression. We were only able to model the interaction between obesity and smoking for FPS and WHII, because HeSSup had no dementia cases among those who were both obese and smokers.

To examine the clustering of the index diseases that were associated with dementia, we computed odds ratios for the associations between these diseases by meta‐analyzing data from the four cohorts and using the Mantel‐Haenszel odds ratio method without continuity correction.^31^

We used Stata MP 15 and 16 to analyze the data. The syntax for these analyses is available in the Appendix (Methods A.3). We report all confidence intervals at a 95% confidence level.

## 3 RESULTS

Table 1 provides the participants’ demographic characteristics (for cohort‐specific characteristics, see Table A.3 and for characteristics split by the presence or absence of a physical disease, see Table A.4 in the Appendix). During the 5,488,175 person‐years at risk, we recorded 3416 incident cases of dementia. Of these, 1933 (56.6%) were diagnosed as Alzheimer's disease, 219 (6.4%) as vascular dementia, 5 (0.1%) as comorbid Alzheimer's disease and vascular dementia, and 908 (26.6%) as other dementias. For 351 cases (10.3%), no information on the type of dementia was available. We identified 1501 incident dementia cases from inpatient hospital discharge records, 814 from other hospital records, 1076 from anti‐dementia medication reimbursement entitlements, and 25 from death certificates. Of the dementia cases, 667 were early onset and 2749 late‐onset (Figure A.4 in the Appendix). Dementia incidence per 100,000 person‐years by age group was 13 for participants younger than 65, 213 for individuals aged 65 to 69, 559 for those aged 70 to 74, 1280 for those aged 75 to 79, 2364 for those aged 80 to 84, and 3760 for those aged 85 or older.

**TABLE 1:** Characteristics of participants at study entry

		No. (%)
Demographic		(N = 283,414)
Age at entry, years		
	18–39	181,942 (64.2%)
	40–49	59,866 (21.1%)
	50–59	36,610 (12.9%)
	60–87	4996 (1.8%)
Age at entry, median (range), years		33.5 (18.0–87.9)
Sex		
	Men	87,536 (30.9%)
	Women	195,878 (69.1%)
Education/socioeconomic status		
	Low	44,050 (15.5%)
	Intermediate	98,007 (34.6%)
	High	141,101 (49.8%)
	(not available)	256 (0.1%)
Follow‐up, median (range), years		19.0 (0.0–30.8)
Dementia by end of follow‐up		
	No	279,999 (98.8%)
	Yes	3415 (1.2%)
Age at dementia diagnosis, median (range), years		73.0 (22.7–93.2)

**FIGURE 2:**
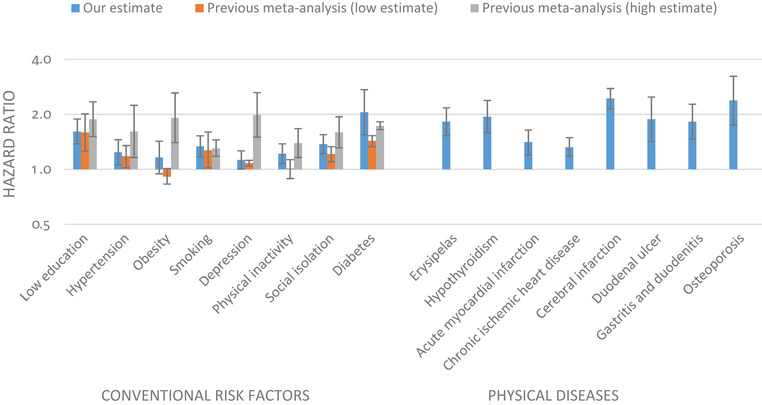
Hazard ratio for dementia for conventional risk factors versus physical diseases. The bars represent 95% confidence intervals. Numerical estimates are reported in Table A.6 in the Appendix and their references are provided in the Appendix (p. 99). Our estimates for physical diseases are the same as those from full dementia follow‐up in Figure 1. The low and high estimates for the hazard ratios for conventional risk factors are from 16 meta‐analyses.

Figure 1 shows the hazard ratios of dementia associated with the 22 physical diseases assessed. After Bonferroni correction, 17 of these diseases were associated with dementia in the full follow‐up (*P *< .0023), and for eight diseases, the association with dementia remained robust when we considered only incident dementia cases occurring from year 10 onward (Table A.5 in the Appendix shows summary estimates by ICD‐10 Chapter). These eight diseases were erysipelas, hypothyroidism, acute myocardial infarction, chronic ischemic heart disease, cerebral infarction, duodenal ulcer, gastritis and duodenitis, and osteoporosis. The hazard ratios for these diseases ranged between 1.3 and 2.4 and were thus comparable to or higher than the relative risk estimates for conventional potentially modifiable dementia risk factors (Figure 2 [Table A.6 in the Appendix shows detailed data]). Unlike for the other five diseases, the hazard of dementia was not proportional for hypothyroidism, cerebral infarction, or osteoporosis, as the hazard ratio for dementia declined with increasing age (Figure A.2 in the Appendix).

**FIGURE 3:**
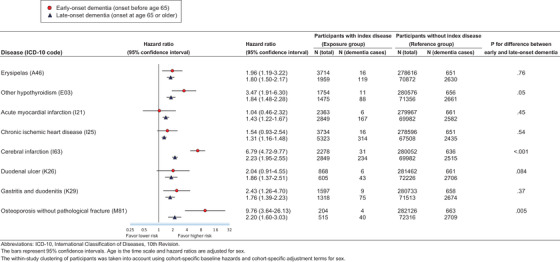
Hazard ratio for early onset and late‐onset dementia by exposure to eight physical diseases. ICD‐10, International Classification of Diseases, 10th Revision. The bars represent 95% confidence intervals. Age is the time scale and hazard ratios are adjusted for sex. The within‐study clustering of participants has been taken into account using cohort‐specific baseline hazards and cohort‐specific adjustment terms for sex.

As shown in Figure 3, cerebral infarction and osteoporosis were more strongly associated with early and late‐onset dementia (*P* for difference < .05), whereas the strength of the association for the remaining six physical diseases did not differ between early and late‐onset dementia. For comparison, the associations between conventional risk factors and dementia did not differ between early and late‐onset dementia (Table A.7 in the Appendix).

Table 2 shows that the disease–dementia associations were generally slightly attenuated when vascular dementia was excluded from dementia definition. The only exception was cerebral infarction, which was associated with a 2.4‐fold increased risk of dementia before but only a 1.8‐fold increased risk after exclusion. Fine‐Gray models, with mortality as the competing risk, revealed sub‐hazard ratios that were only slightly lower than the hazard ratios in the main analysis. Of the eight diseases, cardiovascular diseases (acute myocardial infarction, chronic ischemic heart disease, and cerebral infarction) were strongly clustered with each other (odds ratios 5.2 to 58.4) and duodenal ulcer was strongly associated with gastritis and duodenitis (odds ratio 12.7) (Figure A.5 in the Appendix). Despite this clustering, the association of each of the eight diseases with dementia remained after adjustment for the other diseases (Table 2).

**TABLE 2:** Sensitivity analyses for the associations of eight physical diseases with subsequent dementia

	Hazard ratio (95% CI)			Subhazard ratio (95% CI)
Disease (ICD‐10 code)	Main analysis (Cox model)	Excluding vascular dementia (Cox model)	Adjusted for physical diseases^a^ (Cox model)	Competing‐risks model (Fine‐Gray model)
Erysipelas (A46)	1.82 (1.53–2.17)	1.77 (1.48–2.13)	1.66 (1.40–1.98)	1.68 (1.41–2.00)
Other hypothyroidism (E03)	1.94 (1.59–2.38)	1.96 (1.53–2.52)	1.77 (1.44–2.17)	1.94 (1.66–2.28)
Acute myocardial infarction (I21)	1.41 (1.20–1.64)	1.36 (1.14–1.62)	1.27 (1.09–1.49)	1.34 (1.25–1.43)
Chronic ischemic heart disease (I25)	1.32 (1.18–1.49)	1.20 (1.05–1.38)	1.20 (1.06–1.35)	1.32 (1.18–1.47)
Cerebral infarction (I63)	2.44 (2.14–2.77)	1.82 (1.56–2.12)	2.27 (1.99–2.58)	2.18 (1.80–2.64)
Duodenal ulcer (K26)	1.88 (1.42–2.49)	1.84 (1.34–2.53)	1.72 (1.29–2.29)	1.57 (1.41–1.76)
Gastritis and duodenitis (K29)	1.82 (1.46–2.27)	1.64 (1.18–2.27)	1.61 (1.28–2.01)	1.62 (1.36–1.93)
Osteoporosis without pathological fracture (M81)	2.38 (1.75–3.23)	2.33 (1.52–3.60)	2.00 (1.47–2.71)	2.32 (1.85–2.90)

Abbreviations: CI, confidence interval; ICD‐10, International Classification of Diseases, 10th Revision.

Analyses are based on 283,414 participants of whom 3416 developed incident dementia, except for the model excluding vascular dementia, which is based on 273,381 participants of whom 2846 developed incident dementia.

Hazard ratios are adjusted for sex and age in the time scale.

The estimates from main analysis are the same as those from full dementia follow‐up in Figure 1.

a Additionally adjusted for the seven other physical diseases with robust long‐term association with dementia. (There were two diseases reflecting coronary heart disease: myocardial infarction and chronic ischemic heart disease. To avoid over‐adjustment, these two diseases were not adjusted for the other one.)

The disease–dementia associations were only slightly attenuated by adjustments for conventional dementia risk factors (low education/socioeconomic status, hypertension, obesity, smoking, depression, physical inactivity, marital status, and diabetes, and significant interactions between them) (Table 3). In a subcohort with relevant data available, adjustment for *APOE* genotype (no vs any ε4 allele) had little effect on hazard ratios (Table A.8 in the Appendix; note that numbers were insufficient for erysipelas). Associations with dementia were also replicated using only data on the primary reason for hospitalization to define the eight physical diseases, considering only incident hospitalizations from index diseases that occurred after study baseline, and after excluding cases with missing information on the type of dementia (Figure A.6‐8 in the Appendix).

**TABLE 3:** Association of eight physical diseases with subsequent dementia after adjustment for modifiable risk factors

	Hazard ratio (95% CI)	
Disease (ICD‐10 code)	Model A^a^	Model B^b^
Erysipelas (A46)	1.36 (0.78–2.36)	1.30 (0.74–2.26)
Other hypothyroidism (E03)	2.02 (1.42–2.87)	1.92 (1.35–2.73)
Acute myocardial infarction (I21)	1.44 (0.96–2.17)	1.32 (0.88–1.99)
Chronic ischemic heart disease (I25)	1.62 (1.27–2.07)	1.47 (1.15–1.88)
Cerebral infarction (I63)	3.34 (2.39–4.68)	3.05 (2.17–4.28)
Duodenal ulcer (K26)	2.19 (1.21–3.98)	1.83 (1.00–3.35)
Gastritis and duodenitis (K29)	2.07 (1.51–2.83)	1.89 (1.38–2.60)
Osteoporosis without pathological fracture (M81)	2.69 (1.75–4.15)	2.54 (1.64–3.94)

Abbreviations: CI, confidence interval; ICD‐10, International Classification of Diseases, 10th Revision.

All analyses are based on 121,696 participants without missing data on modifiable risk factors. Of these, 859 developed incident dementia during the follow‐up.

The within‐study clustering of participants was taken into account using cohort‐specific baseline hazards and cohort‐specific adjustment terms for covariates and interactions between them.

a Model A is adjusted for sex and age is the time scale.

b Model B is adjusted for sex and modifiable risk factors (low education/socioeconomic status, hypertension, obesity, smoking, depression, physical inactivity, marital status, and diabetes) and significant interactions between the adjusting variables. Age is the time scale.

## 4 DISCUSSION

In this exploratory multicohort study of over 280,000 adults, we quantified the associations of hospitalizations from 22 physical diseases with a subsequent risk of dementia during a 19‐year follow‐up. For eight diseases—including cardiovascular disease, inflammatory and infectious diseases, and bone disorders—the hazard ratios for incident dementia varied between 1.3 and 2.4 and were thus comparable to the relative risk estimates reported for conventional potentially modifiable dementia risk factors. The associations between the eight diseases and dementia were only slightly attenuated by adjustments for conventional dementia risk factors including low education/socioeconomic status, hypertension, obesity, smoking, depression, physical inactivity, marital status, and diabetes. The associations with incident dementia also remained when hospitalization for the disease occurred more than 10 years before the diagnosis of dementia.

To our knowledge, no long‐term follow‐ups of the associations between erysipelas, duodenal ulcer, gastritis, duodenitis, and dementia have been reported previously. However, a nationwide Danish study detected a higher risk of unspecified dementia or other degenerative diseases of the nervous system for erysipelas (risk ratio 1.2), duodenal ulcer (1.1), and gastritis and duodenitis (1.4) during a shorter 5‐year follow‐up.^22^ Earlier evidence of the association between hypothyroidism and dementia is also sparse.^32^, ^33^, ^34^, ^35^

A comparison of our findings on dementia‐related cardiovascular disease to those of studies that have included clinical examinations to ascertain dementia supports the validity of our approach. In the full follow‐up, our hazard ratios for acute myocardial infarction and chronic ischemic heart disease were 1.4 and 1.3, similar to the relative risk estimate of 1.3 for coronary heart disease and all‐cause dementia in a recent meta‐analysis.^16^ For cerebral infarction, our hazard ratio was 2.4, whereas it was 2.2 in the 2018 systematic review and meta‐analysis.^15^ The present finding on osteoporosis is also consistent with previous studies reporting an association between low bone mineral density and dementia.^36^, ^37^

Several proposed pathological mechanisms may explain the associations between physical diseases and increased dementia risk. According to genetic studies, the immune system may play a role in the etiology of Alzheimer's disease.^20^, ^21^ Animal studies suggest that systemic inflammation can increase the blood–brain barrier's permeability,^38^ induce inflammation into the central nervous system,^39^ and cause long‐term alterations in innate immune cells in the brain and an increased deposition of amyloid plaques, the hallmark of Alzheimer's pathology.^40^ Sydenham's chorea is a specific example of a post‐infectious autoimmune neurological disorder. It is a rare consequence of group A *Streptococcus* pharyngitis but is of interest here because erysipelas is also often caused by group A streptococci.^41^, ^42^ Inflammation may also contribute to increased dementia risk among those with duodenal ulcers, gastritis, and duodenitis. *Helicobacter pylori* infection is a shared cause of these diseases.^43^, ^44^ The evidence on the association between *H. pylori* infection and dementia is mixed^45^; it is possible that only the severe (hospital‐treated) forms of this infection are linked to the development of dementia.

The mechanisms underlying the observed association between osteoporosis and dementia are unclear. It has been suggested that low cumulative exposure to estrogen (a female sex hormone) is a common cause of osteoporosis and dementia,^36^ but findings on estrogen exposure and dementia are conflicting,^46^, ^47^ and osteoporosis has shown similar associations with dementia among men as among women.^37^ A further possibility, supported by the associations between osteoporosis and other diseases, is that osteoporosis represents a general indicator of poor health.

Plausible pathological mechanisms linking cardiovascular disease to dementia involve neuronal damage caused by microinfarcts, microbleeds, white matter lesions, and brain atrophy.^48^ We found that myocardial infarction and chronic ischemic heart disease were associated with an increased risk of dementia, even after adjustment for hypertension, which is an established vascular risk factor for dementia.^1^, ^3^, ^4^ In addition to direct neuronal damage, the association between cerebral infarction and dementia may reflect the impact of underlying systemic vascular disease. Little is known about the mechanisms underlying the association between hypothyroidism and dementia, although they could involve both direct effects on cognition and indirect effects through vascular pathology.^32^, ^33^, ^35^

Our study has some important strengths. We investigated, in a single analytic setting, 22 physical diseases that have been associated previously with a higher incidence of dementia in the short‐term (i.e., during the following 5 years), have been on a disease trajectory leading to dementia, or have been studied in separate studies with varying settings.^22^ In contrast to earlier analyses, we also examined long‐term associations that were less likely to be attributable to reverse causation and ascertainment biases.

Our study also has limitations and therefore should be considered hypothesis generating rather than conclusive. Although our main analysis of most diseases was well powered, the number of dementia cases among those exposed to the index diseases was low in some sensitivity analyses (e.g., occlusion and stenosis of precerebral arteries). For this reason, we may have missed diseases that are associated with an increased risk of dementia (Table A.9 in the Appendix). Given that the reproducibility of the findings was not confirmed in an independent study population, it also remains unclear whether our findings are replicable. We retrieved the information on the index diagnoses and incident dementia from electronic health records. This enabled all participants recruited for the study to be included in the analyses, rather than only those who continued to participate in follow‐up examinations. However, although electronic health records have high positive predictive value (false positives are unlikely), they miss mild and undiagnosed cases of diseases.^49^ This lack of sensitivity could contribute to the dilution of the results by nondifferential misclassification. The generalizability of our results is uncertain as the participants were mostly from occupational cohorts and therefore healthier than the general population. Our sample was relatively young, meaning a higher than usual proportion of early onset dementia cases. This may have led to an overestimation of the overall dementia risk related to cerebral infarction and osteoporosis, as these diseases had stronger associations with early than with late‐onset dementia. Due to reliance on observational data, our results may have been affected by confounding due to imprecise or lacking measurements of relevant covariates, such as conditions shared with the index disease (e.g., frailty) or off‐target effects of medications used to treat these diseases.

## 5 CONCLUSION

In this exploratory study, the associations between eight physical diseases (erysipelas, hypothyroidism, myocardial infarction, ischemic heart disease, cerebral infarction, duodenal ulcer, gastritis and duodenitis, and osteoporosis) and dementia were equally strong or stronger than those between several currently targeted risk factors (low education, hypertension, obesity, smoking, depression, physical inactivity, social isolation, and diabetes) and dementia. Further research on the mechanisms underlying these disease–dementia associations is needed to examine whether the observed associations are causal and to confirm the relevance of our findings for dementia etiology and prevention.

## Supporting information

Supplementary information
