# Supplementary material for: Long‐term risk of dementia following hospitalization due to physical diseases: A multicohort study
Source: Alzheimers Dement. 2020 Sep 4;16(12):1686–95. doi: 10.1002/alz.12167 (PMC7754402; doi:10.1002/alz.12167)
Supplement: Supplementary file 1 — Supplementary information [file ALZ-16-1686-s001.pdf]

Appendix

Sipilä PN, Lindbohm JV, Singh-Manoux A, Shipley MJ, Kiiskinen T, Havulinna AS, Vahtera J, Nyberg ST, Pentti J, Kivimäki M. Long-term risk for dementia following hospitalization due to physical diseases: a multicohort study

CONTENTS

Table A. 1. List of diseases in the study with their ICD-10 codes and the corresponding ICD-8 and ICD-9 codes.......... 2

Table A. 2. Characteristics of participants in the substudy without missing data on modifiable dementia risk factors..... 4

Table A. 3. Demographic characteristics of participants at study entry by cohort 6

Table A. 4. Demographic characteristics of participants at study entry by the presence or absence of physical disease .. 7

Table A. 5. Hazard ratios for incident dementia for exposure to 22 hospital-treated diseases in sensitivity analysis (the first 10 years of dementa follow-up excluded) and their ICD-10 chapter-specific summary estimates*.........................29

Table A. 6. Numerical estimates for Fig. 2 in the main text 31

Table A. 7. Association of conventional potentially modifiable dementia risk factors with early and late-onset dementia 32

Table A. 8. Association of hospital-treated diseases with subsequent dementia after adjustment for apolipoprotein e genotype 33

Table A. 9. Post hoc power analysis: hazard ratios observable with 90% probability at alpha = 0.05 34

Fig. A. 1. Selection of participants in the study 35

Fig. A. 2. Visualization of hazard ratios over time using exponentiated scaled Schoenfeld residuals 36

Fig. A. 3. Dementia follow-up 38

Part A. Dementia follow-up in the main analysis 38

Part B. Dementia follow-up from year 10 onwards...39

Fig. A. 4. Distribution of age at dementia diagnosis 40

Fig. A. 5. Odds ratios between hospital-treated diseases 41

Fig. A. 6. Hazard ratio for association of hospitalization due to disease versus no hospitalization due to disease with incident dementia ascertained using all diagnoses vs. primary diagnosis only 42

Fig. A. 7. Hazard ratio for association of hospitalization due to disease versus no hospitalization due to disease with incident dementia ascertained using all hospitalizations vs. incident hospitalization only 43

Fig. A. 8. Hazard ratio for association of hospitalization due to disease versus no hospitalization due to disease with incident dementia using data on all dementia cases vs. excluding dementia cases with unknown type of dementia 44

Methods A. 1. Study cohorts and data collection 45

The Finnish Public Sector study (FPS)..45

The Health and Social Support study (HeSSup) 46

The Still Working study (STW)..47

The Whitehall II study (WHII) 47

Methods A. 2. Proportionality of hazards...49

Methods A. 3. Stata code for data analysis 50

Supplementary references...99

Table A. 1. List of diseases in the study with their ICD-10 codes and the corresponding ICD-8 and ICD-9 codes

ICD-10 Code	ICD-10 Name	ICD-8 Codes	ICD-9 Codes
Dementia diagnoses			
F00	Dementia in Alzheimer's disease	29010	no equivalent
F01	Vascular dementia	no equivalent	4378A
F02	Dementia in other diseases classified elsewhere	no equivalent	no equivalent
F03	Unspecified dementia	29000, 29019	290, 2900A, 2941A
F05.1	Delirium superimposed on dementia	no equivalent	no equivalent
G30	Alzheimer's disease	no equivalent	3310A
G31.0	Circumscribed brain atrophy	29011, 34791	3311A
G31.1	Senile degeneration of brain, not elsewhere classified	34792	3312X
G31.8	Other specified degenerative diseases of nervous system	no equivalent	no equivalent
Index diagnoses			
A09	Other gastroenteritis and colitis of infectious and unspecified origin	00900, 00919, 00920, 00921, 00998	009, 0092A, 0093A
A46	Erysipelas	03599	035, 0350A, 0350B, 0350X
E03	Other hypothyroidism	24400, 24409	244, 2440A, 2441A, 2442A, 2443X, 2448A, 2448B, 2448X, 2449X
E05	Thyrotoxicosis [hyperthyroidism]	24200, 24209, 24210, 24220	242, 2420A, 2421A, 2422A, 2423X, 2424A, 2428X, 2429X
E10**	Insulin-dependent diabetes mellitus	no equivalent	2500B, 2501B, 2502B, 2503B, 2504B, 2505B, 2506B, 2507B, 2508B
E11**	Non-insulin-dependent diabetes mellitus	no equivalent	2500A, 2501A, 2502A, 2503A, 2504A, 2505A, 2506A, 2507A, 2508A
E14*,**	Unspecified diabetes mellitus	no equivalent	2500C, 2501C, 2502C, 2503C, 2504C, 2505C, 2506C, 2507C, 2508C
E16*	Other disorders of pancreatic internal secretion	25101, 25103, 25108, 25109	2510A, 2512A, 2512B, 2512X, 2514A, 2515A, 2518X, 2519X
E21*	Hyperparathyroidism and other disorders of parathyroid gland	25200, 25298, 25299	2520A, 2528X, 2529X
E78	Disorders of lipoprotein metabolism and other lipidemias	27200, 27201, 27288, 27299, 27900, 27901	2720A, 2720X, 2721A, 2721X, 2722A, 2723A, 2724A, 2724X, 2725A, 2728X, 2729X
H36*	Retinal disorders in diseases classified elsewhere	25002	3620A, 3620B

(continued)

Table A. 1. List of diseases in the study with their ICD-10 codes and the corresponding ICD-8 and ICD-9 codes (continued)

ICD-10 code	ICD-10 name	ICD-8 codes	ICD-9 codes
H81	Disorders of vestibular function	38599	3860A, 3861A, 3868X, 3869X
I10**	Essential (primary) hypertension	40199, 40299, 40399, 40499	401, 4019X
I20	Angina pectoris	41300, 41307, 41397, 41399	4110*, 4111*, 413, 4130*, 4131* (* = A,B,C,D,E,X)
I21	Acute myocardial infarction	no equivalent	no equivalent
I25	Chronic ischemic heart disease	41201, 41209, 41291, 41299, 41400, 41499	412, 4120*, 4121*, 4140*, 4148**, 4149** (*=A,B,C,D,E, X) (**=A,B)
I48	Atrial fibrillation and flutter	42792	4273A
I63	Cerebral infarction	no equivalent	4330A, 4331A, 4339A, 4349A
I64*	Stroke, not specified as hemorrhage or infarction	no equivalent	no equivalent
I65	Occlusion and stenosis of precerebral arteries, not resulting in cerebral infarction	no equivalent	4330X, 4331X, 4339X
J42*	Unspecified chronic bronchitis	49109	4919X
J44	Other chronic obstructive pulmonary disease	49104	4912A, 4912B, 4960A
K20	Esophagitis	53094	5301C, 5301D, 5301X
K26	Duodenal ulcer	53200, 53290, 53291, 53298, 53299	532, 5320*, 5321*, 5322*, 5323*, 5324*, 5325* (*=A,B,C,D,E,F, X)
K29	Gastritis and duodenitis	535* (*=00-09)	535, 5350A, 5351A, 5351B, 5351C, 5351D, 5351X, 5352A, 5353A, 5354X, 5355X, 5356X
K59	Other functional intestinal disorders	56102, 56400, 56498, 56499	5564A, 5582A, 5640A, 5645A, 5646A, 5647A, 5648X, 5649X
K62	Other diseases of anus and rectum	56902, 56903, 56904, 56906, 56907	5581A, 5690A, 5691A, 5692A, 5694X, 5781B
K70	Alcoholic liver disease	57100, 57101	5710A, 5711A, 5712A, 5713X
L30	Other dermatitis	69283, 69290	6929A, 6929B, 6958A, 6965A, 7058A
M81	Osteoporosis without pathological fracture	no equivalent	no equivalent
N31*	Neuromuscular dysfunction of bladder, not elsewhere classified	59601	5964A, 5965A, 5965B
N40	Hyperplasia of prostate	60000	600, 6000A

The diagnosis codes are from the Finnish national editions of the International Classification of Diseases, 8th, 9th, and 10th Revisions (ICD-8, ICD-9, and ICD-10).[1–3]

ICD-10 disease names are from: World Health Organization. International Statistical Classification of Diseases and Related Health Problems 10th Revision [Internet]. ICD-10 Version:2016. [cited 2 Oct 2018]. Available from: https://icd.who.int/browse10/2016/en

*Excluded from the current study due to insufficient data on the analyses of long-term dementia risk.

**Excluded from the current study because we regarded diabetes and hypertension as conventional dementia risk factors.

Table A. 2. Characteristics of participants in the substudy without missing data on modifiable dementia risk factors

Demographic		No. (%) Cohort FPS	HeSSup	STW	WHII	Total
Eligible for the substudy*		114,835	64,797	12,173	14,121	205,926
Included in the substudy		89,175	23,236	8980	9285	130,676
Age, years						
	18-39	33,197 (37.2%)	12,808 (55.1%)	4536 (50.5%)	0 (0.0%)	50,541 (38.7%)
	40-49	29,383 (32.9%)	5727 (24.6%)	2929 (32.6%)	1865 (20.1%)	39,904 (30.5%)
	50-59	23,866 (26.8%)	4701 (20.2%)	1467 (16.3%)	4654 (50.1%)	34,688 (26.5%)
	60-76	2729 (3.1%)	0 (0.0%)	48 (0.5%)	2766 (29.8%)	5543 (4.2%)
Age, median (range), years		43.8 (18.7-76.4)	33.5 (19.5-53.5)	39.7 (18.7-64.7)	55.2 (44.8-69.1)	43.5 (18.7-76.4)
Sex						
	Men	17,899 (20.1%)	9547 (41.1%)	6931 (77.2%)	6278 (67.6%)	40,655 (31.1%)
	Women	71,276 (79.9%)	13,689 (58.9%)	2049 (22.8%)	3007 (32.4%)	90,021 (68.9%)
Education/ socioeconomic status						
	Low	7655 (8.6%)	7014 (30.2%)	6188 (68.9%)	1973 (21.2%)	22,830 (17.5%)
	Intermediate	29,075 (32.6%)	12,384 (53.3%)	2145 (23.9%)	4530 (48.8%)	48,134 (36.8%)
	High	52,445 (58.8%)	3838 (16.5%)	647 (7.2%)	2782 (30.0%)	59,712 (45.7%)
Hypertension						
	No	83,502 (93.6%)	22,297 (96.0%)	8496 (94.6%)	8074 (87.0%)	122,369 (93.6%)
	Yes	5673 (6.4%)	939 (4.0%)	484 (5.4%)	1211 (13.0%)	8307 (6.4%)
Body mass index (kg/m2)						
	10.5-29.9	78,165 (87.7%)	20,986 (90.3%)	0 (0.0%)	8643 (93.1%)	107,794 (82.5%)
	30.0 or more	11,010 (12.3%)	2250 (9.7%)	0 (0.0%)	642 (6.9%)	13,902 (10.6%)
	(not available)	0 (0.0%)	0 (0.0%)	8980 (100%)	0 (0.0%)	8980 (6.9%)
Smoking status						
	Non-smoker	73,192 (82.1%)	17,257 (74.3%)	6021 (67.0%)	7628 (82.2%)	104,098 (79.7%)
	Current smoker	15,983 (17.9%)	5979 (25.7%)	2959 (33.0%)	1657 (17.8%)	26,578 (20.3%)

(continued)

Table A. 2. Characteristics of participants in the substudy without missing data on modifiable dementia risk factors (continued)

		No. (%)				
		Cohort				
**Demographic**		**FPS**	**HeSSup**	**STW**	**WHII**	**Total**
Physical inactivity						
	No	71,929 (80.7%)	18,447 (79.4%)	7229 (80.5%)	7882 (84.9%)	105,487 (80.7%)
	Yes	17,246 (19.3%)	4789 (20.6%)	1751 (19.5%)	1403 (15.1%)	25,189 (19.3%)
Married or cohabiting**						
	No	21,667 (24.3%)	7680 (33.1%)	2630 (29.3%)	2360 (25.4%)	34,337 (26.3%)
	Yes	67,508 (75.7%)	15,556 (66.9%)	6350 (70.7%)	6925 (74.6%)	96,339 (73.7%)
Diabetes						
	No	87,796 (98.5%)	23,009 (99.0%)	8898 (99.1%)	9017 (97.1%)	128,720 (98.5%)
	Yes	1379 (1.5%)	227 (1.0%)	82 (0.9%)	268 (2.9%)	1956 (1.5%)
No. of apolipoprotein E ε4 alleles						
	0	0 (0.0%)	0 (0.0%)	0 (0.0%)	4106 (44.2%)	4106 (3.1%)
	1 or 2	0 (0.0%)	0 (0.0%)	0 (0.0%)	1544 (16.6%)	1544 (1.2%)
	(not available)	89,175 (100%)	23,236 (100%)	8980 (100%)	3635 (39.1%)	125,026 (95.7%)
Follow-up, median (range), years		14.7 (0.0-19.6)	15.0 (0.7-15.0)	30.8 (0.0-30.8)	19.0 (0.0-20.0)	15.0 (0.0-30.8)
Dementia by the end of follow-up						
	No	88,687 (99.5%)	23,175 (99.7%)	8461 (94.2%)	8975 (96.7%)	129,298 (98.9%)
	Yes	488 (0.5%)	61 (0.3%)	519 (5.8%)	310 (3.3%)	1378 (1.1%)
Age at dementia diagnosis, median (range), years		66.9 (27.5-85.0)	60.6 (34.3-67.3)	75.3 (45.5-93.2)	76.6 (53.4-85.9)	72.4 (27.5-93.2)

Abbreviations: FPS, Finnish Public Sector study; HeSSup, Health and Social Support study; STW, Still Working study; WHII, Whitehall II study.

*Invited to the survey.

**In the Still Working study, the participants were classified as married vs. not.
Note: The Still Working study was excluded from analyses on body mass index and analyses adjusted for conventional potentially modifiable dementia risk factors, because no data on body mass index were available.

Table A. 3. Demographic characteristics of participants at study entry by cohort

Demographic		No. (%) Cohort FPS (N = 240,048)	HeSSup (N = 24,057)	STW (N = 9276)	WHII (N = 10,033)
Age at entry, years					
	18-39	164,072 (68.3%)	13,250 (55.1%)	4620 (49.8%)	0 (0.0%)
	40-49	48,930 (20.4%)	5919 (24.6%)	3037 (32.7%)	1980 (19.7%)
	50-59	25,155 (10.5%)	4888 (20.3%)	1566 (16.9%)	5001 (49.8%)
	60-87	1891 (0.8%)	0 (0.0%)	53 (0.6%)	3052 (30.4%)
Age at entry, median (range), years		32.1 (18.0-87.9)	33.5 (19.5-53.5)	40.7 (18.7-64.7)	55.4 (44.8-69.2)
Sex					
	Men	63,804 (26.6%)	9855 (41.0%)	7168 (77.3%)	6709 (66.9%)
	Women	176,244 (73.4%)	14,202 (59.0%)	2108 (22.7%)	3324 (33.1%)
Education/ socioeconomic status					
	Low	28,130 (11.7%)	7236 (30.1%)	6437 (69.4%)	2247 (22.4%)
	Intermediate	78,352 (32.6%)	12,663 (52.6%)	2176 (23.5%)	4816 (48.0%)
	High	133,566 (55.6%)	3902 (16.2%)	663 (7.1%)	2970 (29.6%)
	(not available)	0 (0.0%)	256 (1.1%)	0 (0.0%)	0 (0.0%)
Follow-up, median (range), years		20.0 (0.0-27.0)	15.0 (0.7-15.0)	30.8 (0.0-30.8)	19.0 (0.0-20.0)
Dementia by the end of follow-up					
	No	237,603 (99.0%)	23,987 (99.7%)	8726 (94.1%)	9682 (96.5%)
	Yes	2445 (1.0%)	70 (0.3%)	550 (5.9%)	351 (3.5%)
Age at dementia diagnosis, median (range), years		71.7 (22.7-92.2)	61.2 (34.3-67.3)	75.4 (45.5-93.2)	76.8 (53.4-85.9)

Abbreviations: FPS, Finnish Public Sector study; HeSSup, Health and Social Support study; STW, Still Working study; WHII, Whitehall II study.

Table A. 4. Demographic characteristics of participants at study entry by the presence or absence of physical disease

By other gastroenteritis and colitis of infectious and unspecified origin (A09)

Demographic		No. (%) No disease (N = 275,613)	Disease (N = 7801)
Age at entry, years			
	18-39	176,058 (63.9%)	5884 (75.4%)
	40-49	58,812 (21.3%)	1054 (13.5%)
	50-59	35,893 (13.0%)	717 (9.2%)
	60-87	4850 (1.8%)	146 (1.9%)
Age at entry, median (range), years		33.6 (18.0-87.9)	28.2 (18.0-74.2)
Sex			
	Men	84,047 (30.5%)	3489 (44.7%)
	Women	191,566 (69.5%)	4312 (55.3%)
Education/ socioeconomic status			
	Low	42,879 (15.6%)	1171 (15.0%)
	Intermediate	95,262 (34.6%)	2745 (35.2%)
	High	137,218 (49.8%)	3883 (49.8%)
	(not available)	254 (0.1%)	2 (0.0%)
Follow-up, median (range), years		19.0 (0.0-30.8)	18.6 (0.5-30.8)
Dementia by the end of follow-up			
	No	272,311 (98.8%)	7687 (98.5%)
	Yes	3302 (1.2%)	114 (1.5%)
Age at dementia diagnosis, median (range), years		72.9 (22.7-93.2)	75.8 (32.5-92.2)

By erysipelas (A46)			
		No. (%)	
Demographic		No disease (N = 278,872)	Disease (N = 4542)
Age at entry, years			
	18-39	179,954 (64.5%)	1988 (43.8%)
	40-49	58,418 (20.9%)	1448 (31.9%)
	50-59	35,589 (12.8%)	1021 (22.5%)
	60-87	4911 (1.8%)	85 (1.9%)
Age at entry, median (range), years		33.4 (18.0-87.9)	41.8 (18.0-74.2)
Sex			
	Men	85,468 (30.6%)	2068 (45.5%)
	Women	193,404 (69.4%)	2474 (54.5%)
Education/ socioeconomic status			
	Low	42819 (15.4%)	1231 (27.1%)
	Intermediate	96,325 (34.5%)	1682 (37.0%)
	High	139,472 (50.0%)	1629 (35.9%)
	(not available)	256 (0.1%)	0 (0.0%)
Follow-up, median (range), years		19.0 (0.0-30.8)	21.0 (0.6-30.8)
Dementia by the end of follow-up			
	No	275,591 (98.8%)	4407 (97.0%)
	Yes	3281 (1.2%)	135 (3.0%)
Age at dementia diagnosis, median (range), years		72.9 (22.7-93.2)	74.1 (45.4-91.1)

By other hypothyroidism (E03)			
		No. (%)	
Demographic		No disease (N = 280,712)	Disease (N = 2702)
Age at entry, years			
	18-39	180,949 (64.5%)	993 (36.8%)
	40-49	59,146 (21.1%)	720 (26.6%)
	50-59	35,898 (12.8%)	712 (26.4%)
	60-87	4719 (1.7%)	277 (10.3%)
Age at entry, median (range), years		33.4 (18.0-87.9)	45.4 (18.0-76.2)
Sex			
	Men	87,178 (31.1%)	358 (13.2%)
	Women	193,534 (68.9%)	2344 (86.8%)
Education/ socioeconomic status			
	Low	43,353 (15.4%)	697 (25.8%)
	Intermediate	97,027 (34.6%)	980 (36.3%)
	High	140,077 (49.9%)	1024 (37.9%)
	(not available)	255 (0.1%)	1 (0.0%)
Follow-up, median (range), years		19.0 (0.0-30.8)	19.9 (0.7-30.8)
Dementia by the end of follow-up			
	No	277,395 (98.8%)	2603 (96.3%)
	Yes	3317 (1.2%)	99 (3.7%)
Age at dementia diagnosis, median (range), years		72.9 (22.7-93.2)	75.2 (45.3-89.6)

By thyrotoxicosis [hyperthyroidism] (E05)			
		No. (%)	
Demographic		No disease (N = 282,209)	Disease (N = 1205)
Age at entry, years			
	18-39	181,258 (64.2%)	684 (56.8%)
	40-49	59,565 (21.1%)	301 (25.0%)
	50-59	36,421 (12.9%)	189 (15.7%)
	60-87	4965 (1.8%)	31 (2.6%)
Age at entry, median (range), years		33.5 (18.0-87.9)	36.8 (18.1-74.2)
Sex			
	Men	87,408 (31.0%)	128 (10.6%)
	Women	194,801 (69.0%)	1077 (89.4%)
Education/ socioeconomic status			
	Low	43,835 (15.5%)	215 (17.8%)
	Intermediate	97,567 (34.6%)	440 (36.5%)
	High	140,553 (49.8%)	548 (45.5%)
	(not available)	254 (0.1%)	2 (0.2%)
Follow-up, median (range), years		19.0 (0.0-30.8)	20.0 (0.5-30.8)
Dementia by the end of follow-up			
	No	278,812 (98.8%)	1186 (98.4%)
	Yes	3397 (1.2%)	19 (1.6%)
Age at dementia diagnosis, median (range), years		73.0 (22.7-93.2)	74.2 (53.9-83.7)

By disorders of lipoprotein metabolism and other lipidaemias (E78)			
		No. (%)	
**Demographic**		**No disease (N = 277,626)**	**Disease (N = 5788)**
Age at entry, years			
	18-39	180,933 (65.2%)	1009 (17.4%)
	40-49	58,181 (21.0%)	1685 (29.1%)
	50-59	34,373 (12.4%)	2237 (38.6%)
	60-87	4139 (1.5%)	857 (14.8%)
Age at entry, median (range), years		33.1 (18.0-87.9)	50.7 (18.1-76.2)
Sex			
	Men	84,530 (30.4%)	3006 (51.9%)
	Women	193,096 (69.6%)	2782 (48.1%)
Education/ socioeconomic status			
	Low	42,317 (15.2%)	1733 (29.9%)
	Intermediate	95,800 (34.5%)	2207 (38.1%)
	High	139,255 (50.2%)	1846 (31.9%)
	(not available)	254 (0.1%)	2 (0.0%)
Follow-up, median (range), years		19.0 (0.0-30.8)	19.5 (0.6-30.8)
Dementia by the end of follow-up			
	No	274,424 (98.8%)	5574 (96.3%)
	Yes	3202 (1.2%)	214 (3.7%)
Age at dementia diagnosis, median (range), years		72.7 (22.7-93.2)	76.9 (47.6-91.1)

By disorders of vestibular function (H81)

Demographic		No. (%) No disease (N = 281,677)	Disease (N = 1737)
Age at entry, years			
	18-39	181,251 (64.3%)	691 (39.8%)
	40-49	59,302 (21.1%)	564 (32.5%)
	50-59	36,171 (12.8%)	439 (25.3%)
	60-87	4953 (1.8%)	43 (2.5%)
Age at entry, median (range), years		33.5 (18.0-87.9)	43.5 (18.0-72.0)
Sex			
	Men	87,078 (30.9%)	458 (26.4%)
	Women	194,599 (69.1%)	1279 (73.6%)
Education/ socioeconomic status			
	Low	43,670 (15.5%)	380 (21.9%)
	Intermediate	97,404 (34.6%)	603 (34.7%)
	High	140,347 (49.8%)	754 (43.4%)
	(not available)	256 (0.1%)	0 (0.0%)
Follow-up, median (range), years		19.0 (0.0-30.8)	21.0 (2.7-30.8)
Dementia by the end of follow-up			
	No	278,303 (98.8%)	1695 (97.6%)
	Yes	3374 (1.2%)	42 (2.4%)
Age at dementia diagnosis, median (range), years		73.0 (22.7-93.2)	76.2 (60.6-87.3)

Demographic	By angina pectoris (I20)	No. (%) No disease (N = 278,149)	Disease (N = 5265)
Age at entry, years			
	18-39	181,188 (65.1%)	754 (14.3%)
	40-49	58,103 (20.9%)	1763 (33.5%)
	50-59	34,411 (12.4%)	2199 (41.8%)
	60-87	4447 (1.6%)	549 (10.4%)
Age at entry, median (range), years		33.1 (18.0-87.9)	50.5 (18.0-80.7)
Sex			
	Men	84,291 (30.3%)	3245 (61.6%)
	Women	193,858 (69.7%)	2020 (38.4%)
Education/ socioeconomic status			
	Low	42,034 (15.1%)	2016 (38.3%)
	Intermediate	96,202 (34.6%)	1805 (34.3%)
	High	139,657 (50.2%)	1444 (27.4%)
	(not available)	256 (0.1%)	0 (0.0%)
Follow-up, median (range), years		19.0 (0.0-30.8)	21.0 (0.4-30.8)
Dementia by the end of follow-up			
	No	275,007 (98.9%)	4991 (94.8%)
	Yes	3142 (1.1%)	274 (5.2%)
Age at dementia diagnosis, median (range), years		72.7 (22.7-93.2)	76.8 (55.0-90.0)

Demographic	By myocardial infarction (I21)	No. (%) No disease (N = 279,342)	Disease (N = 4072)
Age at entry, years			
	18-39	181,108 (64.8%)	834 (20.5%)
	40-49	58,464 (20.9%)	1402 (34.4%)
	50-59	35,112 (12.6%)	1498 (36.8%)
	60-87	4658 (1.7%)	338 (8.3%)
Age at entry, median (range), years		33.3 (18.0-87.9)	49.1 (18.0-80.7)
Sex			
	Men	84,969 (30.4%)	2567 (63.0%)
	Women	194,373 (69.6%)	1505 (37.0%)
Education/ socioeconomic status			
	Low	42,485 (15.2%)	1565 (38.4%)
	Intermediate	96,607 (34.6%)	1400 (34.4%)
	High	139,996 (50.1%)	1105 (27.1%)
	(not available)	254 (0.1%)	2 (0.0%)
Follow-up, median (range), years		19.0 (0.0-30.8)	22.0 (0.6-30.8)
Dementia by the end of follow-up			
	No	276,099 (98.8%)	3899 (95.8%)
	Yes	3243 (1.2%)	173 (4.2%)
Age at dementia diagnosis, median (range), years		72.7 (22.7-92.2)	76.9 (51.8-93.2)

By chronic ischemic heart disease (I25)		No. (%)	
Demographic		No disease (N = 276,695)	Disease (N = 6719)
Age at entry, years			
	18-39	181,044 (65.4%)	898 (13.4%)
	40-49	57,811 (20.9%)	2055 (30.6%)
	50-59	33,721 (12.2%)	2889 (43.0%)
	60-87	4119 (1.5%)	877 (13.1%)
Age at entry, median (range), years		33.0 (18.0-87.9)	51.2 (18.0-76.2)
Sex			
	Men	83,240 (30.1%)	4296 (63.9%)
	Women	193,455 (69.9%)	2423 (36.1%)
Education/ socioeconomic status			
	Low	41,518 (15.0%)	2532 (37.7%)
	Intermediate	95,675 (34.6%)	2332 (34.7%)
	High	139,247 (50.3%)	1854 (27.6%)
	(not available)	255 (0.1%)	1 (0.0%)
Follow-up, median (range), years		19.0 (0.0-30.8)	21.0 (0.1-30.8)
Dementia by the end of follow-up			
	No	273,609 (98.9%)	6389 (95.1%)
	Yes	3086 (1.1%)	330 (4.9%)
Age at dementia diagnosis, median (range), years		72.6 (22.7-92.2)	76.7 (45.5-93.2)

Demographic		By atrial fibrillation and flutter (I48) No. (%) No disease (N = 275,836)	Disease (N = 7578)
Age at entry, years			
	18-39	180,724 (65.5%)	1218 (16.1%)
	40-49	57,580 (20.9%)	2286 (30.2%)
	50-59	33,406 (12.1%)	3204 (42.3%)
	60-87	4126 (1.5%)	870 (11.5%)
Age at entry, median (range), years		33.0 (18.0-87.9)	50.8 (18.1-73.8)
Sex			
	Men	83,324 (30.2%)	4212 (55.6%)
	Women	192,512 (69.8%)	3366 (44.4%)
Education/ socioeconomic status			
	Low	41,570 (15.1%)	2480 (32.7%)
	Intermediate	95,499 (34.6%)	2508 (33.1%)
	High	138,515 (50.2%)	2586 (34.1%)
	(not available)	252 (0.1%)	4 (0.1%)
Follow-up, median (range), years		19.0 (0.0-30.8)	21.0 (0.4-30.8)
Dementia by the end of follow-up			
	No	272,766 (98.9%)	7232 (95.4%)
	Yes	3070 (1.1%)	346 (4.6%)
Age at dementia diagnosis, median (range), years		72.4 (22.7-93.2)	77.6 (55.5-92.2)

Demographic	By cerebral infarction (I63)	No. (%) No disease (N = 279,213)	Disease (N = 4201)
Age at entry, years			
	18-39	181,027 (64.8%)	915 (21.8%)
	40-49	58,464 (20.9%)	1402 (33.4%)
	50-59	35,018 (12.5%)	1592 (37.9%)
	60-87	4704 (1.7%)	292 (7.0%)
Age at entry, median (range), years		33.3 (18.0-87.9)	48.9 (18.1-85.9)
Sex			
	Men	85,500 (30.6%)	2036 (48.5%)
	Women	193,713 (69.4%)	2165 (51.5%)
Education/ socioeconomic status			
	Low	42,504 (15.2%)	1546 (36.8%)
	Intermediate	96,628 (34.6%)	1379 (32.8%)
	High	139,828 (50.1%)	1273 (30.3%)
	(not available)	253 (0.1%)	3 (0.1%)
Follow-up, median (range), years		19.0 (0.0-30.8)	21.9 (0.6-30.8)
Dementia by the end of follow-up			
	No	276,062 (98.9%)	3936 (93.7%)
	Yes	3151 (1.1%)	265 (6.3%)
Age at dementia diagnosis, median (range), years		72.8 (22.7-93.2)	74.8 (47.9-91.0)

By occlusion and stenosis of precerebral arteries, not resulting in cerebral infarction (I65)			
		**No. (%)**	
**Demographic**		**No disease (N = 283,002)**	**Disease (N = 412)**
Age at entry, years			
	18-39	181,879 (64.3%)	63 (15.3%)
	40-49	59,740 (21.1%)	126 (30.6%)
	50-59	36,460 (12.9%)	150 (36.4%)
	60-87	4923 (1.7%)	73 (17.7%)
Age at entry, median (range), years		33.5 (18.0-87.9)	51.2 (18.1-77.9)
Sex			
	Men	87,292 (30.8%)	244 (59.2%)
	Women	195,710 (69.2%)	168 (40.8%)
Education/ socioeconomic status			
	Low	43,898 (15.5%)	152 (36.9%)
	Intermediate	97,859 (34.6%)	148 (35.9%)
	High	140,989 (49.8%)	112 (27.2%)
	(not available)	256 (0.1%)	0 (0.0%)
Follow-up, median (range), years		19.0 (0.0-30.8)	19.9 (1.7-30.8)
Dementia by the end of follow-up			
	No	279,610 (98.8%)	388 (94.2%)
	Yes	3392 (1.2%)	24 (5.8%)
Age at dementia diagnosis, median (range), years		73.0 (22.7-93.2)	75.6 (67.3-86.1)

By other chronic obstructive pulmonary disease (J44)			
		**No. (%)**	
**Demographic**		**No disease (N = 281,123)**	**Disease (N = 2291)**
Age at entry, years			
	18-39	181,559 (64.6%)	383 (16.7%)
	40-49	59,124 (21.0%)	742 (32.4%)
	50-59	35,730 (12.7%)	880 (38.4%)
	60-87	4710 (1.7%)	286 (12.5%)
Age at entry, median (range), years		33.4 (18.0-87.9)	50.2 (18.1-74.8)
Sex			
	Men	86,350 (30.7%)	1186 (51.8%)
	Women	194,773 (69.3%)	1105 (48.2%)
Education/ socioeconomic status			
	Low	42,936 (15.3%)	1114 (48.6%)
	Intermediate	97,189 (34.6%)	818 (35.7%)
	High	140,745 (50.1%)	356 (15.5%)
	(not available)	253 (0.1%)	3 (0.1%)
Follow-up, median (range), years		19.0 (0.0-30.8)	19.7 (1.1-30.8)
Dementia by the end of follow-up			
	No	277,798 (98.8%)	2200 (96.0%)
	Yes	3325 (1.2%)	91 (4.0%)
Age at dementia diagnosis, median (range), years		73.0 (22.7-93.2)	75.4 (27.5-86.7)

Demographic	By esophagitis (K20)	No. (%) No disease (N = 282,878)	Disease (N = 536)
Age at entry, years			
	18-39	181,821 (64.3%)	121 (22.6%)
	40-49	59,739 (21.1%)	127 (23.7%)
	50-59	36,419 (12.9%)	191 (35.6%)
	60-87	4899 (1.7%)	97 (18.1%)
Age at entry, median (range), years		33.5 (18.0-87.9)	51.0 (18.2-68.0)
Sex			
	Men	87,257 (30.8%)	279 (52.1%)
	Women	195,621 (69.2%)	257 (47.9%)
Education/ socioeconomic status			
	Low	43,881 (15.5%)	169 (31.5%)
	Intermediate	97,769 (34.6%)	238 (44.4%)
	High	140,972 (49.8%)	129 (24.1%)
	(not available)	256 (0.1%)	0 (0.0%)
Follow-up, median (range), years		19.0 (0.0-30.8)	19.0 (1.7-30.8)
Dementia by the end of follow-up			
	No	279,481 (98.8%)	517 (96.5%)
	Yes	3397 (1.2%)	19 (3.5%)
Age at dementia diagnosis, median (range), years		73.0 (22.7-93.2)	75.7 (52.8-84.6)

Demographic	By duodenal ulcer (K26)	No. (%) No disease (N = 282,360)	Disease (N = 1054)
Age at entry, years			
	18-39	181,651 (64.3%)	291 (27.6%)
	40-49	59,501 (21.1%)	365 (34.6%)
	50-59	36,290 (12.9%)	320 (30.4%)
	60-87	4918 (1.7%)	78 (7.4%)
Age at entry, median (range), years		33.5 (18.0-87.9)	46.7 (18.7-76.8)
Sex			
	Men	86,858 (30.8%)	678 (64.3%)
	Women	195,502 (69.2%)	376 (35.7%)
Education/ socioeconomic status			
	Low	43,626 (15.5%)	424 (40.2%)
	Intermediate	97,641 (34.6%)	366 (34.7%)
	High	140,837 (49.9%)	264 (25.0%)
	(not available)	256 (0.1%)	0 (0.0%)
Follow-up, median (range), years		19.0 (0.0-30.8)	21.0 (0.4-30.8)
Dementia by the end of follow-up			
	No	278,993 (98.8%)	1005 (95.4%)
	Yes	3367 (1.2%)	49 (4.6%)
Age at dementia diagnosis, median (range), years		73.0 (22.7-93.2)	73.4 (53.9-89.5)

Demographic	By gastritis and duodenitis (K29)	No. (%) No disease (N = 281,134)	Disease (N = 2280)
Age at entry, years			
	18-39	181,233 (64.5%)	709 (31.1%)
	40-49	59,327 (21.1%)	539 (23.6%)
	50-59	35,922 (12.8%)	688 (30.2%)
	60-87	4652 (1.7%)	344 (15.1%)
Age at entry, median (range), years		33.4 (18.0-87.9)	48.8 (18.2-68.0)
Sex			
	Men	86,365 (30.7%)	1171 (51.4%)
	Women	194,769 (69.3%)	1109 (48.6%)
Education/ socioeconomic status			
	Low	43,333 (15.4%)	717 (31.4%)
	Intermediate	97,101 (34.5%)	906 (39.7%)
	High	140,444 (50.0%)	657 (28.8%)
	(not available)	256 (0.1%)	0 (0.0%)
Follow-up, median (range), years		19.0 (0.0-30.8)	19.0 (0.4-30.8)
Dementia by the end of follow-up			
	No	277,802 (98.8%)	2196 (96.3%)
	Yes	3332 (1.2%)	84 (3.7%)
Age at dementia diagnosis, median (range), years		73.0 (22.7-93.2)	74.4 (50.9-88.3)

Demographic		By other functional intestinal disorders (K59)<br>No. (%)<br>No disease<br>(N = 281,597)	Disease<br>(N = 1817)
Age at entry, years			
	18-39	181,239 (64.4%)	703 (38.7%)
	40-49	59,497 (21.1%)	369 (20.3%)
	50-59	36,113 (12.8%)	497 (27.4%)
	60-87	4748 (1.7%)	248 (13.6%)
Age at entry, median (range), years		33.5 (18.0-85.9)	46.7 (18.0-87.9)
Sex			
	Men	86,845 (30.8%)	691 (38.0%)
	Women	194,752 (69.2%)	1126 (62.0%)
Education/<br>socioeconomic status			
	Low	43,597 (15.5%)	453 (24.9%)
	Intermediate	97,303 (34.6%)	704 (38.7%)
	High	140,443 (49.9%)	658 (36.2%)
	(not available)	254 (0.1%)	2 (0.1%)
Follow-up, median (range), years		19.0 (0.0-30.8)	19.0 (0.3-30.8)
Dementia by the end of follow-up			
	No	278,249 (98.8%)	1749 (96.3%)
	Yes	3348 (1.2%)	68 (3.7%)
Age at dementia diagnosis, median (range), years		72.9 (22.7-93.2)	75.3 (53.6-89.1)

Demographic	By other diseases of anus and rectum (K62)	No. (%) No disease (N = 281,314)	Disease (N = 2100)
Age at entry, years			
	18-39	181,279 (64.4%)	663 (31.6%)
	40-49	59,322 (21.1%)	544 (25.9%)
	50-59	35,961 (12.8%)	649 (30.9%)
	60-87	4752 (1.7%)	244 (11.6%)
Age at entry, median (range), years		33.5 (18.0-87.9)	48.0 (18.0-76.2)
Sex			
	Men	86,651 (30.8%)	885 (42.1%)
	Women	194,663 (69.2%)	1215 (57.9%)
Education/ socioeconomic status			
	Low	43,613 (15.5%)	437 (20.8%)
	Intermediate	97,175 (34.5%)	832 (39.6%)
	High	140,271 (49.9%)	830 (39.5%)
	(not available)	255 (0.1%)	1 (0.0%)
Follow-up, median (range), years		19.0 (0.0-30.8)	19.1 (1.5-30.8)
Dementia by the end of follow-up			
	No	277,952 (98.8%)	2046 (97.4%)
	Yes	3362 (1.2%)	54 (2.6%)
Age at dementia diagnosis, median (range), years		73.0 (22.7-93.2)	75.0 (27.3-89.1)

Demographic	By alcoholic liver disease (K70)	No. (%)<br>No disease<br>(N = 282,213)	Disease<br>(N = 1201)
Age at entry, years			
	18-39	181,494 (64.3%)	448 (37.3%)
	40-49	59,389 (21.0%)	477 (39.7%)
	50-59	36,354 (12.9%)	256 (21.3%)
	60-87	4976 (1.8%)	20 (1.7%)
Age at entry, median (range), years		33.5 (18.0-87.9)	42.8 (18.1-67.6)
Sex			
	Men	86,804 (30.8%)	732 (60.9%)
	Women	195,409 (69.2%)	469 (39.1%)
Education/ socioeconomic status			
	Low	43,590 (15.4%)	460 (38.3%)
	Intermediate	97,568 (34.6%)	439 (36.6%)
	High	140,799 (49.9%)	302 (25.1%)
	(not available)	256 (0.1%)	0 (0.0%)
Follow-up, median (range), years		19.0 (0.0-30.8)	17.0 (0.2-30.8)
Dementia by the end of follow-up			
	No	278,820 (98.8%)	1178 (98.1%)
	Yes	3393 (1.2%)	23 (1.9%)
Age at dementia diagnosis, median (range), years		73.1 (22.7-93.2)	66.4 (52.3-83.4)

Demographic	By other dermatitis (L30)	No. (%)<br>No disease<br>(N = 282,669)	 Disease<br>(N = 745)
Age at entry, years			
	18-39	181,647 (64.3%)	295 (39.6%)
	40-49	59,648 (21.1%)	218 (29.3%)
	50-59	36,413 (12.9%)	197 (26.4%)
	60-87	4961 (1.8%)	35 (4.7%)
Age at entry, median (range), years		33.5 (18.0-87.9)	43.2 (18.1-67.9)
Sex			
	Men	87,195 (30.8%)	341 (45.8%)
	Women	195,474 (69.2%)	404 (54.2%)
Education/<br>socioeconomic status			
	Low	43,826 (15.5%)	224 (30.1%)
	Intermediate	97,724 (34.6%)	283 (38.0%)
	High	140,863 (49.8%)	238 (31.9%)
	(not available)	256 (0.1%)	0 (0.0%)
Follow-up, median (range), years		19.0 (0.0-30.8)	20.0 (1.5-30.8)
Dementia by the end of follow-up			
	No	279,280 (98.8%)	718 (96.4%)
	Yes	3389 (1.2%)	27 (3.6%)
Age at dementia diagnosis, median (range), years		73.0 (22.7-93.2)	75.5 (44.9-87.3)

Demographic		By osteoporosis without pathological fracture (M81) No. (%) No disease (N = 282,797)	Disease (N = 617)
Age at entry, years			
	18-39	181,868 (64.3%)	74 (12.0%)
	40-49	59,743 (21.1%)	123 (19.9%)
	50-59	36,368 (12.9%)	242 (39.2%)
	60-87	4818 (1.7%)	178 (28.8%)
Age at entry, median (range), years		33.5 (18.0-87.9)	54.7 (18.0-68.2)
Sex			
	Men	87,396 (30.9%)	140 (22.7%)
	Women	195,401 (69.1%)	477 (77.3%)
Education/ socioeconomic status			
	Low	43,810 (15.5%)	240 (38.9%)
	Intermediate	97,781 (34.6%)	226 (36.6%)
	High	140,950 (49.8%)	151 (24.5%)
	(not available)	256 (0.1%)	0 (0.0%)
Follow-up, median (range), years		19.0 (0.0-30.8)	19.0 (1.9-30.8)
Dementia by the end of follow-up			
	No	279,425 (98.8%)	573 (92.9%)
	Yes	3372 (1.2%)	44 (7.1%)
Age at dementia diagnosis, median (range), years		72.9 (22.7-93.2)	78.1 (55.5-89.3)

Demographic		By hyperplasia of prostate (N40) No. (%) No disease (N = 84,818)	Disease (N = 2718)
Age at entry, years			
	18-39	52,066 (61.4%)	196 (7.2%)
	40-49	17,952 (21.2%)	781 (28.7%)
	50-59	12,647 (14.9%)	1266 (46.6%)
	60-87	2153 (2.5%)	475 (17.5%)
Age at entry, median (range), years		34.3 (18.0-80.7)	52.5 (22.5-76.8)
Sex			
	Men	84,818 (100.0%)	2718 (100.0%)
	Women	0 (0.0%)	0 (0.0%)
Education/ socioeconomic status			
	Low	18,019 (21.2%)	792 (29.1%)
	Intermediate	31,158 (36.7%)	905 (33.3%)
	High	35,580 (41.9%)	1020 (37.5%)
	(not available)	61 (0.1%)	1 (0.0%)
Follow-up, median (range), years		19.0 (0.0-30.8)	20.0 (0.4-30.8)
Dementia by the end of follow-up			
	No	83,668 (98.6%)	2548 (93.7%)
	Yes	1150 (1.4%)	170 (6.3%)
Age at dementia diagnosis, median (range), years		73.3 (22.7-93.2)	76.9 (58.1-89.5)

Table A. 5. Hazard ratios for incident dementia for exposure to 22 hospital-treated diseases in sensitivity analysis (the first 10 years of dementa follow-up excluded) and their ICD-10 chapter-specific summary estimates*

**DISEASE CHAPTER**<br>**Disease (ICD-10 code)**	**Hazard ratio**<br>**(95% CI)**
**CERTAIN INFECTIOUS AND PARASITIC DISEASES**	
Other gastroenteritis and colitis of infectious and unspecified origin (A09)	1.24 (0.88-1.76)
Erysipelas (A46)	1.59 (1.19-2.12)
Summary estimate	1.47 (1.17-1.84)
**ENDOCRINE, NUTRITIONAL AND METABOLIC DISEASES**	
Other hypothyroidism (E03)	2.20 (1.48-3.26)
Thyrotoxicosis [hyperthyroidism] (E05)	0.91 (0.47-1.74)
Disorders of lipoprotein metabolism and other lipidaemias (E78)	1.42 (1.00-2.01)
Summary estimate	1.52 (1.18-1.94)
**DISEASES OF THE CIRCULATORY SYSTEM**	
Angina pectoris (I20)	1.19 (1.00-1.41)
Acute myocardial infarction (I21)	1.56 (1.18-2.06)
Chronic ischemic heart disease (I25)	1.28 (1.06-1.56)
Atrial fibrillation and flutter (I48)	1.20 (0.97-1.49)
Cerebral infarction (I63)	2.15 (1.61-2.87)
Occlusion and stenosis of precerebral arteries, not resulting in cerebral infarction (I65)	1.55 (0.69-3.45)
Summary estimate	1.40 (1.24-1.59)
**DISEASES OF THE DIGESTIVE SYSTEM**	
Esophagitis (K20)	1.56 (0.84-2.90)
Duodenal ulcer (K26)	1.92 (1.36-2.71)
Gastritis and duodenitis (K29)	1.48 (1.06-2.07)
Other functional intestinal disorders (K59)	0.88 (0.44-1.75)
Other diseases of anus and rectum (K62)	1.30 (0.86-1.98)
Alcoholic liver disease (K70)	0.92 (0.30-2.87)
Summary estimate	1.45 (1.19-1.76)

*The chapter-specific summary estimates were computed considering as exposed all those who had any of the conditions analyzed under the same chapter above.

Table A. 6. Numerical estimates for Fig. 2 in the main text

Exposure	Exposed, No.	Dementia in Exposed, No.	Reference Group, No.	Dementia in Reference Group, No.	Relative Risk (95% CI)		
						Previous meta-analysis	
					Our estimate	Low estimate	High estimate
**Conventional Risk Factors**							
Low education	22,830	622	59,712	316	1.61 (1.38-1.89)	1.59 (1.26-2.09)[4]	1.88 (1.51-2.34)[5]
Hypertension	8047	182	121,577	1110	1.24 (1.06-1.45)	1.18 (1.02-1.35)[6]	1.61 (1.16-2.24)[7]
Obesity	13,767	108	106,877	665	1.16 (0.95-1.43)	0.91 (0.83-1.01)[8]	1.91 (1.40-2.62)[9]
Smoking	26,578	288	104,098	1090	1.34 (1.17-1.53)	1.27 (1.02-1.60)[10]	1.30 (1.18-1.45)[11]
Depression	31,898	425	98,778	953	1.13 (1.00-1.26)	1.08 (1.04-1.12)[12]	1.98 (1.50-2.63)[13]
Physical inactivity	25,189	335	105,487	1043	1.22 (1.08-1.38)	1.01 (0.89-1.13)[14]	1.39 (1.16-1.67)[15]
Social isolation	96,339	990	34,337	388	1.37 (1.22-1.55)	1.21 (1.10-1.33)[16]	1.59 (1.31-1.94)[16]
Diabetes	1954	50	128,722	1328	2.05 (1.54-2.73)	1.43 (1.33-1.53)[17]	1.73 (1.65-1.82)[18]
**Physical Diseases (ICD-10 code)**							
Erysipelas (A46)	4542	135	278,872	3281	1.82 (1.53-2.17)		
Other hypothyroidism (E03)	2702	99	280,712	3317	1.94 (1.59-2.38)		
Acute myocardial infarction (I21)	4072	173	279,342	3243	1.41 (1.20-1.64)		
Chronic ischemic heart disease (I25)	6719	330	276,695	3086	1.32 (1.18-1.49)		
Cerebral infarction (I63)	4201	265	279,213	3151	2.44 (2.14-2.77)		
Duodenal ulcer (K26)	1054	49	282,360	3367	1.88 (1.42-2.49)		
Gastritis and duodenitis (K29)	2280	84	281,134	3332	1.82 (1.46-2.27)		
Osteoporosis without pathological fracture (M81)	617	44	282,797	3372	2.38 (1.75-3.23)		

Abbreviations: CI, confidence interval; ICD-10, International Classification of Diseases, 10th Revision.

Estimates for physical diseases are the same as in the main analysis with full follow-up (reported in Figure 1 in the main text).
Low education/socioeconomic status was compared with high education/socioeconomic status. Hazard ratio (95% CI) for intermediate vs. high education/socioeconomic status was 1.28 (1.10-1.48). Other conventional risk factors were dichotomized.

Marital status was used as a proxy for social isolation when computing our estimate.

Hearing loss was omitted from the conventional risk factors because we had no data on it.

Table A. 7. Association of conventional potentially modifiable dementia risk factors with early and late-onset dementia

	Early-onset dementia (age of onset <65 years)					Late-onset dementia (age of onset ≥65 years)				
Conventional Risk Factors	Exposed, No.	Dementia in Exposed, No.	Reference Group, No.	Dementia in Reference Group, No.	Hazard ratio (95% CI)	Exposed, No.	Dementia in Exposed, No.	Reference Group, No.	Dementia in Reference Group, No.	Hazard ratio (95% CI)
Low education	22,507	86	59,362	97	1.68 (1.22-2.32)	11,680	536	14,969	219	1.60 (1.34-1.92)
Hypertension	8047	32	121,576	260	1.19 (0.82-1.73)	5072	150	36,293	850	1.25 (1.05-1.49)
Obesity	13,767	33	106,876	227	0.97 (0.67-1.40)	4670	75	30,906	438	1.28 (1.00-1.64)
Smoking	26,394	77	103,229	215	1.48 (1.14-1.92)	7429	211	34,988	875	1.30 (1.11-1.51)
Depression	31,653	86	97,970	206	1.20 (0.93-1.55)	11,303	339	31,114	747	1.11 (0.97-1.26)
Physical inactivity	24,925	79	104,698	213	1.38 (1.06-1.78)	9098	256	33,319	830	1.18 (1.02-1.36)
Social isolation	95,561	195	34,062	97	1.59 (1.25-2.04)	32,300	795	10,117	291	1.31 (1.14-1.51)
Diabetes	1900	10	127,723	282	2.11 (1.12-3.97)	890	40	41,527	1046	2.04 (1.48-2.80)

Abbreviations: CI, confidence interval

Low education/socioeconomic status was compared with high education/socioeconomic status. Other conventional risk factors were dichotomized.

Marital status was used as a proxy for social isolation.

Hearing loss was omitted from the conventional risk factors because we had no data on it.

Table A. 8. Association of hospital-treated diseases with subsequent dementia after adjustment for apolipoprotein e genotype

Disease (ICD-10 Code)	Index Disease, No.	Dementia in Index Disease Cases, No.	Reference Group, No.	Dementia in Reference Group, No.	Hazard ratio (95% CI)	
					Model A	Model C
A46 Erysipelas	1	0	5665	163	NA	NA
E03 Other hypothyroidism	214	10	5452	153	1.38 (0.72 to 2.67)	1.31 (0.68 to 2.54)
I21 Acute myocardial infarction	176	7	5490	156	1.47 (0.69 to 3.14)	1.56 (0.73 to 3.34)
I25 Chronic ischemic heart disease	651	22	5015	141	1.20 (0.76 to 1.90)	1.26 (0.79 to 1.99)
I63 Cerebral infarction	93	10	5573	153	5.13 (2.66 to 9.87)	5.18 (2.69 to 10.00)
K26 Duodenal ulcer	50	3	5616	160	2.29 (0.73 to 7.21)	2.16 (0.68 to 6.79)
K29 Gastritis and duodenitis	422	14	5244	149	1.32 (0.76 to 2.28)	1.33 (0.77 to 2.31)
M81 Osteoporosis without pathological fracture	151	8	5515	155	2.02 (0.98 to 4.18)	1.92 (0.93 to 3.98)

Abbreviations: CI, confidence interval; ICD-10, International Classification of Diseases, 10th Revision; NA, not available due to insufficient data.

Diseases are in order of descending hazard ratios of the main analysis (reported in Figure 1 in the main text).

Model A is adjusted for sex, and age is the time scale.

Model C is adjusted for sex and ε4 allele of apolipoprotein E (0 vs. any), and age is the time scale.

Table A. 9. Post hoc power analysis: hazard ratios observable with 90% probability at alpha = 0.05

Disease (ICD-10 code)	Observable hazard ratio
Other gastroenteritis and colitis of infectious and unspecified origin (A09)	1.35
Erysipelas (A46)	1.46
Other hypothyroidism (E03)	1.59
Thyrotoxicosis [hyperthyroidism] (E05)	1.88
Disorders of lipoprotein metabolism and other lipidaemias (E78)	1.40
Disorders of vestibular function (H81)	1.73
Angina pectoris (I20)	1.42
Acute myocardial infarction (I21)	1.48
Chronic ischemic heart disease (I25)	1.38
Atrial fibrillation and flutter (I48)	1.36
Cerebral infarction (I63)	1.47
Occlusion and stenosis of precerebral arteries, not resulting in cerebral infarction (I65)	2.54
Other chronic obstructive pulmonary disease (J44)	1.64
Esophagitis (K20)	2.34
Duodenal ulcer (K26)	1.95
Gastritis and duodenitis (K29)	1.64
Other functional intestinal disorders (K59)	1.72
Other diseases of anus and rectum (K62)	1.67
Alcoholic liver disease (K70)	1.88
Other dermatitis (L30)	2.13
Osteoporosis without pathological fracture (M81)	2.24
Hyperplasia of prostate (N40)	1.53

Power was computed using the method recommended by Scosyrev & Glimm 2019 (equation 19).[19]

Fig. A. 1. Selection of participants in the study

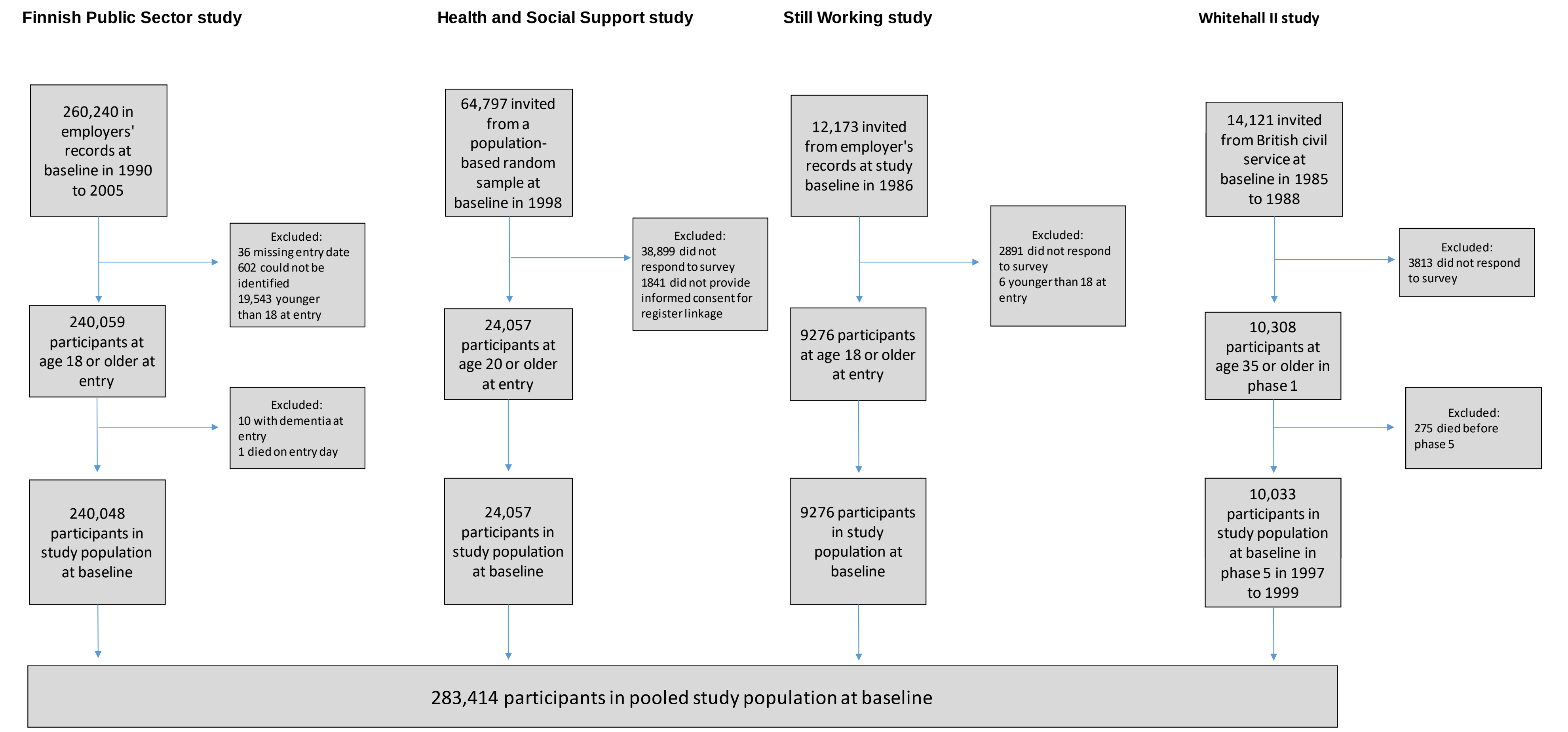

Fig. A. 2. Visualization of hazard ratios over time using exponentiated scaled Schoenfeld residuals

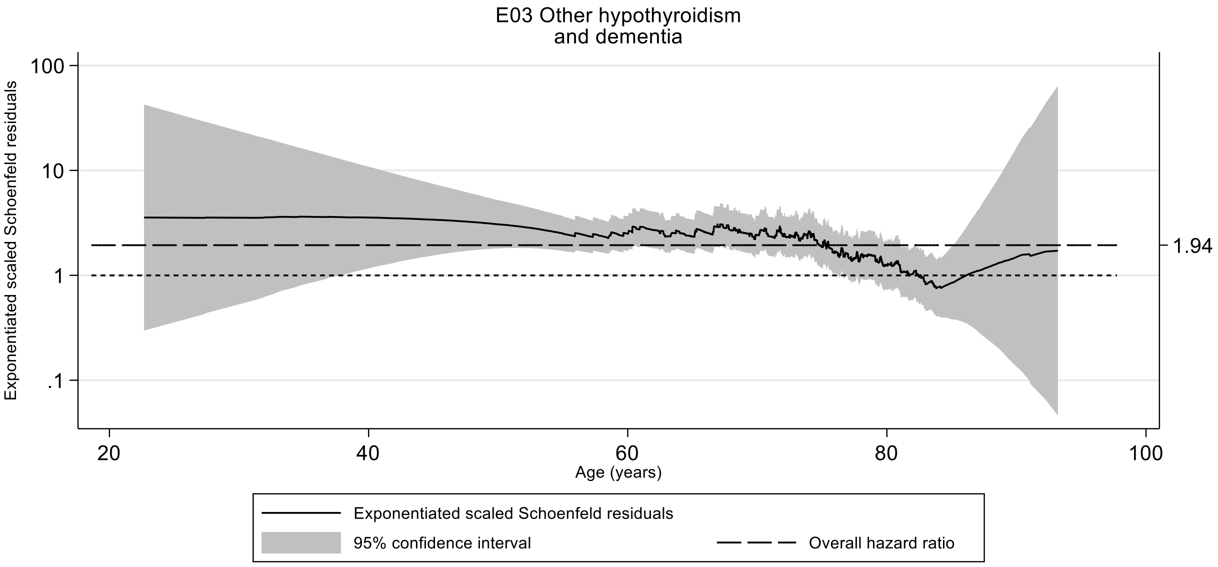

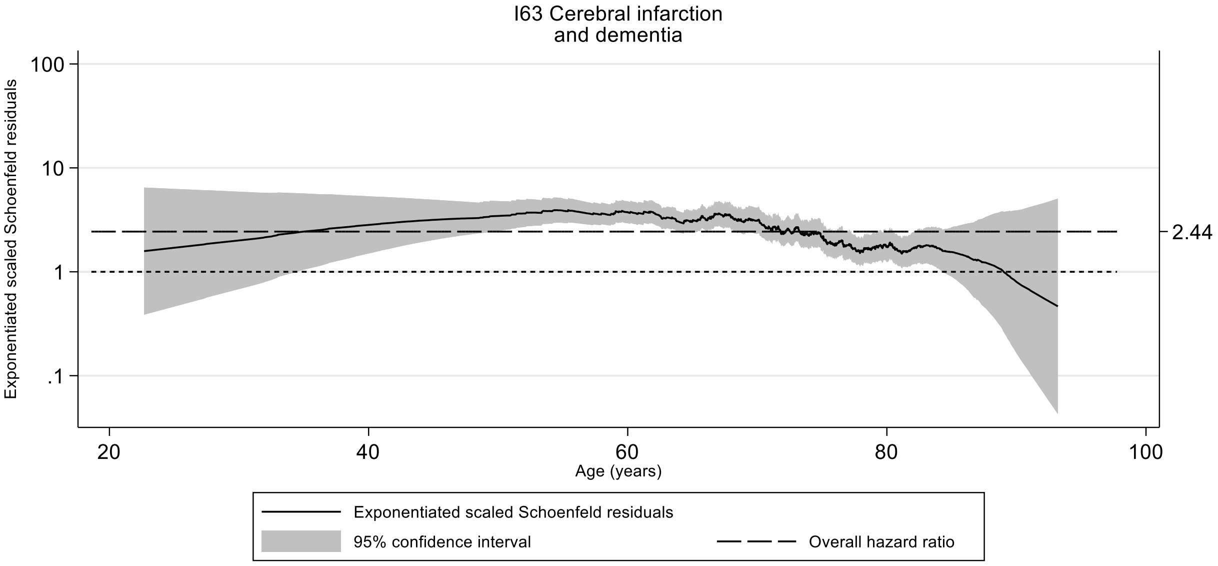

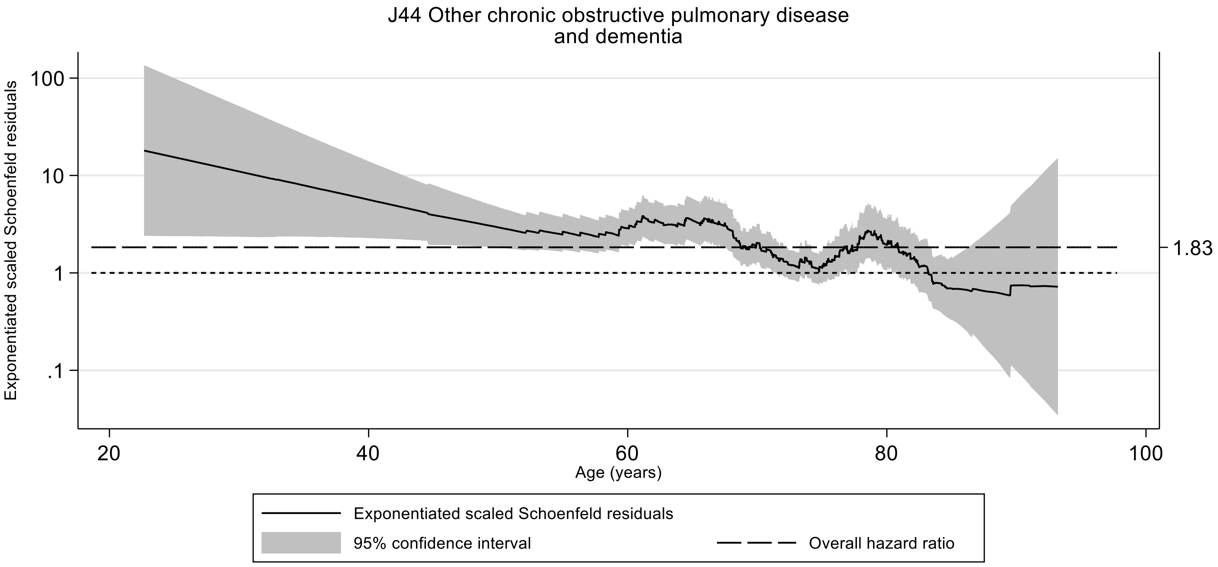

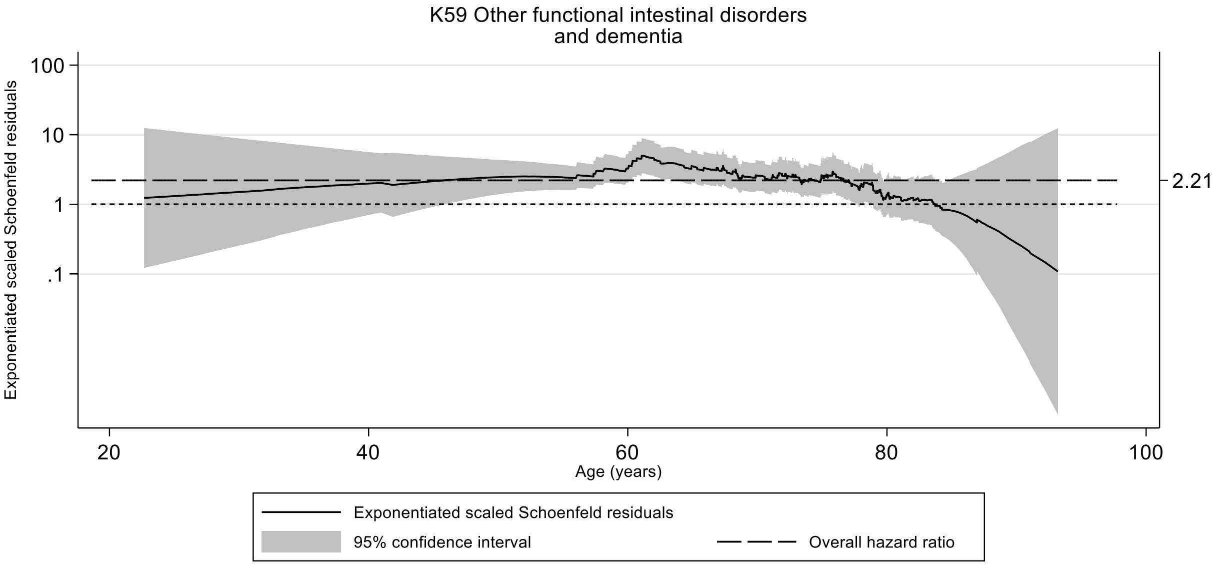

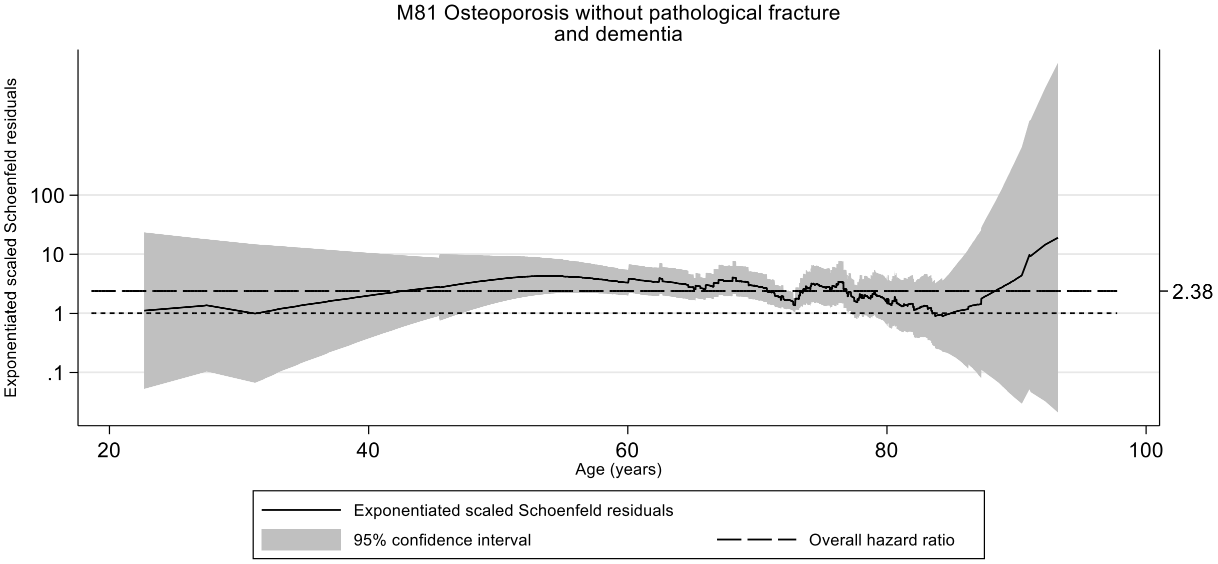

The plots are drawn using symmetric nearest-neighbor smoothing of the scaled Schoenfeld residuals, which can be interpreted as hazard ratios.[20]

Fig. A. 3. Dementia follow-up

Part A. Dementia follow-up in the main analysis

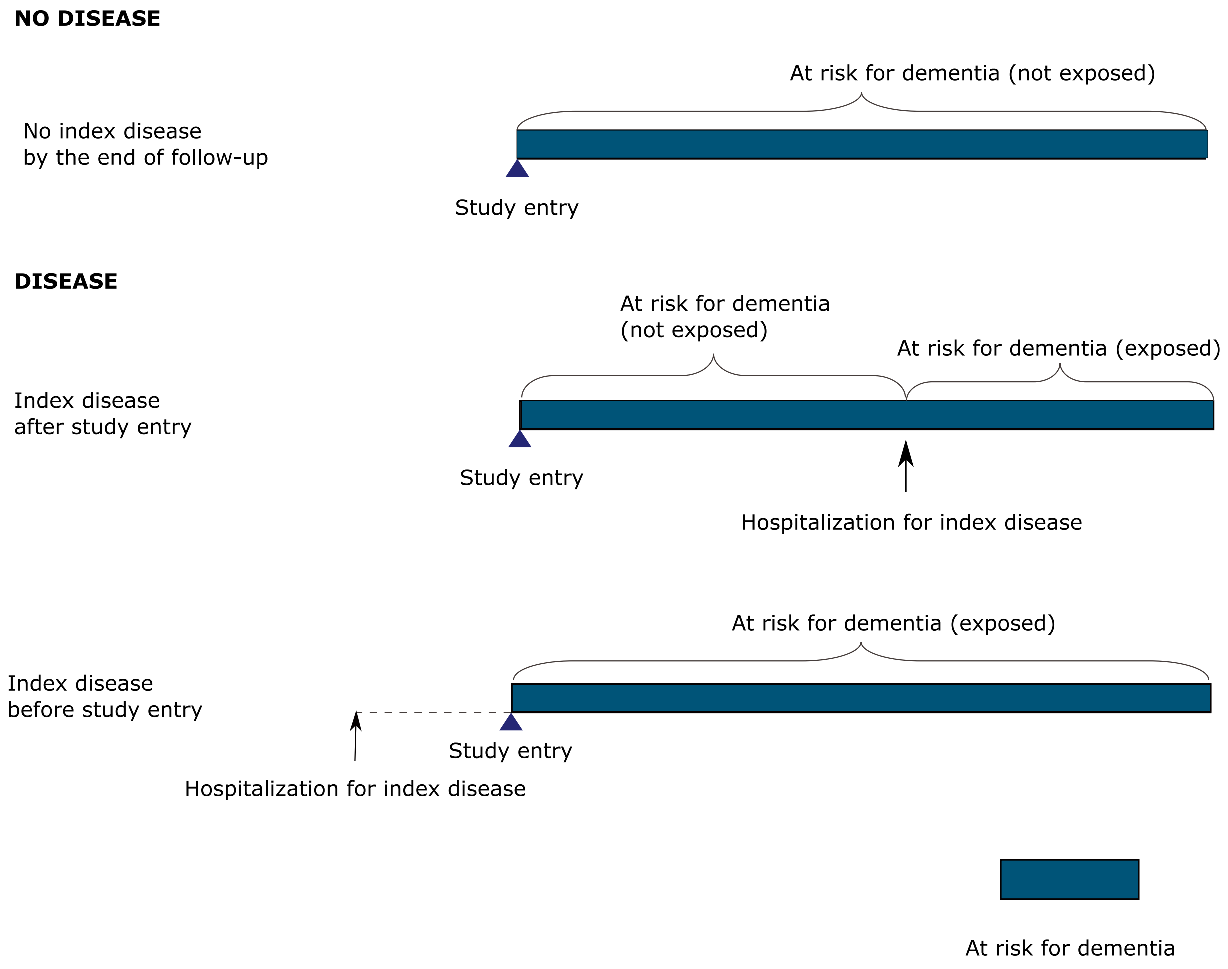

Note: Exposure to index disease is modelled as a time-dependent covariate. Age is the time scale.

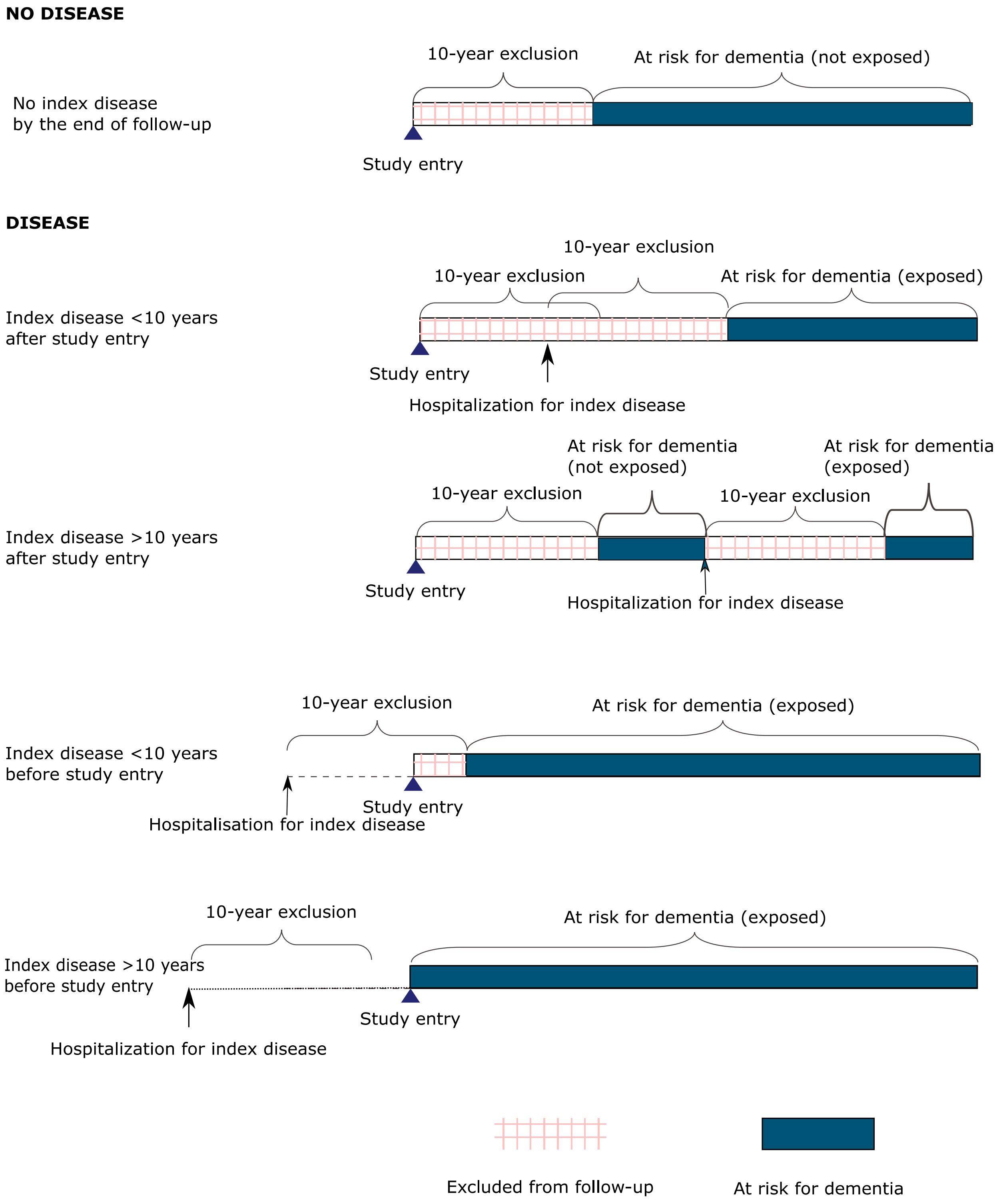

Note: Exposure to index disease is modelled as a time-dependent covariate. Age is the time scale.

Fig. A. 4. Distribution of age at dementia diagnosis

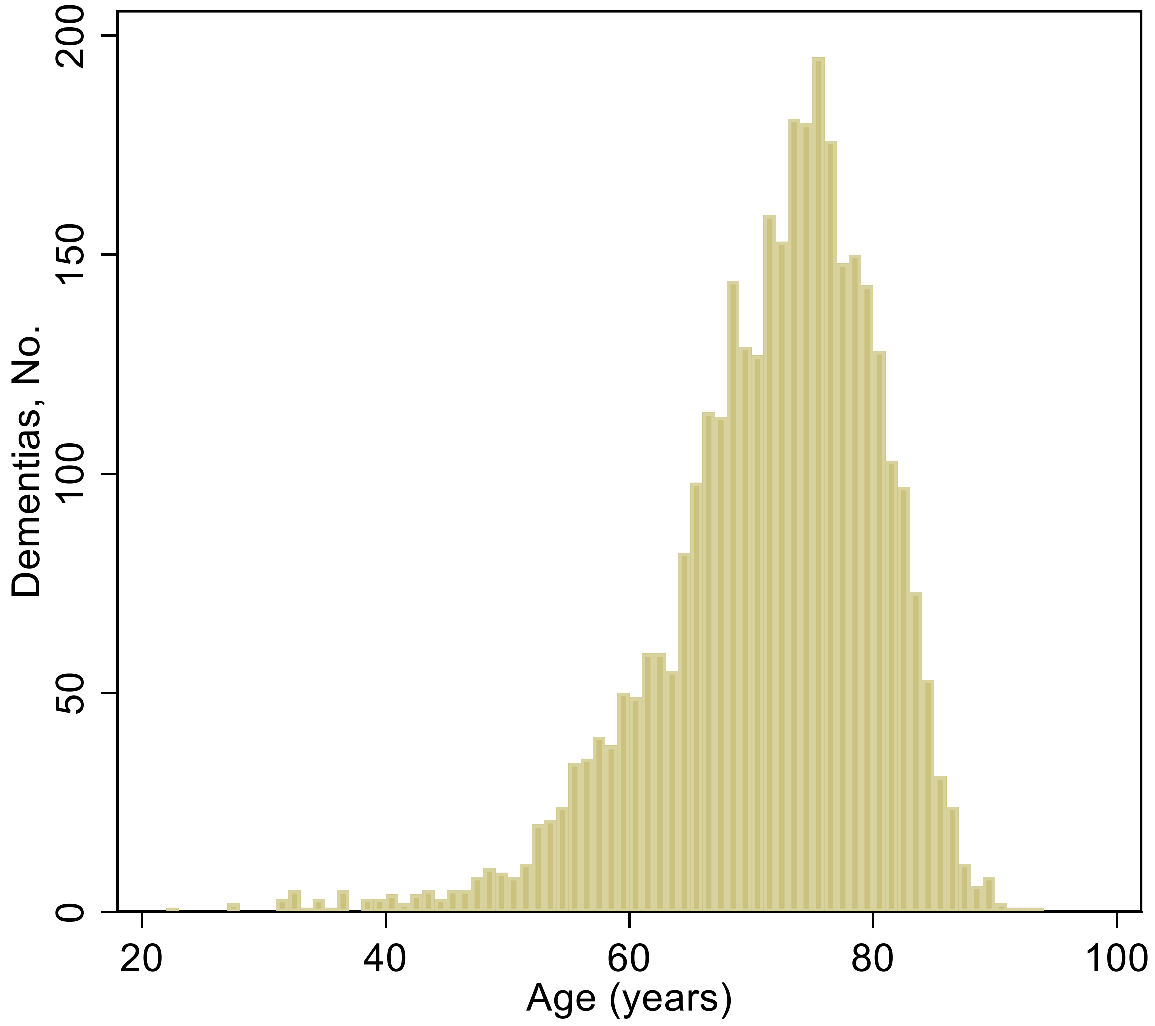

Fig. A. 5. Odds ratios between hospital-treated diseases

**Disease (ICD-10 code)**	Other hypothyroidism (E03)	Acute myocardial infarction (I21)	Chronic ischemic heart disease (I25)	Cerebral infarction (I63)	Duodenal ulcer (K26)	Gastritis and duodenitis (K29)	Osteoporosis without pathological fracture (M81)
Erysipelas (A46)	3.27 (2.71-3.95)	3.64 (3.17-4.17)	4.26 (3.81-4.76)	3.93 (3.45-4.47)	3.38 (2.57-4.43)	2.85 (2.24-3.63)	3.54 (2.23-5.63)
Other hypothyroidism (E03)		2.04 (1.65-2.53)	2.35 (2.03-2.73)	3.00 (2.49-3.62)	1.48 (0.94-2.35)	2.39 (1.94-2.95)	3.32 (2.43-4.53)
Acute myocardial infarction (I21)			58.40 (53.75-63.46)	5.24 (4.68-5.88)	3.47 (2.72-4.43)	2.38 (1.95-2.90)	2.96 (2.14-4.10)
Chronic ischemic heart disease (I25)				5.52 (5.03-6.06)	3.30 (2.71-4.03)	2.56 (2.24-2.92)	2.37 (1.88-2.99)
Cerebral infarction (I63)					3.13 (2.41-4.07)	2.75 (2.25-3.37)	3.71 (2.65-5.20)
Duodenal ulcer (K26)						12.68 (10.17-15.81)	2.51 (1.36-4.62)
Gastritis and duodenitis (K29)							2.48 (1.85-3.33)

Odds ratios	<2	2-4.99	5-9.99	≥10

95% confidence intervals are given in parentheses.

Fig. A. 6. Hazard ratio for association of hospitalization due to disease versus no hospitalization due to disease with incident dementia ascertained using all diagnoses vs. primary diagnosis only

● All diagnoses of physical diseases
▲ Primary diagnoses of physical diseases only

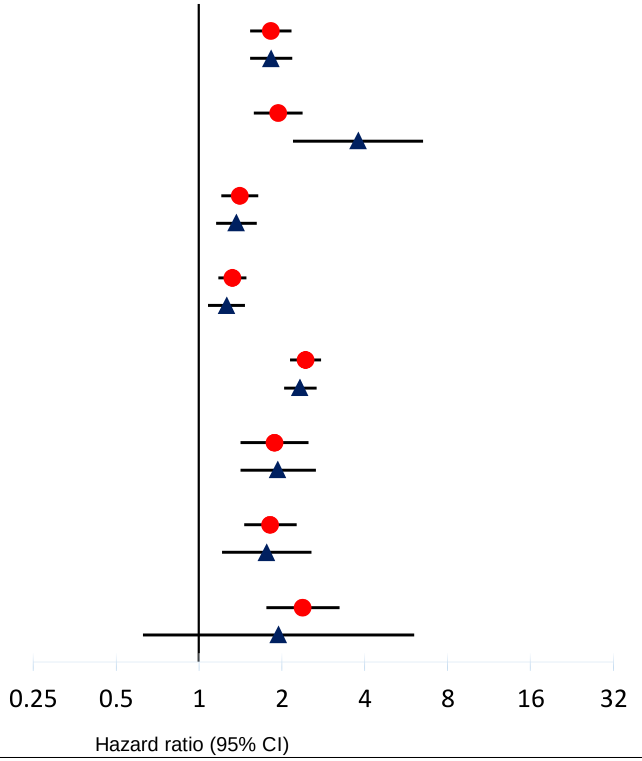

Disease (ICD-10 code)	Hazard ratio (95% CI)	Index disease, No.	Dementia in index disease cases, No.	Reference group, No.	Dementia in reference group, No.
Erysipelas (A46)	1.82 (1.53-2.17)	4542	135	278872	3281
	1.83 (1.53-2.18)	4289	128	269092	2937
Other hypothyroidism (E03)	1.94 (1.59-2.38)	2702	99	280712	3317
	3.78 (2.19-6.53)	196	13	273185	3052
Acute myocardial infarction (I21)	1.41 (1.20-1.64)	4072	173	279342	3243
	1.36 (1.15-1.62)	3397	145	269984	2920
Chronic ischemic heart disease (I25)	1.32 (1.18-1.49)	6719	330	276695	3086
	1.26 (1.08-1.47)	3498	183	269883	2882
Cerebral infarction (I63)	2.44 (2.14-2.77)	4201	265	279213	3151
	2.33 (2.03-2.67)	3642	230	269739	2835
Duodenal ulcer (K26)	1.88 (1.42-2.49)	1054	49	282360	3367
	1.94 (1.42-2.65)	837	40	272544	3025
Gastritis and duodenitis (K29)	1.82 (1.46-2.27)	2280	84	281134	3332
	1.76 (1.21-2.56)	1046	28	272335	3037
Osteoporosis without pathological fracture (M81)	2.38 (1.75-3.23)	617	44	282797	3372
	1.94 (0.62-6.03)	48	3	273333	3062

Analyses restricted to primary diagnoses exclude data from the Whitehall II study, where information on the type of diagnosis (primary vs. secondary) was not available.

The bars represent 95% confidence intervals (CI). Age is the time scale and hazard ratios are adjusted for sex.

Abbreviations: ICD-10, International Classification of Diseases, 10th Revision.

Fig. A. 7. Hazard ratio for association of hospitalization due to disease versus no hospitalization due to disease with incident dementia ascertained using all hospitalizations vs. incident hospitalization only

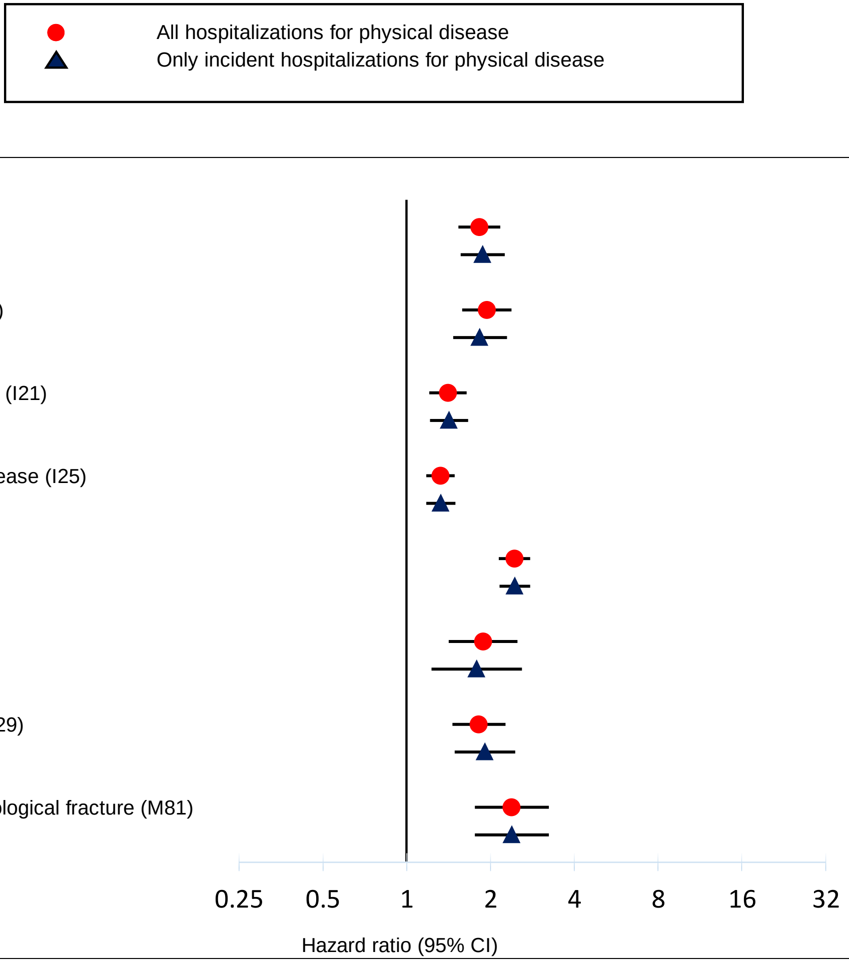

Disease (ICD-10 code)	Hazard ratio (95% CI)	Index disease, No.	Dementia in index disease cases, No.	Reference group, No.	Dementia in reference group, No.
Erysipelas (A46)	1.82 (1.53-2.17)	4542	135	278872	3281
	1.88 (1.56-2.25)	3954	122	278872	3281
Other hypothyroidism (E03)	1.94 (1.59-2.38)	2702	99	280712	3317
	1.83 (1.47-2.28)	2433	85	280712	3317
Acute myocardial infarction (I21)	1.41 (1.20-1.64)	4072	173	279342	3243
	1.42 (1.21-1.66)	4011	173	279342	3243
Chronic ischemic heart disease (I25)	1.32 (1.18-1.49)	6719	330	276695	3086
	1.33 (1.18-1.50)	6447	314	276695	3086
Cerebral infarction (I63)	2.44 (2.14-2.77)	4201	265	279213	3151
	2.44 (2.15-2.78)	4126	263	279213	3151
Duodenal ulcer (K26)	1.88 (1.42-2.49)	1054	49	282360	3367
	1.78 (1.23-2.59)	657	28	282360	3367
Gastritis and duodenitis (K29)	1.82 (1.46-2.27)	2280	84	281134	3332
	1.91 (1.49-2.45)	1614	68	281134	3332
Osteoporosis without pathological fracture (M81)	2.38 (1.75-3.23)	617	44	282797	3372
	2.38 (1.75-3.23)	613	44	282797	3372

The bars represent 95% confidence intervals (CI). Age is the time scale and hazard ratios are adjusted for sex.

Abbreviations: ICD-10, International Classification of Diseases, 10th Revision.

Fig. A. 8. Hazard ratio for association of hospitalization due to disease versus no hospitalization due to disease with incident dementia using data on all dementia cases vs. excluding dementia cases with unknown type of dementia

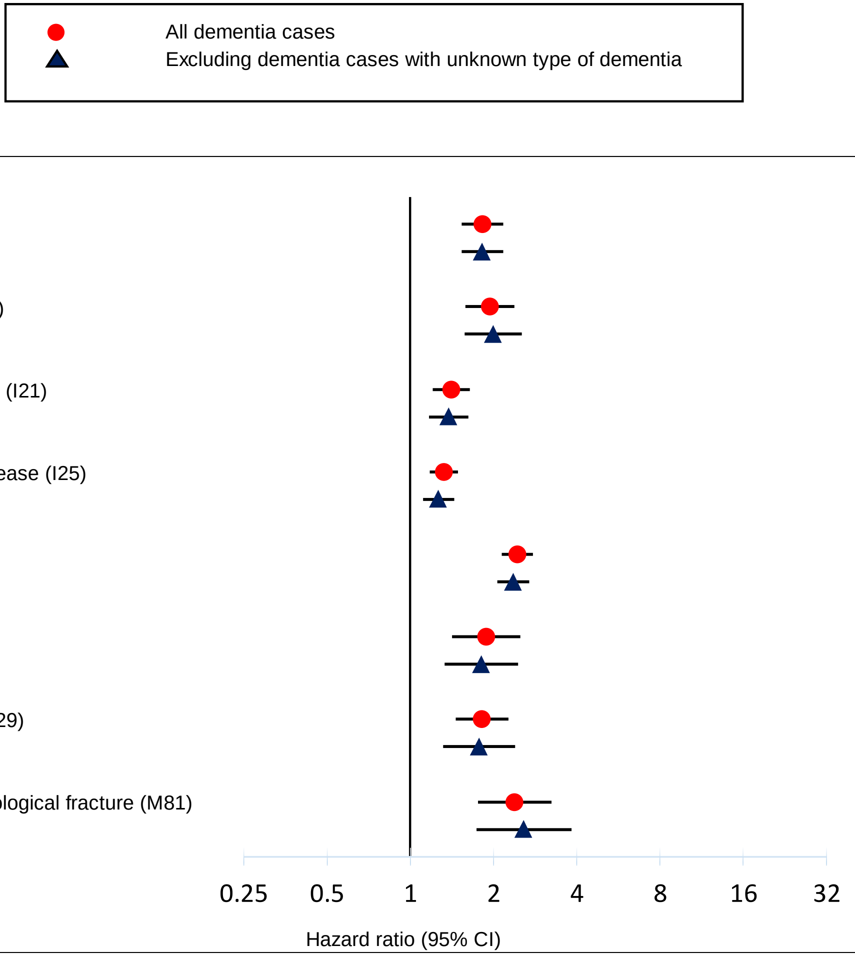

Disease (ICD-10 code)	Hazard ratio (95% CI)	Index disease, No.	Dementia in index disease cases, No.	Reference group, No.	Dementia in reference group, No.
Erysipelas (A46)	1.82 (1.53-2.17)	4542	135	278872	3281
	1.82 (1.53-2.17)	4540	135	268841	2930
Other hypothyroidism (E03)	1.94 (1.59-2.38)	2702	99	280712	3317
	1.99 (1.57-2.53)	2250	71	271131	2994
Acute myocardial infarction (I21)	1.41 (1.20-1.64)	4072	173	279342	3243
	1.37 (1.17-1.62)	3721	155	269660	2910
Chronic ischemic heart disease (I25)	1.32 (1.18-1.49)	6719	330	276695	3086
	1.26 (1.11-1.44)	5440	265	267941	2800
Cerebral infarction (I63)	2.44 (2.14-2.77)	4201	265	279213	3151
	2.35 (2.06-2.69)	3992	246	269389	2819
Duodenal ulcer (K26)	1.88 (1.42-2.49)	1054	49	282360	3367
	1.81 (1.33-2.45)	938	42	272443	3023
Gastritis and duodenitis (K29)	1.82 (1.46-2.27)	2280	84	281134	3332
	1.77 (1.31-2.39)	1446	43	271935	3022
Osteoporosis without pathological fracture (M81)	2.38 (1.75-3.23)	617	44	282797	3372
	2.57 (1.73-3.82)	327	25	273054	3040

Analyses excluding cases with unknown type of dementia exclude data from the Whitehall II study, where information on the type of dementia was not available

The bars represent 95% confidence intervals (CI). Age is the time scale and hazard ratios are adjusted for sex.

Abbreviations: ICD-10, International Classification of Diseases, 10th Revision.

Methods A. 1. Study cohorts and data collection

The Finnish Public Sector study (FPS)

The cohort

The prospective Finnish Public Sector cohort study comprises 260,240 public sector employees from 10 Finnish towns and 6 Finnish hospital districts identified from the employers' registers.[21] 240,048 of them were available for this study and were linked to national registries of hospital discharge information (dates and diagnoses recorded by the National Institute for Health and Welfare) and medication reimbursement entitlements (dates and anatomical therapeutic chemical (ATC) codes recorded by the Finnish Social Insurance Institution) using the personal identification codes unique to each Finnish citizen. We also linked the participants to records on dates and causes of death from Statistics Finland. Informed consent was obtained from all participants except for a subsample, for which it was not required because only deidentified register data were used and the participants were not invited to surveys or clinical examinations. The ethics committee of Helsinki and Uusimaa Hospital District approved the register linkage and data analysis.

Diagnoses of hospital-treated diseases (index diseases)

We retrieved both primary and secondary diagnoses of hospital-treated diseases from inpatient hospital discharge information from 1 January 1980 to 31 December 2016, using the International Classification of Diseases, 10th Revision (ICD-10). The diagnosis codes from the 8th and 9th revisions (ICD-8 and ICD-9) were converted into the corresponding ICD-10 codes according to the national editions of the International Classification of Diseases (eTable 1 in this Supplement).[1–3]

Covariates

Participants' birthdate and sex were retrieved from the Finnish Population Information System. Information on the participants' education was available from Statistics Finland and analyzed in three classes: low (basic education), intermediate (high school or vocational school), and high (university degree or other tertiary degree).

Diabetes mellitus was defined as hospitalization for it (ICD-10 codes E10–E14 and ICD-8 and -9 codes under 250 as primary or secondary diagnosis) or a medication reimbursement entitlement for its treatment by the start of follow-up. Similarly, hypertension was defined as hospitalization for it (ICD-10 codes I10–I15 and ICD-8 and -9 codes 400–40499 as primary or secondary diagnosis) or a medication reimbursement entitlement for its treatment by the start of follow-up. Data for hospitalizations were available from 1 January 1980 to 31 December 2016 and for medication reimbursement entitlements from 1964 to the end-of 2011 (diabetes) or from 1970 to the end-of 2011 (hypertension).

A subsample of the study was invited to respond to mailed questionnaires (N = 114,835), and 92,727 of them responded to at least one questionnaire. Information on smoking status, mental health, physical activity, marital status, and body mass index was retrieved from the earliest available self-report during the follow-up from June 1997 to November 2013 and was available for 89,175 participants. Smoking status was analyzed as a dichotomy whether or not the participants were current smokers. Mental health was measured using the 12-item General Health Questionnaire (GHQ-12) with a cut-off ≥4 used as a proxy for depression.[22] Those reporting "less than 0.5 hours of each (brisk walking, jogging, or running) per week" were defined as physically inactive, and the rest were considered physically active.[23] Body mass index was dichotomized as not obese (≤29.9 kg/m^2) vs. obese (≥30.0 kg/m^2). Marital status (married or cohabiting vs. single, divorced, separated, or widowed) was used as a proxy for social isolation.

Ascertainment of incident dementia

A diagnosis of all-cause dementia comprised ICD-10 codes F00-F03, F05.1, G30, G31.0, G31.1, and G31.8. ICD-8 and -9 codes were converted to the the corresponding ICD-10 codes (Table A. 1 in the Appendix). We compiled dementia diagnoses from hospital discharge information, reimbursed prescription medication purchases, and causes of death. Inpatient discharge information from hospitals and health center wards was available from 1 January 1980 to 31 December 2016, causes of death from 1 January 1980 to 31 December 2011, medication reimbursement entitlements for the treatment of dementia from February 1999 to 31 December 2011, and outpatient hospital visits (including outpatient clinic, emergency room, and day ward visits) from 1 January 2012 to 31 December 2016. The date of incident dementia was defined as the first occurrence of dementia diagnosis, whether primary or secondary, in any of these information sources.

Dementia follow-up

Follow-up for incident dementia started on study entry (the first day of the year in which the participant was first employed between 1990 and 2005) and continued until dementia diagnosis, death, or the end of follow-up (31 December 2016), whichever

came first. However, to avoid immortal time bias,[24] we started the follow-up on the questionnaire date (or the latest questionnaire date if information on covariates was retrieved from more than one questionnaire) in the multivariate analyses (Table 2 in the main text) and in the analyses on conventional dementia risk factors (Figure 3 in the main text).

The Health and Social Support study (HeSSup)

The Health and Social Support study is a prospective cohort study representative of the Finnish population.[25] A random sample from the Finnish Population Information System, stratified by sex and age group (20–24, 30–34, 40–44, and 50–54 years), was invited to respond a mailed questionnaire in 1998 (N = 64,797). This study comprises 24,057 respondents who returned the questionnaire and provided informed consent for register linkage. They were linked to national registries of hospital discharge information (dates and diagnoses recorded by the National Institute for Health and Welfare) and medication reimbursement entitlements (dates and anatomical therapeutic chemical (ATC) codes recorded by the Finnish Social Insurance Institution) using the personal identification codes unique to each Finnish citizen. The participants were also linked to records on dates and causes of death from Statistics Finland. The Turku University Central Hospital Ethics Committee approved the study.

Diagnoses of hospital-treated diseases (index diseases)

We retrieved both primary and secondary diagnoses of hospital-treated diseases from inpatient hospital discharge information available from 1 January 1998 to 31 December 2012 (with occasional information available from 4 August 1997 to 31 December 1997). We used the International Classification of Diseases, 10th Revision (ICD-10). All available data were coded according to ICD-10.

Covariates

Participants' birth year and sex were retrieved from the Finnish Population Information System. Information on education, smoking status, depression, physical inactivity, marital status, and body mass index was retrieved from self-reports. Education was analyzed in three classes: low (basic education), intermediate (apprenticeship, vocational school, or college degree), and high (university or polytechnic degree). Smoking status was analyzed as a dichotomy whether or not the participants were current smokers. Depression was measured using the Beck Depression Inventory (BDI) with a cut-off ≥10.[22] Those reporting "less than 0.5 hours of each (brisk walking, jogging, or running) per week" were defined as physically inactive, and the rest were considered physically active.[23] Body mass index was dichotomized as not obese (≤29.9 kg/m^2) vs. obese (≥30.0 kg/m^2). Marital status (married or cohabiting vs. single, divorced, separated, or widowed) was used as a proxy for social isolation.

Diabetes mellitus was defined as hospitalization for it (ICD-10 codes E10–E14 and ICD-8 and -9 codes under 250 as primary or secondary diagnosis) or a medication reimbursement entitlement for its treatment by the start of follow-up. Similarly, hypertension was defined as hospitalization for it (ICD-10 codes I10–I15 and ICD-8 and -9 codes 400–40499 as primary or secondary diagnosis) or a medication reimbursement entitlement for its treatment by the start of follow-up. Data for hospitalizations were available from 1 January 1998 to 31 December 2012 (with occasional information available from 4 August 1997 to 31 December 1997) and for medication reimbursement entitlements from 1964 to the end-of 2013 (diabetes) or from 1970 to the end-of 2013 (hypertension).

Ascertainment of incident dementia

A diagnosis of all-cause dementia comprised ICD-10 codes F00-F03, F05.1, G30, G31.0, G31.1, and G31.8. There were no ICD-8 or -9 codes in the data.We compiled dementia diagnoses from inpatient hospital discharge information, reimbursed prescription medication purchases, and causes of death. Inpatient discharge information from hospitals and health center wards was available from 1 January 1998 to 31 December 2012 (with occasional information available from 4 August 1997 to 31 December 1997), causes of death from cohort entry (1998) to 31 December 2013, and medication reimbursement entitlements for the treatment of dementia from February 1999 to 31 December 2013. The date of incident dementia was defined as the first occurrence of dementia diagnosis, whether primary or secondary, in any of these information sources.

Dementia follow-up

Follow-up for incident dementia started on study entry (1 January 1998) and continued until dementia diagnosis, death, or end of follow-up (31 December 2012), whichever came first.

The Still Working study (STW)

The cohort

The Still Working study is an ongoing prospective cohort study.[26] In 1986, all employees of Enso Gutzeit (a large forest industry company, which nowadays is part of Stora Enso) in Finland were invited to respond to a mailed questionnaire (N = 12,173). 9276 of them were available for this study and were linked to national registries of hospital discharge information (dates and diagnoses recorded by the National Institute for Health and Welfare) and medication reimbursement entitlements (dates and anatomical therapeutic chemical (ATC) codes recorded by the Finnish Social Insurance Institution) using the personal identification codes unique to each Finnish citizen. Participants were also linked to records on dates and causes of death from Statistics Finland. Informed consent was obtained from all participants. The ethics committee of the Finnish Institute of Occupational Health approved the study.

Diagnoses of hospital-treated diseases (index diseases)

We retrieved both primary and secondary diagnoses of hospital-treated diseases from inpatient hospital discharge information from 1971 to 31 December 2016, using the International Classification of Diseases, 10th Revision (ICD-10). The diagnosis codes from the 8th and 9th revisions (ICD-8 and ICD-9) were converted into the corresponding ICD-10 codes according to the national editions of the International Classification of Diseases (eTable 1 in this Supplement).[1–3]

Covariates

Participants' birth year and sex were retrieved from the Finnish Population Information System. Information on occupational grade, smoking status, depression, physical inactivity, and marital status was retrieved from self-reports. Occupational grade was analyzed in three categories (low, intermediate, high). Smoking status was analyzed as a dichotomy whether or not the participants were current smokers. Depression was defined as a self-assessment of being depressed "now and then" or more often (as opposed to "quite rarely" or "not at all").[22] Those reporting "[S]port activities less than a couple of times per month" were defined as physically inactive, and the rest were considered physically active.[23] Marital status (married vs. not married) was used as a proxy for social isolation. Data on body mass index were not available in this cohort.

Diabetes mellitus was defined as hospitalization for it (ICD-10 codes E10–E14 and ICD-8 and -9 codes under 250 as primary or secondary diagnosis) or a medication reimbursement entitlement for its treatment by the start of follow-up. Similarly, hypertension was defined as hospitalization for it (ICD-10 codes I10–I15 and ICD-8 and -9 codes 400–40499 as primary or secondary diagnosis) or a medication reimbursement entitlement for its treatment by the start of follow-up. Data for hospitalizations were available from 1971 to 31 December 2016 and for medication reimbursement entitlements from 1964 to the end-of 2016 (diabetes) or from 1970 to the end-of 2016 (hypertension).

Ascertainment of incident dementia

A diagnosis of all-cause dementia comprised ICD-10 codes F00-F03, F05.1, G30, G31.0, G31.1, and G31.8. ICD-8 and -9 codes were converted to the the corresponding ICD-10 codes (Table A. 1 in the Appendix). We compiled dementia diagnoses from hospital discharge information, reimbursed prescription medication purchases, and causes of death. Inpatient discharge information from hospitals and health center wards was available from 1971 to 31 December 2016, causes of death from study entry (in 1 March 1986) to 31 December 2016, medication reimbursement entitlements for the treatment of dementia from February 1999 to 31 December 2016, and outpatient hospital visits (including outpatient clinic, emergency room, and day ward visits) from 1 January 2012 to 31 December 2016. The date of incident dementia was defined as the first occurrence of dementia diagnosis, whether primary or secondary, in any of these information sources.

Dementia follow-up

Follow-up for incident dementia started on study entry (1 March 1986) and continued until dementia diagnosis, death, or end of follow-up (31 December 2016), whichever came first.

The Whitehall II study (WHII)

The Whitehall II study is an ongoing prospective cohort study of non-industrial civil servants in London, UK.[27] Of the 14,121 people invited to the first phase between 1985 and 1988, 10,308 participated. 10,033 of them were alive in phase 5 (baseline of the present analysis) and were included in the dementia follow-up. In each study phase, the ethics approval was renewed, and the

participants provided written informed consent. The University College London Hospital Committee on the Ethics of Human Research (reference number 85/0938) provided the most recent approval.

Diagnoses of hospital-treated diseases (index diseases)

Diagnoses of hospital-treated diseases were retrieved from inpatient records of the Hospital Episode Statistics (HES) from 1997 to 31 March 2017, using the International Classification of Diseases, 10th Revision (ICD-10). All available data were coded according to ICD-10. Both primary and secondary diagnoses were retrieved.

Covariates

Participants reported their age, sex, occupational grade, smoking status, mental health, physical activity, and marital status in phase 1 (1985–1988). The time of birth for dementia follow-up was calculated backward from age in phase 1. Occupational grade, which is more strongly associated with dementia than education in this cohort,[28] was analyzed in three categories (low, intermediate, high). Smoking status was analyzed as a dichotomy whether or not the participants were current smokers. Mental health was measured using the 30-item General Health Questionnaire (GHQ-30) with a cut-off ≥5 used as a proxy for depression.[29] Those reporting moderate exercise less than 2.5 hours per week and vigorous exercise less than 1 hour per week were defined as physically inactive, and the rest were considered physically active. Marital status (married or cohabiting vs. single, divorced, separated, or widowed) was used as proxy for social isolation. Body mass index (not obese ≤29.9 kg/m^2 vs. obese ≥30.0 kg/m^2) and hypertension (systolic blood pressure ≥140 mmHg)[30] were measured in phase 1. Diabetes mellitus was defined as known diabetes or fasting glucose ≥7.0 mmol/l in phase 5 (1997–1999). Apolipoprotein E genotype was determined using polymerase chain reaction and analyzed as a dichotomy (0 vs. any ε4 allele).[31]

Ascertainment of incident dementia

Incident dementia was ascertained from inpatient records of the Hospital Episode Statistics using ICD-10 codes G30, G31, F00, F01, and F03. These data were available from 1997 to 31 March 2017. All available data were coded according to ICD-10. The date of incident dementia was defined as the first occurrence of dementia diagnosis, whether primary or secondary, in the Hospital Episode Statistics.

Dementia follow-up

Follow-up for incident dementia started in phase 5 (1997–1999) and continued until dementia diagnosis, death (drawn from the British national mortality register), or end of follow-up (31 March 2017), whichever came first.

Methods A. 2. Proportionality of hazards

We examined the proportional hazards assumption in the Cox models with scaled Schoenfeld residuals. These tests indicated a violation of the proportional hazards assumption for five diseases: other hypothyroidism, cerebral infarction, other chronic obstructive pulmonary disease, other functional intestinal disorders, and osteoporosis without pathological fracture. We assessed these diseases plotting exponentiated scaled Schoenfeld residuals using symmetric nearest neighbor smoothing (eFigure 1 in this Supplement).[20] The exponentiated scaled Schoenfeld residuals can be interpreted as hazard ratios and therefore visualize that the hazard ratios tended to diminish with increasing age.[20] The diminishing hazard ratios are compatible with the observation from cardiovascular epidemiology that the strength of many risk factors tend to decrease at older ages.[32]

Methods A. 3. Stata code for data analysis

**** Variables ****

/*

VARIABLE NAME	VARIABLE DESCRIPTION
id	participant id
rivipvm	row date
ensidementia	all-cause dementia
syntpvm	date of birth
entrypvm	date of study entry
supu	sex
any`idg'	exposed to hospitalization for index disease
anyp`idg'	exposed to hospitalization for index disease (primary diagnoses only)
exitpvm	exit date
*_timedep	time-dependent variables for index diseases
cohort	study cohort
kyselydiabetes	diabetes mellitus
kyselyhypert	hypertension
ensi`idg'pvm	date of hospitalization for index disease
ensip`idg'pvm	date of hospitalization for index disease (primary diagnoses only)
smoker	current smoker
obesity	obese vs not obese
ses	education or socioeconomic status (depending on the cohort)
kyselydepressio	depression
inactive	physical inactivity
married	married or cohabiting

*/

**** Data analysis ****

*** FIGURE 1***

```
local dg `"A09 A46 E03 E05 E78 H81 I20 I21 I25 I48 I63 I65 J44 K20 K26 K29 K59 K62 K70 L30 M81 N40"'

local n_dg: word count `dg'

local dg_name `""Other gastroenteritis and colitis of infectious and unspecified origin" "Erysipelas" "Other hypothyroidism"  "Thyrotoxicosis [hyperthyroidism]" "Disorders of lipoprotein metabolism and other lipidaemias" "Disorders of vestibular function" "Angina pectoris" "Acute myocardial infarction" "Chronic ischaemic heart disease" "Atrial fibrillation and flutter" "Cerebral infarction" "Occlusion and stenosis of precerebral arteries, not resulting in cerebral infarction" "Other chronic obstructive pulmonary disease" "Oesophagitis" "Duodenal ulcer" "Gastritis and duodenitis" "Other functional intestinal disorders" "Other diseases of anus and rectum" "Alcoholic liver disease" "Other dermatitis" "Osteoporosis without pathological fracture" "Hyperplasia of prostate""'

quietly: local irow = 121

forvalues i=1/`n_dg' {

        quietly: local idg : word `i' of `dg'

        quietly: local idg_name : word  `i' of `dg_name'

        display ""

        display "`idg' followed by broad dementia, adjusted for sex and stratified by cohort"

        display ""

        display "Pooled IPD-analysis (with and without 10-year exclusion)"

        display ""

        display "$S_TIME  $S_DATE"

        display ""


        quietly: use "Pooled_trimmed", clear

        quietly: stset exitpvm, id(id) failure(ensidementia) origin(time syntpvm) enter(time entrypvm) scale(365.25)

        quietly: keep id any`idg' supu cohort exitpvm ensidementia syntpvm entrypvm ensi`idg'pvm  _st _d _origin _t _t0

        tab any`idg'

        stsplit `idg'_split, at(0) after(time=ensi`idg'pvm)

        tab `idg'_split

        gen `idg'_timedep =0

        replace `idg'_timedep = 1 if any`idg'==1 & `idg'_split==0

        tab `idg'_timedep
```

```
tab any`idg' if `idg'_split!=-1


quietly: stdescribe
quietly: local failures = `r(N_fail)'
if "`idg'" == "N40" {
stcox `idg'_timedep if supu==1, strata(cohort)
}
else {
stcox `idg'_timedep 2.supu#cohort, strata(cohort)
}
quietly: matrix m = r(table)
tempname hr ll ul
scalar `hr' = m[1,1]
scalar `ll' = m[5,1]
scalar `ul' = m[6,1]
quietly: putexcel set "Pooled results.xlsm", sheet("eFIGURE 5") modify
                quietly: putexcel K`irow'=(`e(N_sub)'), nformat(number)
                quietly: putexcel L`irow'=(`e(N_fail)'), nformat(number)
                quietly: putexcel E`irow'=(`hr'), nformat(number_d2)
                quietly: putexcel F`irow'=(`ll'), nformat(number_d2)
                quietly: putexcel G`irow'=(`ul'), nformat(number_d2)
                quietly: putexcel B`irow'=("`idg'")
                quietly: putexcel C`irow'=("pooled")
                quietly: putexcel D`irow'=("`idg_name'")
tab any`idg', matcell(x), if `idg'_split!=-1
matrix list x
quietly: tab any`idg' ensidementia, matcell(y), if `idg'_split!=-1
matrix list y
quietly: putexcel set "Pooled results.xlsm", sheet("eFIGURE 5") modify
                quietly: putexcel M`irow'=(x[2,1]), nformat(number)
                quietly: putexcel N`irow'=(y[2,1]), nformat(number)
local ++irow
```

```
gen ensi`idg'pvm10v = ensi`idg'pvm + round(10*365.25) if ensi`idg'pvm<entrypvm
replace ensi`idg'pvm10v = entrypvm if ensi`idg'pvm10v<entrypvm
replace _t0 = (ensi`idg'pvm10v-syntpvm)/365.25 if ensi`idg'pvm10v!=.
replace _t0 = _t0 + 10 if ensi`idg'pvm10v==.
drop if _t0>=_t
quietly: stdescribe
quietly: local failures = `r(N_fail)'
if "`idg'" == "N40" {
stcox `idg'_timedep if supu==1, strata(cohort)
}
else {
stcox `idg'_timedep 2.supu#cohort, strata(cohort)
}
quietly: matrix m = r(table)
scalar `hr' = m[1,1]
scalar `ll' = m[5,1]
scalar `ul' = m[6,1]
quietly: putexcel set "Pooled results.xlsm", sheet("eFIGURE 5") modify
        quietly: putexcel K`irow'=(`e(N_sub)'), nformat(number)
        quietly: putexcel L`irow'=(`e(N_fail)'), nformat(number)
        quietly: putexcel E`irow'=(`hr'), nformat(number_d2)
        quietly: putexcel F`irow'=(`ll'), nformat(number_d2)
        quietly: putexcel G`irow'=(`ul'), nformat(number_d2)
        quietly: putexcel B`irow'=("   `idg'_10y_exclusion")
        quietly: putexcel C`irow'=("pooled")
        quietly: putexcel D`irow'=("   Dementia occurring more than 10 years
after the hospitalisation")
tab any`idg', matcell(x), if `idg'_split!=-1
matrix list x
quietly: tab any`idg' ensidementia, matcell(y), if `idg'_split!=-1
matrix list y
quietly: putexcel set "Pooled results.xlsm", sheet("eFIGURE 5") modify
        quietly: putexcel M`irow'=(x[2,1]), nformat(number)
```

```
quietly: putexcel N`irow'=(y[2,1]), nformat(number)
local ++irow
local ++irow
}
```

********Figure 2 and Table A. 6*********

********HRs for conventional risk factors***************************

```
use "Pooled_trimmed", clear

drop if cohort==3
keep if ses!=. & obesity!=. & smoker!=. & kyselydepressio !=. & inactive!=. & married!=. & kyselydiabetes!=. & kyselyhypert!=.
stset exitpvm, id(id) failure(ensidementia) origin(time syntpvm) enter(time viimentrypvm) scale(365.25)
keep if _st==1
count
count if ensidementia==1

*Low education
gen low_vs_highses = ses
recode low_vs_highses 2=. 3=0
tab low_vs_highses _d, matcell(n)
stcox b3.ses 2.supu#cohort, strata(cohort)
matrix a = r(table)
local irow = 6
quietly: putexcel set "Pooled results.xlsm", sheet(”FIGURE 2”) modify
quietly: putexcel E`irow'=n[2,1]+n[2,2], nformat(number_d1)
quietly: putexcel F`irow'=n[2,2], nformat(number_d1)
quietly: putexcel G`irow'=n[1,1]+n[1,2], nformat(number_d1)
quietly: putexcel H`irow'=(n[1,2]), nformat(number_d1)
quietly: putexcel Y`irow'=(a[1,1]), nformat(number_d1)
```

```
                    quietly: putexcel Z`irow'=(a[5,1]), nformat(number_d1)
                    quietly: putexcel AA`irow'=(a[6,1]), nformat(number_d1)



*Midlife hypertension (ne pudotetaan pois, joiden seuranta alkaa vasta 65-vuotiaana tai myöhemmin)
drop if _t0>=65

tab kyselyhypert _d, matcell(n)
stcox kyselyhypert 2.supu#cohort, strata(cohort)
matrix a = r(table)
local irow = 7
quietly: putexcel set "Pooled results.xlsm", sheet(”FIGURE 2”) modify
                    quietly: putexcel E`irow'=n[2,1]+n[2,2], nformat(number_d1)
                    quietly: putexcel F`irow'=n[2,2], nformat(number_d1)
                    quietly: putexcel G`irow'=n[1,1]+n[1,2], nformat(number_d1)
                    quietly: putexcel H`irow'=(n[1,2]), nformat(number_d1)
                    quietly: putexcel Y`irow'=(a[1,1]), nformat(number_d1)
                    quietly: putexcel Z`irow'=(a[5,1]), nformat(number_d1)
                    quietly: putexcel AA`irow'=(a[6,1]), nformat(number_d1)

*Midlife obesity (ne pudotetaan pois, joiden seuranta alkaa vasta 65-vuotiaana tai myöhemmin)
tab obesity _d, matcell(n), if cohort!=3
stcox obesity 2.supu#cohort, strata(cohort), if cohort!=3
matrix a = r(table)
local irow = 8
quietly: putexcel set "Pooled results.xlsm", sheet(”FIGURE 2”) modify
                    quietly: putexcel E`irow'=n[2,1]+n[2,2], nformat(number_d1)
                    quietly: putexcel F`irow'=n[2,2], nformat(number_d1)
                    quietly: putexcel G`irow'=n[1,1]+n[1,2], nformat(number_d1)
                    quietly: putexcel H`irow'=(n[1,2]), nformat(number_d1)
                    quietly: putexcel Y`irow'=(a[1,1]), nformat(number_d1)
                    quietly: putexcel Z`irow'=(a[5,1]), nformat(number_d1)
                    quietly: putexcel AA`irow'=(a[6,1]), nformat(number_d1)
```

```
*Smoking
use "Pooled_trimmed", clear
replace obesity = 0 if cohort==3
keep if ses!=. & obesity!=. & smoker!=. & kyselydepressio !=. & inactive!=. & married!=. & kyselydiabetes!=. & kyselyhypert!=.
stset exitpvm, id(id) failure(ensidementia) origin(time syntpvm) enter(time viimentrypvm) scale(365.25)
keep if _st==1

tab smoker _d, matcell(n)
stcox smoker 2.supu#cohort, strata(cohort)
matrix a = r(table)
local irow = 9
quietly: putexcel set "Pooled results.xlsm", sheet(”FIGURE 2”) modify
        quietly: putexcel E`irow'=n[2,1]+n[2,2], nformat(number_d1)
        quietly: putexcel F`irow'=n[2,2], nformat(number_d1)
        quietly: putexcel G`irow'=n[1,1]+n[1,2], nformat(number_d1)
        quietly: putexcel H`irow'=(n[1,2]), nformat(number_d1)
        quietly: putexcel Y`irow'=(a[1,1]), nformat(number_d1)
        quietly: putexcel Z`irow'=(a[5,1]), nformat(number_d1)
        quietly: putexcel AA`irow'=(a[6,1]), nformat(number_d1)

*Depression

tab kyselydepressio _d, matcell(n)
stcox kyselydepressio 2.supu#cohort, strata(cohort)
matrix a = r(table)
local irow = 10
quietly: putexcel set "Pooled results.xlsm", sheet(”FIGURE 2”) modify
        quietly: putexcel E`irow'=n[2,1]+n[2,2], nformat(number_d1)
        quietly: putexcel F`irow'=n[2,2], nformat(number_d1)
        quietly: putexcel G`irow'=n[1,1]+n[1,2], nformat(number_d1)
```

```
quietly: putexcel H`irow'=(n[1,2]), nformat(number_d1)
quietly: putexcel Y`irow'=(a[1,1]), nformat(number_d1)
quietly: putexcel Z`irow'=(a[5,1]), nformat(number_d1)
quietly: putexcel AA`irow'=(a[6,1]), nformat(number_d1)

*Physical inactivity

tab inactive _d, matcell(n)
stcox inactive 2.supu#cohort, strata(cohort)
matrix a = r(table)
local irow = 11
quietly: putexcel set "Pooled results.xlsm", sheet(”FIGURE 2”) modify
quietly: putexcel E`irow'=n[2,1]+n[2,2], nformat(number_d1)
quietly: putexcel F`irow'=n[2,2], nformat(number_d1)
quietly: putexcel G`irow'=n[1,1]+n[1,2], nformat(number_d1)
quietly: putexcel H`irow'=(n[1,2]), nformat(number_d1)
quietly: putexcel Y`irow'=(a[1,1]), nformat(number_d1)
quietly: putexcel Z`irow'=(a[5,1]), nformat(number_d1)
quietly: putexcel AA`irow'=(a[6,1]), nformat(number_d1)

*Social isolation by proxy

tab married _d, matcell(n)
stcox 0.married 2.supu#cohort, strata(cohort)
matrix a = r(table)
local irow = 12
quietly: putexcel set "Pooled results.xlsm", sheet(”FIGURE 2”) modify
quietly: putexcel E`irow'=n[2,1]+n[2,2], nformat(number_d1)
quietly: putexcel F`irow'=n[2,2], nformat(number_d1)
quietly: putexcel G`irow'=n[1,1]+n[1,2], nformat(number_d1)
quietly: putexcel H`irow'=(n[1,2]), nformat(number_d1)
quietly: putexcel Y`irow'=(a[1,1]), nformat(number_d1)
quietly: putexcel Z`irow'=(a[5,1]), nformat(number_d1)
```

```
				quietly: putexcel AA`irow'=(a[6,1]), nformat(number_d1)

		*Diabetes

		tab kyselydiabetes _d, matcell(n)
		stcox kyselydiabetes 2.supu#cohort, strata(cohort)
		matrix a = r(table)
		local irow = 13
		quietly: putexcel set "Pooled results.xlsm", sheet(”FIGURE 2”) modify
				quietly: putexcel E`irow'=n[2,1]+n[2,2], nformat(number_d1)
				quietly: putexcel F`irow'=n[2,2], nformat(number_d1)
				quietly: putexcel G`irow'=n[1,1]+n[1,2], nformat(number_d1)
				quietly: putexcel H`irow'=(n[1,2]), nformat(number_d1)
				quietly: putexcel Y`irow'=(a[1,1]), nformat(number_d1)
				quietly: putexcel Z`irow'=(a[5,1]), nformat(number_d1)
				quietly: putexcel AA`irow'=(a[6,1]), nformat(number_d1)

*** FIGURE 3 ***

local dg `"I63 M81 E03 K26 A46 K29 I21 I25"'
local dg_name `""Cerebral infarction" "Osteoporosis without pathological fracture" "Other hypothyroidism" "Duodenal ulcer" "Erysipelas" "Gastritis and duodenitis" "Acute myocardial infarction" "Chronic ischaemic heart disease""'
local n_dg: word count `dg'
quietly: local irow = 12
		forvalues i=1/`n_dg' {
			quietly: local idg : word `i' of `dg'
			quietly: local idg_name : word  `i' of `dg_name'
			display ""
			display "`idg' followed by broad dementia, adjusted for sex and stratified by cohort, early and late-onset dementia"
			display ""
			display "$S_TIME  $S_DATE"
			display ""
```

```
quietly: use "Pooled_trimmed", clear
quietly: stset exitpvm, id(id) failure(ensidementia) origin(time syntpvm) enter(time entrypvm) scale(365.25)
quietly: keep id any`idg' supu cohort exitpvm ensidementia syntpvm entrypvm ensi`idg'pvm  _st _d _origin _t _t0
tab any`idg'
stsplit `idg'_split, at(0) after(time=ensi`idg'pvm)
tab `idg'_split
gen `idg'_timedep =0
replace `idg'_timedep = 1 if any`idg'==1 & `idg'_split==0
tab `idg'_timedep
tab any`idg' if `idg'_split!=-1

stsplit age65, at(64.999)
recode age65 64.999=1

tab age65
stdescribe if age65==0
quietly: local failures = `r(N_fail)'
quietly: local N = `r(N_sub)'
display `N'
display `failures'
stdescribe if age65==0 & `idg'_timedep==1
quietly: local failures_exposed = `r(N_fail)'
quietly: local N_exposed = `r(N_sub)'
display `N_exposed'
display `failures_exposed'
if "`idg'" == "N40" {
stcox `idg'_timedep 1.`idg'_timedep#1.age65 if supu==1, strata(cohort)
}
else {
stcox `idg'_timedep 1.`idg'_timedep#1.age65 2.supu#cohort, strata(cohort)
}
```

```
quietly: matrix m = r(table)

matrix list m

tempname hr ll ul pdif

scalar `hr' = m[1,1]

scalar `ll' = m[5,1]

scalar `ul' = m[6,1]

scalar `pdif' = m[4,2]

quietly: putexcel set "Pooled results.xlsm", sheet(”FIGURE 3”) modify

			quietly: putexcel E`irow'=(`hr'), nformat(number_d2)

			quietly: putexcel F`irow'=(`ll'), nformat(number_d2)

			quietly: putexcel G`irow'=(`ul'), nformat(number_d2)

			quietly: putexcel K`irow'=(`N'), nformat(number_d2)

			quietly: putexcel L`irow'=(`failures'), nformat(number_d2)

			quietly: putexcel M`irow'=(`N_exposed'), nformat(number_d2)

			quietly: putexcel N`irow'=(`failures_exposed'), nformat(number_d2)

			quietly: putexcel AC`irow'=(`pdif'), nformat(number_d2)

			quietly: putexcel B`irow'=("`idg'")

			quietly: putexcel C`irow'=("pooled")

			quietly: putexcel D`irow'=("`idg_name'")

local ++irow


stdescribe if age65==1

quietly: local failures = `r(N_fail)'

quietly: local N = `r(N_sub)'

display `N'

display `failures'

stdescribe if age65==1 & `idg'_timedep==1

quietly: local failures_exposed = `r(N_fail)'

quietly: local N_exposed = `r(N_sub)'

display `N_exposed'

display `failures_exposed'


if "`idg'" == "N40" {
```

```
stcox `idg'_timedep 1.`idg'_timedep#0.age65 if supu==1, strata(cohort)
}
else {
stcox `idg'_timedep 1.`idg'_timedep#0.age65 2.supu#cohort, strata(cohort)
}
quietly: matrix m = r(table)
matrix list m
tempname hr ll ul
scalar `hr' = m[1,1]
scalar `ll' = m[5,1]
scalar `ul' = m[6,1]
quietly: putexcel set "Pooled results.xlsm", sheet(”FIGURE 3”) modify
        quietly: putexcel E`irow'=(`hr'), nformat(number_d2)
        quietly: putexcel F`irow'=(`ll'), nformat(number_d2)
        quietly: putexcel G`irow'=(`ul'), nformat(number_d2)
        quietly: putexcel K`irow'=(`N'), nformat(number_d2)
        quietly: putexcel L`irow'=(`failures'), nformat(number_d2)
        quietly: putexcel M`irow'=(`N_exposed'), nformat(number_d2)
        quietly: putexcel N`irow'=(`failures_exposed'), nformat(number_d2)
        quietly: putexcel B`irow'=("`idg'")
        quietly: putexcel C`irow'=("pooled")
        quietly: putexcel D`irow'=("`idg_name'")
local ++irow
local ++irow
}
```

```
*** TABLE 2 ***

local dg `"A46 E03 I21 I25 I63 K26 K29 M81"'
local n_dg: word count `dg'
local dg_name `""Erysipelas" "Other hypothyroidism" "Acute myocardial infarction" "Chronic ischaemic heart disease" "Cerebral infarction" "Duodenal ulcer" "Gastritis and duodenitis" "Osteoporosis without pathological fracture""'
        quietly: local irow = 11
```

```
forvalues i=1/`n_dg' {
		quietly: local idg : word `i' of `dg'
		quietly: local idg_name : word  `i' of `dg_name'
		display ""
		display "`idg' followed by non-vascular dementia"
		display ""
		display "Pooled IPD-analysis adjusted for sex and stratified for cohort"
		display ""
		display "$S_TIME  $S_DATE"
		display ""


		quietly: use "Pooled_trimmattu", clear
		tab dementiatyyppi
		recode ensidementia 1=. if dementiatyyppi==2
		drop if cohort ==4 // drop observations from WHII where information on the type of dementia was not available
		quietly: stset exitpvm, id(id) failure(ensidementia) origin(time syntpvm) enter(time entrypvm) scale(365.25)
		quietly: keep id any`idg' supu cohort exitpvm ensidementia syntpvm entrypvm ensi`idg'pvm  _st _d _origin _t _t0 failure
		tab any`idg'
		stsplit `idg'_split, at(0) after(time=ensi`idg'pvm)
		tab `idg'_split
		gen `idg'_timedep =0
		replace `idg'_timedep = 1 if any`idg'==1 & `idg'_split==0
		tab `idg'_timedep
		tab any`idg' if `idg'_split!=-1


		quietly: stdescribe
		quietly: local failures = `r(N_fail)'
		stcox `idg'_timedep 2.supu#cohort , strata(cohort)
		quietly: matrix m = r(table)
		tempname hr ll ul p
		scalar `hr' = m[1,1]
```

```
scalar `ll' = m[5,1]
scalar `ul' = m[6,1]
scalar `p'  = m[4,1]
quietly: putexcel set "Pooled results.xlsm", sheet("nonvascular") modify
        quietly: putexcel K`irow'=(`e(N_sub)'), nformat(number)
        quietly: putexcel L`irow'=(`e(N_fail)'), nformat(number)
        quietly: putexcel E`irow'=(`hr'), nformat(number_d2)
        quietly: putexcel F`irow'=(`ll'), nformat(number_d2)
        quietly: putexcel G`irow'=(`ul'), nformat(number_d2)
        quietly: putexcel AF`irow'=(`p'), nformat(number_d3)
        quietly: putexcel B`irow'=("`idg'")
        quietly: putexcel C`irow'=("pooled")
        quietly: putexcel D`irow'=("`idg_name'")
tab any`idg', matcell(x), if `idg'_split!=-1
matrix list x
quietly: tab any`idg' ensidementia, matcell(y), if `idg'_split!=-1
matrix list y
quietly: putexcel set "Pooled results.xlsm", sheet("nonvascular") modify
        quietly: putexcel M`irow'=(x[2,1]), nformat(number)
        quietly: putexcel N`irow'=(y[2,1]), nformat(number)
local ++irow
}
```

```
local dg `"A46 E03 I21 I25 I63 K26 K29 M81"'
local n_dg: word count `dg'
local dg_name `""Erysipelas" "Other hypothyroidism" "Acute myocardial infarction" "Chronic ischaemic heart disease" "Cerebral infarction" "Duodenal ulcer" "Gastritis and duodenitis" "Osteoporosis without pathological fracture""'

    quietly: stset exitpvm, id(id) failure(ensidementia) origin(time syntpvm) enter(time entrypvm) scale(365.25)
    quietly: keep id any* supu cohort exitpvm ensidementia syntpvm entrypvm ensi*pvm  _st _d _origin _t _t0
```

```
forvalues i=1/`n_dg' {
        quietly: local idg : word `i' of `dg'
        quietly: local idg_name : word  `i' of `dg_name'


        tab any`idg'
        stsplit `idg'_split, at(0) after(time=ensi`idg'pvm)
        tab `idg'_split
        gen `idg'_timedep =0
        replace `idg'_timedep = 1 if any`idg'==1 & `idg'_split==0
        tab `idg'_timedep
        tab any`idg' if `idg'_split!=-1
        }


        local irow 11
forvalues i=1/`n_dg' {
        quietly: local idg : word `i' of `dg'
        quietly: local idg_name : word  `i' of `dg_name'
if "`idg'" == "I21" {
stcox `idg'_timedep A46_timedep E03_timedep I63_timedep K26_timedep K29_timedep
M81_timedep 2.supu#cohort, strata(cohort)
}
else if "`idg'" == "I25" {
stcox `idg'_timedep A46_timedep E03_timedep I63_timedep K26_timedep K29_timedep
M81_timedep 2.supu#cohort, strata(cohort)
}
else {
stcox `idg'_timedep *_timedep 2.supu#cohort, strata(cohort)
        }
        quietly: matrix m = r(table)
        tempname hr ll ul p
        scalar `hr' = m[1,1]
        scalar `ll' = m[5,1]
        scalar `ul' = m[6,1]
        scalar `p'  = m[4,1]
```

```
quietly: putexcel set "Pooled results.xlsm", sheet("adj_other_dis") modify
        quietly: putexcel K`irow'=(`e(N_sub)'), nformat(number)
        quietly: putexcel L`irow'=(`e(N_fail)'), nformat(number)
        quietly: putexcel E`irow'=(`hr'), nformat(number_d2)
        quietly: putexcel F`irow'=(`ll'), nformat(number_d2)
        quietly: putexcel G`irow'=(`ul'), nformat(number_d2)
        quietly: putexcel AF`irow'=(`p'), nformat(number_d3)
        quietly: putexcel B`irow'=("`idg'")
        quietly: putexcel C`irow'=("pooled")
        quietly: putexcel D`irow'=("`idg_name'")
tab any`idg', matcell(x), if `idg'_split!=-1
matrix list x
quietly: tab any`idg' ensidementia, matcell(y), if `idg'_split!=-1
matrix list y
quietly: putexcel set "Pooled results.xlsm", sheet("adj_other_dis") modify
        quietly: putexcel M`irow'=(x[2,1]), nformat(number)
        quietly: putexcel N`irow'=(y[2,1]), nformat(number)
local ++irow
}
```

```
local dg `"A46 E03 I21 I25 I63 K26 K29 M81"'
local n_dg: word count `dg'
local dg_name `""Erysipelas" "Other hypothyroidism" "Acute myocardial infarction" "Chronic ischaemic heart disease" "Cerebral infarction" "Duodenal ulcer" "Gastritis and duodenitis" "Osteoporosis without pathological fracture""'
    quietly: local irow = 11
    forvalues i=1/`n_dg' {
        quietly: local idg : word `i' of `dg'
        quietly: local idg_name : word  `i' of `dg_name'
        display ""
        display "`idg' followed by broad dementia, adjusted for sex and cohort"
        display ""
        display "Pooled IPD-analysis using Fine and Gray model"
        display ""
```

```
display "$S_TIME  $S_DATE"
display ""

quietly: use "Pooled_trimmattu", clear
quietly: stset exitpvm, id(id) failure(ensidementia) origin(time syntpvm) enter(time entrypvm) scale(365.25)
quietly: keep id any`idg' supu cohort exitpvm ensidementia syntpvm entrypvm ensi`idg'pvm  _st _d _origin _t _t0 failure
tab any`idg'
stsplit `idg'_split, at(0) after(time=ensi`idg'pvm)
tab `idg'_split
gen `idg'_timedep =0
replace `idg'_timedep = 1 if any`idg'==1 & `idg'_split==0
tab `idg'_timedep
tab any`idg' if `idg'_split!=-1

quietly: stdescribe
quietly: local failures = `r(N_fail)'
stcrreg `idg'_timedep 2.supu#cohort i.cohort, cl(cohort) compete(failure==2)
quietly: matrix m = r(table)
tempname hr ll ul p
scalar `hr' = m[1,1]
scalar `ll' = m[5,1]
scalar `ul' = m[6,1]
scalar `p'  = m[4,1]
quietly: putexcel set "Pooled results.xlsm", sheet("Fine_Gray") modify
        quietly: putexcel K`irow'=(`e(N_sub)'), nformat(number)
        quietly: putexcel L`irow'=(`e(N_fail)'), nformat(number)
        quietly: putexcel E`irow'=(`hr'), nformat(number_d2)
        quietly: putexcel F`irow'=(`ll'), nformat(number_d2)
        quietly: putexcel G`irow'=(`ul'), nformat(number_d2)
        quietly: putexcel AF`irow'=(`p'), nformat(number_d3)
        quietly: putexcel B`irow'=("`idg'")
        quietly: putexcel C`irow'=("pooled")
```

```
quietly: putexcel D`irow'=("`idg_name'")
tab any`idg', matcell(x), if `idg'_split!=-1
matrix list x
quietly: tab any`idg' ensidementia, matcell(y), if `idg'_split!=-1
matrix list y
quietly: putexcel set "Pooled results.xlsm", sheet("Fine_Gray") modify
quietly: putexcel M`irow'=(x[2,1]), nformat(number)
quietly: putexcel N`irow'=(y[2,1]), nformat(number)
local ++irow
}
```

*** TABLE 3 ***

```
local dg `"I63 M81 E03 K26 A46 K29 I21 I25"'
local dg_name `""Cerebral infarction" "Osteoporosis without pathological fracture" "Other hypothyroidism" "Duodenal ulcer" "Erysipelas" "Gastritis and duodenitis" "Acute myocardial infarction" "Chronic ischaemic heart disease""'
local n_dg: word count `dg'
quietly: local irow = 4
forvalues i=1/`n_dg' {
quietly: local idg : word `i' of `dg'
quietly: local idg_name : word  `i' of `dg_name'
display ""
display "`idg' followed by broad dementia, adjsuted for conventional potentially modifiable risk factors"
display ""
display "Pooled IPD-analysis"
display ""
display "$S_TIME  $S_DATE"
display ""

use "Pooled_trimmed.dta", clear
```

```
drop if cohort==3
keep if lowses!=. & obesity!=. & smoker!=. & kyselydepressio !=. & inactive!=. & married!=. & kyselydiabetes!=. & kyselyhypert!=.
count
count if ensidementia==1

stset exitpvm, id(id) failure(ensidementia) origin(time syntpvm) enter(time viimentrypvm) scale(365.25)
keep if _st==1
count
count if ensidementia==1
keep id any`idg' supu cohort ensi`idg'pvm ensidementia _st _d _origin _t _t0 lowses kyselyhypert obesity  smoker  kyselydepressio   inactive  married  kyselydiabetes ses

tab any`idg'
stsplit `idg'_split, at(0) after(time=ensi`idg'pvm)
tab `idg'_split
gen `idg'_timedep =0
replace `idg'_timedep = 1 if any`idg'==1 & `idg'_split==0
tab `idg'_timedep
drop any`idg' ensi`idg'pvm `idg'_split

stcox `idg'_timedep 2.supu#cohort, strata(cohort)
matrix a = r(table)
stcox `idg'_timedep 2.supu#cohort#married 1.ses#cohort#inactive 2.ses#cohort#inactive 1.kyselyhypert#cohort#married 1.obesity#cohort 1.obesity#1.cohort#1.smoker 1.obesity#4.cohort#1.smoker 1.smoker#cohort#kyselydepressio 1.kyselydepressio#cohort 1.inactive#cohort 0.married#cohort 1.kyselydiabetes#cohort, strata(cohort)
matrix b = r(table)
estat phtest, detail

quietly: putexcel set "Pooled results.xlsm", sheet("Table 2") modify
        *quietly: putexcel B`irow'=("`idg' `idg_name'"), nformat(number_d1)
        quietly: putexcel H`irow'=(a[1,1]), nformat(number_d1)
        quietly: putexcel I`irow'=(a[5,1]), nformat(number_d1)
```

```
						quietly: putexcel J`irow'=(a[6,1]), nformat(number_d1)

						quietly: putexcel K`irow'=(b[1,1]), nformat(number_d1)

						quietly: putexcel L`irow'=(b[5,1]), nformat(number_d1)

						quietly: putexcel M`irow'=(b[6,1]), nformat(number_d1)


				local ++irow

}
```

```
*** eMethods 2 ***

local dg `"A09 A46 E03 E05 E78 H81 I20 I21 I25 I48 I63 I65 J44 K20 K26 K29 K59 K62 K70 L30 M81 N40"'

local n_dg: word count `dg'

local dg_name `""Other gastroenteritis and colitis of infectious and unspecified origin"	"Erysipelas"	"Other hypothyroidism"	"Thyrotoxicosis [hyperthyroidism]"	"Disorders of lipoprotein metabolism and other lipidaemias"	"Disorders of vestibular function"	"Angina pectoris"	"Acute myocardial infarction"	"Chronic ischaemic heart disease"	"Atrial fibrillation and flutter"	"Cerebral infarction"	"Occlusion and stenosis of precerebral arteries, not resulting in cerebral infarction"	"Other chronic obstructive pulmonary disease"	"Oesophagitis"	"Duodenal ulcer"	"Gastritis and duodenitis"	"Other functional intestinal disorders"	"Other diseases of anus and rectum"	"Alcoholic liver disease"	"Other dermatitis"	"Osteoporosis without pathological fracture"	"Hyperplasia of prostate""'

	quietly: local irow = 7

forvalues i=1/`n_dg' {

	quietly: local idg : word `i' of `dg'

	quietly: local idg_name : word  `i' of `dg_name'

	display ""

	display "`idg' followed by broad dementia, adjusted for sex and stratified by cohort"

	display ""

	display "Normaali seuranta"

	display ""

	use "Pooled_trimmed", clear


	quietly: stset exitpvm, id(id) failure(ensidementia) origin(time syntpvm) enter(time entrypvm) scale(365.25)
```

```
        quietly: keep id any`idg' supu cohort exitpvm ensidementia syntpvm entrypvm ensi`idg'pvm  _st _d
_origin _t _t0
                tab any`idg'
                stsplit `idg'_split, at(0) after(time=ensi`idg'pvm)
                tab `idg'_split
                gen `idg'_timedep =0
                replace `idg'_timedep = 1 if any`idg'==1 & `idg'_split==0
                tab `idg'_timedep
                tab any`idg' if `idg'_split!=-1

        if "`idg'" == "N40" {
                stcox `idg'_timedep if supu==1, strata(cohort)
                }
        else {
                stcox `idg'_timedep 2.supu#cohort, strata(cohort)
                }
        matrix r = r(table)
        estat phtest, detail
        matrix m = r(phtest)
                quietly: putexcel set "Pooled results.xlsm", sheet("IPD, PH-assumptions") modify
    quietly: putexcel A`irow'="`idg'", nformat(number)
    quietly: putexcel B`irow'="`idg_name'", nformat(number)
                quietly: putexcel C`irow'=`r(p)', nformat(number)
                quietly: putexcel D`irow'=matrix(m[1,4]), nformat(number_d2)
                quietly: putexcel E`irow'=matrix(m[2,4]), nformat(number_d2)
                quietly: putexcel F`irow'=matrix(m[3,4]), nformat(number_d2)
                quietly: putexcel G`irow'=matrix(m[4,4]), nformat(number_d2)
                quietly: putexcel H`irow'=matrix(m[5,4]), nformat(number_d2)
                quietly: putexcel I`irow'=matrix(r[1,1]), nformat(number_d2)
        local ++irow
        }
```

*** TABLE A. 5 ***

```
use "Pooled_trimmattu", clear

egen byte anyA = rowmax(anyA09 anyA46)
egen ensiApvm = rowmin(ensiA09pvm ensiA46pvm)

egen byte anyE = rowmax(anyE03 anyE05 anyE78)
egen ensiEpvm = rowmin(ensiE03pvm ensiE05pvm ensiE78pvm)

egen byte anyI = rowmax(anyI20 anyI21 anyI25 anyI48 anyI63 anyI65)
egen ensiIpvm = rowmin(ensiI20pvm ensiI21pvm ensiI25pvm ensiI48pvm ensiI63pvm ensiI65pvm)

egen byte anyK = rowmax(anyK20 anyK26 anyK29 anyK59 anyK62 anyK70)
egen ensiKpvm = rowmin(ensiK20pvm ensiK26pvm ensiK29pvm ensiK59pvm ensiK62pvm ensiK70pvm)

keep id anyA* anyE* anyI* anyK* ensiA* ensiE* ensiI* ensiK* supu cohort exitpvm ensidementia syntpvm entrypvm

save "Pooled_class", replace

local dg `"A E I K"'
local n_dg: word count `dg'
local dg_name `""Chapter A, summary estimate" "Chapter E, summary estimate" "Chapter I, summary estimate" "Chapter K, summary estimate""'
		quietly: local irow = 11
		forvalues i=1/`n_dg' {
				quietly: local idg : word `i' of `dg'
				quietly: local idg_name : word  `i' of `dg_name'
				display ""
				display "`idg' followed by broad dementia, adjusted for sex and stratified by cohort"
				display ""
				display "Pooled IPD-analysis, 10-year exclusion"
```

```
display ""
display "$S_TIME  $S_DATE"
display ""

quietly: use "Pooled_class", clear
quietly: stset exitpvm, id(id) failure(ensidementia) origin(time syntpvm) enter(time entrypvm) scale(365.25)
tab any`idg'
stsplit `idg'_split, at(0) after(time=ensi`idg'pvm)
tab `idg'_split
gen `idg'_timedep =0
replace `idg'_timedep = 1 if any`idg'==1 & `idg'_split==0
tab `idg'_timedep
tab any`idg' if `idg'_split!=-1



*Poistetaan seurannan ensimmäiset 10 vuotta (tai 10v diagnoosista, jos diagnoosi oli ennen seurannan alkua mutta kuitenkin alle 10 ennen seurannan alkua)
gen ensi`idg'pvm10v = ensi`idg'pvm + round(10*365.25) if ensi`idg'pvm<entrypvm
replace ensi`idg'pvm10v = entrypvm if ensi`idg'pvm10v<entrypvm
replace _t0 = (ensi`idg'pvm10v-syntpvm)/365.25 if ensi`idg'pvm10v!=.
replace _t0 = _t0 + 10 if ensi`idg'pvm10v==.
drop if _t0>=_t
quietly: stdescribe
quietly: local failures = `r(N_fail)'
stcox `idg'_timedep 2.supu#cohort, strata(cohort)
quietly: matrix m = r(table)
scalar `hr' = m[1,1]
scalar `ll' = m[5,1]
scalar `ul' = m[6,1]
scalar `p'  = m[4,1]
quietly: putexcel set "Pooled results.xlsm", sheet("summary estimate") modify
        quietly: putexcel K`irow'=(`e(N_sub)'), nformat(number)
        quietly: putexcel L`irow'=(`e(N_fail)'), nformat(number)
```

```
quietly: putexcel E`irow'=(`hr'), nformat(number_d2)
quietly: putexcel F`irow'=(`ll'), nformat(number_d2)
quietly: putexcel G`irow'=(`ul'), nformat(number_d2)
quietly: putexcel Y`irow'=(`p'), nformat(number_d3)
quietly: putexcel B`irow'=("   `idg'_10y_exclusion")
quietly: putexcel C`irow'=("pooled")
quietly: putexcel D`irow'=("   Dementia occurring more than 10 years after the hospitalisation")
tab any`idg', matcell(x), if `idg'_split!=-1
matrix list x
quietly: tab any`idg' ensidementia, matcell(y), if `idg'_split!=-1
matrix list y
quietly: putexcel set "Pooled results.xlsm", sheet("summary estimate") modify
quietly: putexcel M`irow'=(x[2,1]), nformat(number)
quietly: putexcel N`irow'=(y[2,1]), nformat(number)
local ++irow
local ++irow
}
```

```
*** TABLE A. 6 ***
*Please see the code for Figure 2.
```

```
***	TABLE A. 7 ***
use "Pooled_trimmed", clear

replace obesity = 0 if cohort==3
replace bmi_who = 0 if cohort==3
keep if ses!=. & obesity!=. & smoker!=. & kyselydepressio !=. & inactive!=. & married!=. & kyselydiabetes!=. & kyselyhypert!=.
stset exitpvm, id(id) failure(ensidementia) origin(time syntpvm) enter(time viimentrypvm) scale(365.25)
keep if _st==1
```

```
count
count if ensidementia==1
replace obesity = . if cohort==3
replace bmi_who = . if cohort==3

stsplit age65, at(64.999)
recode age65 64.999=1

*Low education
preserve
keep if age65==0
gen low_vs_highses = ses
recode low_vs_highses 2=. 3=0
tab low_vs_highses _d, matcell(n)
stcox b3.ses 2.supu#cohort, strata(cohort)
matrix a = r(table)
local irow = 6
quietly: putexcel set "Pooled results.xlsm", sheet("Conventional_early") modify
                quietly: putexcel E`irow'=n[2,1]+n[2,2], nformat(number_d1)
                quietly: putexcel F`irow'=n[2,2], nformat(number_d1)
                quietly: putexcel G`irow'=n[1,1]+n[1,2], nformat(number_d1)
                quietly: putexcel H`irow'=(n[1,2]), nformat(number_d1)
                quietly: putexcel Y`irow'=(a[1,1]), nformat(number_d1)
                quietly: putexcel Z`irow'=(a[5,1]), nformat(number_d1)
                quietly: putexcel AA`irow'=(a[6,1]), nformat(number_d1)
restore

preserve
keep if age65==1
gen low_vs_highses = ses
recode low_vs_highses 2=. 3=0
tab low_vs_highses _d, matcell(n)
stcox b3.ses 2.supu#cohort, strata(cohort)
```

```
matrix a = r(table)
local irow = 6
quietly: putexcel set "Pooled results.xlsm", sheet("Conventional_late") modify
        quietly: putexcel E`irow'=n[2,1]+n[2,2], nformat(number_d1)
        quietly: putexcel F`irow'=n[2,2], nformat(number_d1)
        quietly: putexcel G`irow'=n[1,1]+n[1,2], nformat(number_d1)
        quietly: putexcel H`irow'=(n[1,2]), nformat(number_d1)
        quietly: putexcel Y`irow'=(a[1,1]), nformat(number_d1)
        quietly: putexcel Z`irow'=(a[5,1]), nformat(number_d1)
        quietly: putexcel AA`irow'=(a[6,1]), nformat(number_d1)
restore

*Midlife hypertension (ne pudotetaan pois, joiden seuranta alkaa vasta 65-vuotiaana tai myöhemmin)
use "Pooled_trimmed", clear

replace obesity = 0 if cohort==3
replace bmi_who = 0 if cohort==3
keep if ses!=. & obesity!=. & smoker!=. & kyselydepressio !=. & inactive!=. & married!=. & kyselydiabetes!=. & kyselyhypert!=.
stset exitpvm, id(id) failure(ensidementia) origin(time syntpvm) enter(time viimentrypvm) scale(365.25)
keep if _st==1
count
count if ensidementia==1
replace obesity = . if cohort==3
replace bmi_who = . if cohort==3

drop if _t0>=65

stsplit age65, at(64.999)
recode age65 64.999=1

preserve
keep if age65==0
```

```
tab kyselyhypert _d, matcell(n)
stcox kyselyhypert 2.supu#cohort, strata(cohort)
matrix a = r(table)
local irow = 7
quietly: putexcel set "Pooled results.xlsm", sheet("Conventional_early") modify
                    quietly: putexcel E`irow'=n[2,1]+n[2,2], nformat(number_d1)
                    quietly: putexcel F`irow'=n[2,2], nformat(number_d1)
                    quietly: putexcel G`irow'=n[1,1]+n[1,2], nformat(number_d1)
                    quietly: putexcel H`irow'=(n[1,2]), nformat(number_d1)
                    quietly: putexcel Y`irow'=(a[1,1]), nformat(number_d1)
                    quietly: putexcel Z`irow'=(a[5,1]), nformat(number_d1)
                    quietly: putexcel AA`irow'=(a[6,1]), nformat(number_d1)
restore

preserve
keep if age65==1
tab kyselyhypert _d, matcell(n)
stcox kyselyhypert 2.supu#cohort, strata(cohort)
matrix a = r(table)
local irow = 7
quietly: putexcel set "Pooled results.xlsm", sheet("Conventional_late") modify
                    quietly: putexcel E`irow'=n[2,1]+n[2,2], nformat(number_d1)
                    quietly: putexcel F`irow'=n[2,2], nformat(number_d1)
                    quietly: putexcel G`irow'=n[1,1]+n[1,2], nformat(number_d1)
                    quietly: putexcel H`irow'=(n[1,2]), nformat(number_d1)
                    quietly: putexcel Y`irow'=(a[1,1]), nformat(number_d1)
                    quietly: putexcel Z`irow'=(a[5,1]), nformat(number_d1)
                    quietly: putexcel AA`irow'=(a[6,1]), nformat(number_d1)
restore

*Midlife obesity (ne pudotetaan pois, joiden seuranta alkaa vasta 65-vuotiaana tai myöhemmin)
preserve
keep if age65==0
```

```
tab obesity _d, matcell(n), if cohort!=3
stcox obesity 2.supu#cohort, strata(cohort), if cohort!=3
matrix a = r(table)
local irow = 8
quietly: putexcel set "Pooled results.xlsm", sheet("Conventional_early") modify
                                quietly: putexcel E`irow'=n[2,1]+n[2,2], nformat(number_d1)
                                quietly: putexcel F`irow'=n[2,2], nformat(number_d1)
                                quietly: putexcel G`irow'=n[1,1]+n[1,2], nformat(number_d1)
                                quietly: putexcel H`irow'=(n[1,2]), nformat(number_d1)
                                quietly: putexcel Y`irow'=(a[1,1]), nformat(number_d1)
                                quietly: putexcel Z`irow'=(a[5,1]), nformat(number_d1)
                                quietly: putexcel AA`irow'=(a[6,1]), nformat(number_d1)
restore

preserve
keep if age65==1

tab obesity _d, matcell(n), if cohort!=3
stcox obesity 2.supu#cohort, strata(cohort), if cohort!=3
matrix a = r(table)
local irow = 8
quietly: putexcel set "Pooled results.xlsm", sheet("Conventional_late") modify
                                quietly: putexcel E`irow'=n[2,1]+n[2,2], nformat(number_d1)
                                quietly: putexcel F`irow'=n[2,2], nformat(number_d1)
                                quietly: putexcel G`irow'=n[1,1]+n[1,2], nformat(number_d1)
                                quietly: putexcel H`irow'=(n[1,2]), nformat(number_d1)
                                quietly: putexcel Y`irow'=(a[1,1]), nformat(number_d1)
                                quietly: putexcel Z`irow'=(a[5,1]), nformat(number_d1)
                                quietly: putexcel AA`irow'=(a[6,1]), nformat(number_d1)
restore

*Smoking
```

```
use "Pooled_trimmed", clear
replace obesity = 0 if cohort==3
keep if ses!=. & obesity!=. & smoker!=. & kyselydepressio !=. & inactive!=. & married!=. & kyselydiabetes!=. & kyselyhypert!=.
stset exitpvm, id(id) failure(ensidementia) origin(time syntpvm) enter(time viimentrypvm) scale(365.25)
keep if _st==1

stsplit age65, at(64.999)
recode age65 64.999=1

preserve
keep if age65==0

tab smoker _d, matcell(n)
stcox smoker 2.supu#cohort, strata(cohort)
matrix a = r(table)
local irow = 9
quietly: putexcel set "Pooled results.xlsm", sheet("Conventional_early") modify
        quietly: putexcel E`irow'=n[2,1]+n[2,2], nformat(number_d1)
        quietly: putexcel F`irow'=n[2,2], nformat(number_d1)
        quietly: putexcel G`irow'=n[1,1]+n[1,2], nformat(number_d1)
        quietly: putexcel H`irow'=(n[1,2]), nformat(number_d1)
        quietly: putexcel Y`irow'=(a[1,1]), nformat(number_d1)
        quietly: putexcel Z`irow'=(a[5,1]), nformat(number_d1)
        quietly: putexcel AA`irow'=(a[6,1]), nformat(number_d1)
restore

preserve
keep if age65==1

tab smoker _d, matcell(n)
stcox smoker 2.supu#cohort, strata(cohort)
matrix a = r(table)
```

```
local irow = 9
quietly: putexcel set "Pooled results.xlsm", sheet("Conventional_late") modify
                                        quietly: putexcel E`irow'=n[2,1]+n[2,2], nformat(number_d1)
                                        quietly: putexcel F`irow'=n[2,2], nformat(number_d1)
                                        quietly: putexcel G`irow'=n[1,1]+n[1,2], nformat(number_d1)
                                        quietly: putexcel H`irow'=(n[1,2]), nformat(number_d1)
                                        quietly: putexcel Y`irow'=(a[1,1]), nformat(number_d1)
                                        quietly: putexcel Z`irow'=(a[5,1]), nformat(number_d1)
                                        quietly: putexcel AA`irow'=(a[6,1]), nformat(number_d1)
restore


*Depression
preserve
keep if age65==0
tab kyselydepressio _d, matcell(n)
stcox kyselydepressio 2.supu#cohort, strata(cohort)
matrix a = r(table)
local irow = 10
quietly: putexcel set "Pooled results.xlsm", sheet("Conventional_early") modify
                                        quietly: putexcel E`irow'=n[2,1]+n[2,2], nformat(number_d1)
                                        quietly: putexcel F`irow'=n[2,2], nformat(number_d1)
                                        quietly: putexcel G`irow'=n[1,1]+n[1,2], nformat(number_d1)
                                        quietly: putexcel H`irow'=(n[1,2]), nformat(number_d1)
                                        quietly: putexcel Y`irow'=(a[1,1]), nformat(number_d1)
                                        quietly: putexcel Z`irow'=(a[5,1]), nformat(number_d1)
                                        quietly: putexcel AA`irow'=(a[6,1]), nformat(number_d1)
restore

preserve
keep if age65==1
tab kyselydepressio _d, matcell(n)
stcox kyselydepressio 2.supu#cohort, strata(cohort)
```

```
matrix a = r(table)
local irow = 10
quietly: putexcel set "Pooled results.xlsm", sheet("Conventional_late") modify
                quietly: putexcel E`irow'=n[2,1]+n[2,2], nformat(number_d1)
                quietly: putexcel F`irow'=n[2,2], nformat(number_d1)
                quietly: putexcel G`irow'=n[1,1]+n[1,2], nformat(number_d1)
                quietly: putexcel H`irow'=(n[1,2]), nformat(number_d1)
                quietly: putexcel Y`irow'=(a[1,1]), nformat(number_d1)
                quietly: putexcel Z`irow'=(a[5,1]), nformat(number_d1)
                quietly: putexcel AA`irow'=(a[6,1]), nformat(number_d1)
restore



*Physical inactivity
preserve
keep if age65==0
tab inactive _d, matcell(n)
stcox inactive 2.supu#cohort, strata(cohort)
matrix a = r(table)
local irow = 11
quietly: putexcel set "Pooled results.xlsm", sheet("Conventional_early") modify
                quietly: putexcel E`irow'=n[2,1]+n[2,2], nformat(number_d1)
                quietly: putexcel F`irow'=n[2,2], nformat(number_d1)
                quietly: putexcel G`irow'=n[1,1]+n[1,2], nformat(number_d1)
                quietly: putexcel H`irow'=(n[1,2]), nformat(number_d1)
                quietly: putexcel Y`irow'=(a[1,1]), nformat(number_d1)
                quietly: putexcel Z`irow'=(a[5,1]), nformat(number_d1)
                quietly: putexcel AA`irow'=(a[6,1]), nformat(number_d1)
restore

preserve
keep if age65==1
```

```
tab inactive _d, matcell(n)
stcox inactive 2.supu#cohort, strata(cohort)
matrix a = r(table)
local irow = 11
quietly: putexcel set "Pooled results.xlsm", sheet("Conventional_late") modify
                    quietly: putexcel E`irow'=n[2,1]+n[2,2], nformat(number_d1)
                    quietly: putexcel F`irow'=n[2,2], nformat(number_d1)
                    quietly: putexcel G`irow'=n[1,1]+n[1,2], nformat(number_d1)
                    quietly: putexcel H`irow'=(n[1,2]), nformat(number_d1)
                    quietly: putexcel Y`irow'=(a[1,1]), nformat(number_d1)
                    quietly: putexcel Z`irow'=(a[5,1]), nformat(number_d1)
                    quietly: putexcel AA`irow'=(a[6,1]), nformat(number_d1)
restore

*Social isolation by proxy
preserve
keep if age65==0
tab married _d, matcell(n)
stcox 0.married 2.supu#cohort, strata(cohort)
matrix a = r(table)
local irow = 12
quietly: putexcel set "Pooled results.xlsm", sheet("Conventional_early") modify
                    quietly: putexcel E`irow'=n[2,1]+n[2,2], nformat(number_d1)
                    quietly: putexcel F`irow'=n[2,2], nformat(number_d1)
                    quietly: putexcel G`irow'=n[1,1]+n[1,2], nformat(number_d1)
                    quietly: putexcel H`irow'=(n[1,2]), nformat(number_d1)
                    quietly: putexcel Y`irow'=(a[1,1]), nformat(number_d1)
                    quietly: putexcel Z`irow'=(a[5,1]), nformat(number_d1)
                    quietly: putexcel AA`irow'=(a[6,1]), nformat(number_d1)
restore

preserve
keep if age65==1
```

```
tab married _d, matcell(n)
stcox 0.married 2.supu#cohort, strata(cohort)
matrix a = r(table)
local irow = 12
quietly: putexcel set "Pooled results.xlsm", sheet("Conventional_late") modify
                quietly: putexcel E`irow'=n[2,1]+n[2,2], nformat(number_d1)
                quietly: putexcel F`irow'=n[2,2], nformat(number_d1)
                quietly: putexcel G`irow'=n[1,1]+n[1,2], nformat(number_d1)
                quietly: putexcel H`irow'=(n[1,2]), nformat(number_d1)
                quietly: putexcel Y`irow'=(a[1,1]), nformat(number_d1)
                quietly: putexcel Z`irow'=(a[5,1]), nformat(number_d1)
                quietly: putexcel AA`irow'=(a[6,1]), nformat(number_d1)
restore


*Diabetes
preserve
keep if age65==0
tab kyselydiabetes _d, matcell(n)
stcox kyselydiabetes 2.supu#cohort, strata(cohort)
matrix a = r(table)
local irow = 13
quietly: putexcel set "Pooled results.xlsm", sheet("Conventional_early") modify
                quietly: putexcel E`irow'=n[2,1]+n[2,2], nformat(number_d1)
                quietly: putexcel F`irow'=n[2,2], nformat(number_d1)
                quietly: putexcel G`irow'=n[1,1]+n[1,2], nformat(number_d1)
                quietly: putexcel H`irow'=(n[1,2]), nformat(number_d1)
                quietly: putexcel Y`irow'=(a[1,1]), nformat(number_d1)
                quietly: putexcel Z`irow'=(a[5,1]), nformat(number_d1)
                quietly: putexcel AA`irow'=(a[6,1]), nformat(number_d1)
restore

preserve
```

```
keep if age65==1

tab kyselydiabetes _d, matcell(n)

stcox kyselydiabetes 2.supu#cohort, strata(cohort)

matrix a = r(table)

local irow = 13

quietly: putexcel set "Pooled results.xlsm", sheet("Conventional_late") modify

quietly: putexcel E`irow'=n[2,1]+n[2,2], nformat(number_d1)

quietly: putexcel F`irow'=n[2,2], nformat(number_d1)

quietly: putexcel G`irow'=n[1,1]+n[1,2], nformat(number_d1)

quietly: putexcel H`irow'=(n[1,2]), nformat(number_d1)

quietly: putexcel Y`irow'=(a[1,1]), nformat(number_d1)

quietly: putexcel Z`irow'=(a[5,1]), nformat(number_d1)

quietly: putexcel AA`irow'=(a[6,1]), nformat(number_d1)

restore
```

```
*** TABLE A. 8 ***

local dg `"I63 M81 E03 K26 A46 K29 I21 I25"'

local dg_name `""Cerebral infarction" "Osteoporosis without pathological fracture" "Other hypothyroidism" "Duodenal ulcer" "Erysipelas" "Gastritis and duodenitis" "Acute myocardial infarction" "Chronic ischaemic heart disease""'

local n_dg: word count `dg'

quietly: local irow = 4

forvalues i=1/`n_dg' {

quietly: local idg : word `i' of `dg'

quietly: local idg_name : word  `i' of `dg_name'

display ""

display "`idg' followed by broad dementia, relative excess risk attributable to modifiable risk factors"

display ""

display "Pooled IPD-analysis"

display ""

display "$S_TIME  $S_DATE"

display ""
```

```
use "WHII_apoe", clear

keep if apoe_bin!=.

*Models A and C
quietly: stset exitpvm, id(id) failure(ensidementia) origin(time syntpvm) enter(time entrypvm) scale(365.25)
keep if _st==1
keep id any`idg' supu cohort ensi`idg'pvm _st _d _origin _t _t0 apoe_bin ensidementia

tempname Ncont Dcont Nexp Dexp
count if any`idg'==0
scalar `Ncont' = r(N)
count if any`idg'==0 & ensidementia==1
scalar `Dcont' = r(N)
count if any`idg'==1
scalar `Nexp' = r(N)
count if any`idg'==1 & ensidementia==1
scalar `Dexp' = r(N)

stsplit `idg'_split, at(0) after(time=ensi`idg'pvm)
tab `idg'_split
gen `idg'_timedep =0
replace `idg'_timedep = 1 if any`idg'==1 & `idg'_split==0
tab `idg'_timedep
drop any`idg' ensi`idg'pvm `idg'_split

stcox `idg'_timedep i.supu
matrix a = r(table)
stcox `idg'_timedep  i.supu apoe_bin
matrix c = r(table)

quietly: putexcel set "Pooled results.xlsm", sheet("eTable 4") modify
```

```
				quietly: putexcel M`irow'=(a[1,1]), nformat(number_d1)
				quietly: putexcel N`irow'=(a[5,1]), nformat(number_d1)
				quietly: putexcel O`irow'=(a[6,1]), nformat(number_d1)
				quietly: putexcel P`irow'=(c[1,1]), nformat(number_d1)
				quietly: putexcel Q`irow'=(c[5,1]), nformat(number_d1)
				quietly: putexcel R`irow'=(c[6,1]), nformat(number_d1)
				quietly: putexcel E`irow'=`Ncont', nformat(number_d1)
				quietly: putexcel F`irow'=`Dcont', nformat(number_d1)
				quietly: putexcel G`irow'=`Nexp', nformat(number_d1)
				quietly: putexcel H`irow'=`Dexp', nformat(number_d1)
		local ++irow
}
```

```
*** TABLE A. 9 ***
/*
```

Produced with Excel using formula 19 in Scosyrev E, Glimm E. Power analysis for multivariable Cox regression models. Stat Med. 2019;38(1):88-99.

The overall survival probability and numbers of participants exposed to the index diseases were retrieved from the actual data.

VIF was estimated using formula VIF=1/(1-R_squared). R_squared was derived from actual correlations between sex (the adjusting variable) and the index diseases. The highest estimated VIF was 1.0125833 which was rounded upwards to a conservative estimate of VIF=1.02 used in all calculations.

```
*/
```

```
*** Fig. A. 2 ***
```

*Code for eFigure 2 modified from: Royston P, Lambert PC. Flexible Parametric Survival Analysis Using Stata: Beyond the Cox Model. College Station, TX: Stata Press; 2011.

```
local dg `"E03 I63 J44 K59 M81"'
local n_dg: word count `dg'
```

```
local dg_name `""Other hypothyroidism" "Cerebral infarction" "Other chronic obstructive pulmonary disease" "Other functional intestinal disorders" "Osteoporosis without pathological fracture""'

forvalues i=1/`n_dg' {
		quietly: local idg : word `i' of `dg'
		quietly: local idg_name : word  `i' of `dg_name'


		use "Pooled_trimmed", clear
		quietly: keep id any`idg' supu cohort exitpvm ensidementia syntpvm entrypvm ensi`idg'pvm


		local j = 1
		foreach cohort in FPS HeSSup STW WHII {
				gen `cohort'supu=0
				replace `cohort'supu=1 if supu==2 & cohort==`j'
				local ++j
				}
		tabulate cohort, gen(dummycohort)


		quietly: stset exitpvm, id(id) failure(ensidementia) origin(time syntpvm) enter(time entrypvm)
scale(365.25)
		tab any`idg'
				stsplit `idg'_split, at(0) after(time=ensi`idg'pvm)
				tab `idg'_split
				gen `idg'_timedep =0
				replace `idg'_timedep = 1 if any`idg'==1 & `idg'_split==0
				tab `idg'_timedep
				tab any`idg' if `idg'_split!=-1


		stcox `idg'_timedep FPSsupu HeSSupsupu STWsupu WHIIsupu, strata(cohort)
		capture drop *sca*
		predict sca*, scaledsch
		running sca1 _t if _d==1, gen(smooth_sca) gense(smooth_sca_se) nodraw
		gen smooth_esca = exp(smooth_sca)
		gen smooth_esca_lci = exp(smooth_sca - 1.96*smooth_sca_se)
		gen smooth_esca_uci = exp(smooth_sca + 1.96*smooth_sca_se)
```

```
local betaround: di %7.2f exp(_b[`idg'_timedep])
tokenize "`betaround'"
di "`1'"
twoway (rarea smooth_esca_lci smooth_esca_uci _t, pstyle(ci) sort yaxis(1 2)) ///
	(line smooth_esca _t, sort clpattern(solid)) ///
	(function y = 1, lpattern(shortdash) range(_t)) ///
	(function y = `1', lpattern(longdash) range(_t)), ///
	legend(order(2 "Exponentiated scaled Schoenfeld residuals" 1 "95% confidence interval" 4 "Overall hazard ratio   ") holes(2) size(small)) ///
	title("`idg' `idg_name'" "and dementia", size(medsmall)) ///
	ytitle("Exponentiated scaled Schoenfeld residuals", size(small)) ///
	xtitle("Age (years)", size(small)) ///
	yscale(log range(0.1 100)) ///
	scheme(sj) graphregion(color(white)) ///
	ylabel(0.1 1 10 100, labsize(medsmall) angle(0)) ///
	ylabel("`1'", angle(0) axis(2))
graph save sca_`idg'.gph, replace
}
```

```
*** Fig. A. 5 ***
```

```
use "Pooled_trimmattu", clear
local dg `"A46 E03 I21 I25 I63 K26 K29 M81"'
local dg_name `""Erysipelas" "Other hypothyroidism"	"Acute myocardial infarction" "Chronic ischaemic heart disease" "Cerebral infarction" "Duodenal ulcer" "Gastritis and duodenitis" "Osteoporosis without pathological fracture""'
local n_dg: word count `dg'
local column "F G H I J K L M N O P Q R S T U V W X Y Z AA AB AC AD AE AF AG AH AI AJ AK AL AM AN AO AP AQ AR AS AT AU AV AW AX AY AZ BA"

forvalues i = 1/`n_dg' {
	quietly: local dg1: word  `i' of `dg'
	quietly: local dg1_name : word  `i' of `dg_name'
	local row = `i' + 3
```

```
quietly: putexcel set "Pooled results.xlsm", sheet("new odds ratios") modify
        quietly: putexcel A`row'="`dg1'", nformat(number)
        quietly: putexcel B`row'="`dg1_name'", nformat(number)
local k = `i' + 1
forvalues j = `k'/`n_dg' {
        quietly: local dg2: word  `j' of `dg'
        quietly: local col : word `j' of `column'
        di ""
        di "Odds ratio for `dg1' and `dg2'"
        cc any`dg1' any`dg2', by(cohort)
        quietly: local output: di %7.2f `r(or)' " ("% 7.2f `r(lb_or)' "-" %7.2f
`r(ub_or)' ")"
        quietly: putexcel `col'`row'="`output'"
        }
}
```

*** Fig. A. 6 ***

```
local dg `"I63 M81 E03 K26 A46 K29 I21 I25"'
local dg_name `""Cerebral infarction" "Osteoporosis without pathological fracture" "Other hypothyroidism" "Duodenal ulcer" "Erysipelas" "Gastritis and duodenitis" "Acute myocardial infarction" "Chronic ischaemic heart disease""'
local n_dg: word count `dg'
quietly: local irow = 8
forvalues i=1/`n_dg' {
    quietly: local idg : word `i' of `dg'
    quietly: local idg_name : word  `i' of `dg_name'
    display ""
    display "`idg' followed by broad dementia, adjusted for sex and stratified by cohort"
    display ""
    display "Pooled IPD-analysis, all diagnoses vs primary diagnoses only"
    display ""
    display "$S_TIME  $S_DATE"
```

```
display ""



quietly: use "Pooled_trimmed", clear
quietly: stset exitpvm, id(id) failure(ensidementia) origin(time syntpvm) enter(time entrypvm) scale(365.25)
quietly: keep id any`idg' supu cohort exitpvm ensidementia syntpvm entrypvm ensi`idg'pvm  _st _d _origin _t _t0
tab any`idg'
stsplit `idg'_split, at(0) after(time=ensi`idg'pvm)
tab `idg'_split
gen `idg'_timedep =0
replace `idg'_timedep = 1 if any`idg'==1 & `idg'_split==0
tab `idg'_timedep
tab any`idg' if `idg'_split!=-1

quietly: stdescribe
quietly: local failures = `r(N_fail)'
if "`idg'" == "N40" {
stcox `idg'_timedep if supu==1, strata(cohort)
}
else {
stcox `idg'_timedep 2.supu#cohort, strata(cohort)
}
quietly: matrix m = r(table)
tempname hr ll ul
scalar `hr' = m[1,1]
scalar `ll' = m[5,1]
scalar `ul' = m[6,1]
quietly: putexcel set "Pooled results.xlsm", sheet("eFIGURE 5") modify
        quietly: putexcel K`irow'=(`e(N_sub)'), nformat(number)
        quietly: putexcel L`irow'=(`e(N_fail)'), nformat(number)
        quietly: putexcel E`irow'=(`hr'), nformat(number_d2)
        quietly: putexcel F`irow'=(`ll'), nformat(number_d2)
```

```
quietly: putexcel G`irow'=(`ul'), nformat(number_d2)
quietly: putexcel B`irow'=("`idg'")
quietly: putexcel C`irow'=("pooled")
quietly: putexcel D`irow'=("`idg_name'")
tab any`idg', matcell(x), if `idg'_split!=-1
matrix list x
quietly: tab any`idg' ensidementia, matcell(y), if `idg'_split!=-1
matrix list y
quietly: putexcel set "Pooled results.xlsm", sheet("eFIGURE 5") modify
quietly: putexcel M`irow'=(x[2,1]), nformat(number)
quietly: putexcel N`irow'=(y[2,1]), nformat(number)

local ++irow

quietly: use "Pooled_trimmed_lisäyksiä", clear
drop if cohort==4
quietly: stset exitpvm, id(id) failure(ensidementia) origin(time syntpvm) enter(time entrypvm) scale(365.25)
quietly: keep id anyp`idg' supu cohort exitpvm ensidementia syntpvm entrypvm ensip`idg'pvm  _st _d _origin _t _t0
tab anyp`idg'
stsplit `idg'_split, at(0) after(time=ensip`idg'pvm)
tab `idg'_split
gen `idg'_timedep =0
replace `idg'_timedep = 1 if anyp`idg'==1 & `idg'_split==0
tab `idg'_timedep
tab anyp`idg' if `idg'_split!=-1

quietly: stdescribe
quietly: local failures = `r(N_fail)'
if "`idg'" == "N40" {
stcox `idg'_timedep if supu==1, strata(cohort)
}
else {
```

```
stcox `idg'_timedep 2.supu#cohort, strata(cohort)
}
quietly: matrix m = r(table)
tempname hr ll ul
scalar `hr' = m[1,1]
scalar `ll' = m[5,1]
scalar `ul' = m[6,1]
quietly: putexcel set "Pooled results.xlsm", sheet("eFIGURE 5") modify
        quietly: putexcel K`irow'=(`e(N_sub)'), nformat(number)
        quietly: putexcel L`irow'=(`e(N_fail)'), nformat(number)
        quietly: putexcel E`irow'=(`hr'), nformat(number_d2)
        quietly: putexcel F`irow'=(`ll'), nformat(number_d2)
        quietly: putexcel G`irow'=(`ul'), nformat(number_d2)
        quietly: putexcel B`irow'=("`idg'")
        quietly: putexcel C`irow'=("pooled (primary diagnoses only)")
        quietly: putexcel D`irow'=("`idg_name'")
tab anyp`idg', matcell(x), if `idg'_split!=-1
matrix list x
quietly: tab anyp`idg' ensidementia, matcell(y), if `idg'_split!=-1
matrix list y
quietly: putexcel set "Pooled results.xlsm", sheet("eFIGURE 5") modify
        quietly: putexcel M`irow'=(x[2,1]), nformat(number)
        quietly: putexcel N`irow'=(y[2,1]), nformat(number)
local ++irow
local ++irow
}
```

*** Fig. A 7. ***

```
local dg `"I63 M81 E03 K26 A46 K29 I21 I25"'
local dg_name `""Cerebral infarction" "Osteoporosis without pathological fracture" "Other hypothyroidism" "Duodenal ulcer" "Erysipelas" "Gastritis and duodenitis" "Acute myocardial infarction" "Chronic ischaemic heart disease""'
local n_dg: word count `dg'
```

```
quietly: local irow = 8
	forvalues i=1/`n_dg' {
		quietly: local idg : word `i' of `dg'
		quietly: local idg_name : word  `i' of `dg_name'
		display ""
		display "`idg' followed by broad dementia, adjusted for sex and stratified by cohort"
		display ""
		display "Pooled IPD-analysis, 10-year exclusion"
		display ""
		display "$S_TIME  $S_DATE"
		display ""


		quietly: use "Pooled_trimmed", clear
		quietly: stset exitpvm, id(id) failure(ensidementia) origin(time syntpvm) enter(time entrypvm) scale(365.25)
		quietly: keep id any`idg' supu cohort exitpvm ensidementia syntpvm entrypvm ensi`idg'pvm  _st _d _origin _t _t0
		tab any`idg'
		stsplit `idg'_split, at(0) after(time=ensi`idg'pvm)
		tab `idg'_split
		gen `idg'_timedep =0
		replace `idg'_timedep = 1 if any`idg'==1 & `idg'_split==0
		tab `idg'_timedep
		tab any`idg' if `idg'_split!=-1


		quietly: stdescribe
		quietly: local failures = `r(N_fail)'
		if "`idg'" == "N40" {
		stcox `idg'_timedep if supu==1, strata(cohort)
		}
		else {
		stcox `idg'_timedep 2.supu#cohort, strata(cohort)
		}
		quietly: matrix m = r(table)
```

```
tempname hr ll ul
scalar `hr' = m[1,1]
scalar `ll' = m[5,1]
scalar `ul' = m[6,1]
quietly: putexcel set "Pooled results.xlsm", sheet("eFIGURE 6") modify
		quietly: putexcel K`irow'=(`e(N_sub)'), nformat(number)
		quietly: putexcel L`irow'=(`e(N_fail)'), nformat(number)
		quietly: putexcel E`irow'=(`hr'), nformat(number_d2)
		quietly: putexcel F`irow'=(`ll'), nformat(number_d2)
		quietly: putexcel G`irow'=(`ul'), nformat(number_d2)
		quietly: putexcel B`irow'=("`idg'")
		quietly: putexcel C`irow'=("pooled")
		quietly: putexcel D`irow'=("`idg_name'")
		quietly: putexcel U`irow'=("normal")
tab any`idg', matcell(x), if `idg'_split!=-1
matrix list x
quietly: tab any`idg' ensidementia, matcell(y), if `idg'_split!=-1
matrix list y
quietly: putexcel set "Pooled results.xlsm", sheet("eFIGURE 6") modify
		quietly: putexcel M`irow'=(x[2,1]), nformat(number)
		quietly: putexcel N`irow'=(y[2,1]), nformat(number)
local ++irow

drop if ensi`idg'pvm < entrypvm
quietly: stdescribe
quietly: local failures = `r(N_fail)'
if "`idg'" == "N40" {
stcox `idg'_timedep if supu==1, strata(cohort)
}
else {
stcox `idg'_timedep 2.supu#cohort, strata(cohort)
}
quietly: matrix m = r(table)
```

```
tempname hr ll ul
scalar `hr' = m[1,1]
scalar `ll' = m[5,1]
scalar `ul' = m[6,1]
quietly: putexcel set "Pooled results.xlsm", sheet("eFIGURE 6") modify
        quietly: putexcel K`irow'=(`e(N_sub)'), nformat(number)
        quietly: putexcel L`irow'=(`e(N_fail)'), nformat(number)
        quietly: putexcel E`irow'=(`hr'), nformat(number_d2)
        quietly: putexcel F`irow'=(`ll'), nformat(number_d2)
        quietly: putexcel G`irow'=(`ul'), nformat(number_d2)
        quietly: putexcel B`irow'=("`idg'")
        quietly: putexcel C`irow'=("pooled")
        quietly: putexcel D`irow'=("`idg_name'")
        quietly: putexcel U`irow'=("incident only")
tab any`idg', matcell(x), if `idg'_split!=-1
matrix list x
quietly: tab any`idg' ensidementia, matcell(y), if `idg'_split!=-1
matrix list y
quietly: putexcel set "Pooled results.xlsm", sheet("eFIGURE 6") modify
        quietly: putexcel M`irow'=(x[2,1]), nformat(number)
        quietly: putexcel N`irow'=(y[2,1]), nformat(number)
local ++irow


local ++irow
}
```

```
*** Fig. A. 8 ***


local dg `"I63 M81 E03 K26 A46 K29 I21 I25"'
local dg_name `""Cerebral infarction" "Osteoporosis without pathological fracture" "Other hypothyroidism" "Duodenal ulcer" "Erysipelas" "Gastritis and duodenitis" "Acute myocardial infarction" "Chronic ischaemic heart disease""'
local n_dg: word count `dg'
```

```
quietly: local irow = 8
        forvalues i=1/`n_dg' {
                quietly: local idg : word `i' of `dg'
                quietly: local idg_name : word  `i' of `dg_name'
                display ""
                display "`idg' followed by broad dementia, adjusted for sex and stratified by cohort"
                display ""
                display "Pooled IPD-analysis, all diagnoses vs primary diagnoses only"
                display ""
                display "$S_TIME  $S_DATE"
                display ""



                quietly: use "Pooled_trimmed", clear
                quietly: stset exitpvm, id(id) failure(ensidementia) origin(time syntpvm) enter(time
entrypvm) scale(365.25)
                quietly: keep id any`idg' supu cohort exitpvm ensidementia syntpvm entrypvm
ensi`idg'pvm  _st _d _origin _t _t0
                tab any`idg'
                stsplit `idg'_split, at(0) after(time=ensi`idg'pvm)
                tab `idg'_split
                gen `idg'_timedep =0
                replace `idg'_timedep = 1 if any`idg'==1 & `idg'_split==0
                tab `idg'_timedep
                tab any`idg' if `idg'_split!=-1

                quietly: stdescribe
                quietly: local failures = `r(N_fail)'
                if "`idg'" == "N40" {
                stcox `idg'_timedep if supu==1, strata(cohort)
                }
                else {
                stcox `idg'_timedep 2.supu#cohort, strata(cohort)
                }
```

```
quietly: matrix m = r(table)
tempname hr ll ul
scalar `hr' = m[1,1]
scalar `ll' = m[5,1]
scalar `ul' = m[6,1]
quietly: putexcel set "Pooled results.xlsm", sheet("eFIGURE 7") modify
        quietly: putexcel K`irow'=(`e(N_sub)'), nformat(number)
        quietly: putexcel L`irow'=(`e(N_fail)'), nformat(number)
        quietly: putexcel E`irow'=(`hr'), nformat(number_d2)
        quietly: putexcel F`irow'=(`ll'), nformat(number_d2)
        quietly: putexcel G`irow'=(`ul'), nformat(number_d2)
        quietly: putexcel B`irow'=("`idg'")
        quietly: putexcel C`irow'=("pooled")
        quietly: putexcel D`irow'=("`idg_name'")
tab any`idg', matcell(x), if `idg'_split!=-1
matrix list x
quietly: tab any`idg' ensidementia, matcell(y), if `idg'_split!=-1
matrix list y
quietly: putexcel set "Pooled results.xlsm", sheet("eFIGURE 7") modify
        quietly: putexcel M`irow'=(x[2,1]), nformat(number)
        quietly: putexcel N`irow'=(y[2,1]), nformat(number)


local ++irow


quietly: use "Pooled_trimmed", clear
drop if cohort==4 // (In the Whitehall II study, data on the type of dementia were always missing. In other cohorts, these data were always available.)
quietly: stset exitpvm, id(id) failure(ensidementia) origin(time syntpvm) enter(time entrypvm) scale(365.25)
quietly: keep id any`idg' supu cohort exitpvm ensidementia syntpvm entrypvm ensi`idg'pvm  _st _d _origin _t _t0
tab any`idg'
stsplit `idg'_split, at(0) after(time=ensi`idg'pvm)
tab `idg'_split
```

```
gen `idg'_timedep =0
replace `idg'_timedep = 1 if any`idg'==1 & `idg'_split==0
tab `idg'_timedep
tab any`idg' if `idg'_split!=-1


quietly: stdescribe
quietly: local failures = `r(N_fail)'
if "`idg'" == "N40" {
stcox `idg'_timedep if supu==1, strata(cohort)
}
else {
stcox `idg'_timedep 2.supu#cohort, strata(cohort)
}
quietly: matrix m = r(table)
tempname hr ll ul
scalar `hr' = m[1,1]
scalar `ll' = m[5,1]
scalar `ul' = m[6,1]
quietly: putexcel set "Pooled results.xlsm", sheet("eFIGURE 7") modify
        quietly: putexcel K`irow'=(`e(N_sub)'), nformat(number)
        quietly: putexcel L`irow'=(`e(N_fail)'), nformat(number)
        quietly: putexcel E`irow'=(`hr'), nformat(number_d2)
        quietly: putexcel F`irow'=(`ll'), nformat(number_d2)
        quietly: putexcel G`irow'=(`ul'), nformat(number_d2)
        quietly: putexcel B`irow'=("`idg'")
        quietly: putexcel C`irow'=("pooled")
        quietly: putexcel D`irow'=("`idg_name'")
tab any`idg', matcell(x), if `idg'_split!=-1
matrix list x
quietly: tab any`idg' ensidementia, matcell(y), if `idg'_split!=-1
matrix list y
quietly: putexcel set "Pooled results.xlsm", sheet("eFIGURE 7") modify
        quietly: putexcel M`irow'=(x[2,1]), nformat(number)
```

```
quietly: putexcel N`irow'=(y[2,1]), nformat(number)

local ++irow
local ++irow
}
```

Supplementary references

1. Lääkintöhallitus. *Classificatio Morborum et Causarum Mortis: Tauti- Ja Kuolinsyyluokitus [Classification of Diseases and Causes of Death]*. Lääkintöhallitus; 1969. http://urn.fi/URN:NBN:fi-fe201710058910

2. Lääkintöhallitus. *Tautiluokitus 1987 : Osa 1 : Systemaattinen Osa [Classification of Diseases 1987: Part 1: Systematic Part]*. Lääkintöhallitus; 1986. http://urn.fi/URN:NBN:fi-fe201701261356

3. Terveyden ja hyvinvoinnin laitos (THL). *Tautiluokitus ICD-10 [Classification of Diseases ICD-10]*. 3rd ed. Terveyden ja hyvinvoinnin laitos (THL); 2011. http://urn.fi/URN:NBN:fi-fe201205085423

4. Caamaño-Isorna F, Corral M, Montes-Martínez A, Takkouche B. Education and dementia: a meta-analytic study. *Neuroepidemiology*. 2006;26(4):226-232. doi:10.1159/000093378

5. Meng X, D'Arcy C. Education and dementia in the context of the cognitive reserve hypothesis: a systematic review with meta-analyses and qualitative analyses. *PLoS ONE*. 2012;7(6):e38268. doi:10.1371/journal.pone.0038268

6. Lennon MJ, Makkar SR, Crawford JD, Sachdev PS. Midlife Hypertension and Alzheimer's Disease: A Systematic Review and Meta-Analysis. *J Alzheimers Dis*. 2019;71(1):307-316. doi:10.3233/JAD-190474

7. Barnes DE, Yaffe K. The projected effect of risk factor reduction on Alzheimer's disease prevalence. *Lancet Neurol*. 2011;10(9):819-828. doi:10.1016/S1474-4422(11)70072-2

8. Lee CM, Woodward M, Batty GD, et al. Association of anthropometry and weight change with risk of dementia and its major subtypes: A meta-analysis consisting 2.8 million adults with 57 294 cases of dementia. *Obes Rev*. 2020;21(4):e12989. doi:10.1111/obr.12989

9. Loef M, Walach H. Midlife obesity and dementia: meta-analysis and adjusted forecast of dementia prevalence in the United States and China. *Obesity (Silver Spring)*. 2013;21(1):E51-55. doi:10.1002/oby.20037

10. Anstey KJ, von Sanden C, Salim A, O'Kearney R. Smoking as a risk factor for dementia and cognitive decline: a meta-analysis of prospective studies. *Am J Epidemiol*. 2007;166(4):367-378. doi:10.1093/aje/kwm116

11. Zhong G, Wang Y, Zhang Y, Guo JJ, Zhao Y. Smoking is associated with an increased risk of dementia: a meta-analysis of prospective cohort studies with investigation of potential effect modifiers. *PLoS ONE*. 2015;10(3):e0118333. doi:10.1371/journal.pone.0118333

12. Xu W, Tan L, Wang H-F, et al. Meta-analysis of modifiable risk factors for Alzheimer's disease. *J Neurol Neurosurg Psychiatry*. 2015;86(12):1299-1306. doi:10.1136/jnnp-2015-310548

13. Cherbuin N, Kim S, Anstey KJ. Dementia risk estimates associated with measures of depression: a systematic review and meta-analysis. *BMJ Open*. 2015;5(12):e008853. doi:10.1136/bmjopen-2015-008853

14. Kivimäki M, Singh-Manoux A, Pentti J, et al. Physical inactivity, cardiometabolic disease, and risk of dementia: an individual-participant meta-analysis. *BMJ*. 2019;365:l1495. doi:10.1136/bmj.l1495

15. Hamer M, Chida Y. Physical activity and risk of neurodegenerative disease: a systematic review of prospective evidence. *Psychol Med*. 2009;39(1):3-11. doi:10.1017/S0033291708003681

16. Kuiper JS, Zuidersma M, Oude Voshaar RC, et al. Social relationships and risk of dementia: A systematic review and meta-analysis of longitudinal cohort studies. *Ageing Res Rev*. 2015;22:39-57. doi:10.1016/j.arr.2015.04.006

17. Xue M, Xu W, Ou Y-N, et al. Diabetes mellitus and risks of cognitive impairment and dementia: A systematic review and meta-analysis of 144 prospective studies. *Ageing Res Rev*. 2019;55:100944. doi:10.1016/j.arr.2019.100944

18. Gudala K, Bansal D, Schifano F, Bhansali A. Diabetes mellitus and risk of dementia: A meta-analysis of prospective observational studies. *J Diabetes Investig*. 2013;4(6):640-650. doi:10.1111/jdi.12087

19. Scosyrev E, Glimm E. Power analysis for multivariable Cox regression models. *Stat Med*. 2019;38(1):88-99. doi:10.1002/sim.7964

20. Royston P, Lambert PC. *Flexible Parametric Survival Analysis Using Stata: Beyond the Cox Model*. Stata Press; 2011.

21. Kivimäki M, Lawlor DA, Davey Smith G, et al. Socioeconomic position, co-occurrence of behavior-related risk factors, and coronary heart disease: the Finnish Public Sector study. *Am J Public Health*. 2007;97(5):874-879. doi:10.2105/AJPH.2005.078691

22. Aalto A-M, Elovainio M, Kivimäki M, Uutela A, Pirkola S. The Beck Depression Inventory and General Health Questionnaire as measures of depression in the general population: a validation study using the Composite International Diagnostic Interview as the gold standard. *Psychiatry Res*. 2012;197(1-2):163-171. doi:10.1016/j.psychres.2011.09.008

23. Fransson EI, Heikkilä K, Nyberg ST, et al. Job strain as a risk factor for leisure-time physical inactivity: an individual-participant meta-analysis of up to 170,000 men and women: the IPD-Work Consortium. *Am J Epidemiol*. 2012;176(12):1078-1089. doi:10.1093/aje/kws336

24. Lévesque LE, Hanley JA, Kezouh A, Suissa S. Problem of immortal time bias in cohort studies: example using statins for preventing progression of diabetes. *BMJ*. 2010;340:b5087. doi:10.1136/bmj.b5087

25. Korkeila K, Suominen S, Ahvenainen J, et al. Non-response and related factors in a nation-wide health survey. *Eur J Epidemiol*. 2001;17(11):991-999.

26. Väänänen A, Murray M, Koskinen A, Vahtera J, Kouvonen A, Kivimäki M. Engagement in cultural activities and cause-specific mortality: prospective cohort study. *Prev Med*. 2009;49(2-3):142-147. doi:10.1016/j.ypmed.2009.06.026

27. Kuper H, Marmot M. Job strain, job demands, decision latitude, and risk of coronary heart disease within the Whitehall II study. *J Epidemiol Community Health*. 2003;57(2):147-153.

28. Rusmaully J, Dugravot A, Moatti J-P, et al. Contribution of cognitive performance and cognitive decline to associations between socioeconomic factors and dementia: A cohort study. *PLoS Med*. 2017;14(6):e1002334. doi:10.1371/journal.pmed.1002334

29. Virtanen M, Vahtera J, Singh-Manoux A, Elovainio M, Ferrie JE, Kivimäki M. Unfavorable and favorable changes in modifiable risk factors and incidence of coronary heart disease: The Whitehall II cohort study. *Int J Cardiol*. 2018;269:7-12. doi:10.1016/j.ijcard.2018.07.005

30. Williams B, Mancia G, Spiering W, et al. 2018 ESC/ESH Guidelines for the management of arterial hypertension. *Eur Heart J*. 2018;39(33):3021-3104. doi:10.1093/eurheartj/ehy339

31. Zhao JH, Brunner EJ, Kumari M, et al. APOE polymorphism, socioeconomic status and cognitive function in mid-life--the Whitehall II longitudinal study. *Soc Psychiatry Psychiatr Epidemiol*. 2005;40(7):557-563. doi:10.1007/s00127-005-0925-y

32. Lind L, Sundström J, Ärnlöv J, Lampa E. Impact of Aging on the Strength of Cardiovascular Risk Factors: A Longitudinal Study Over 40 Years. *J Am Heart Assoc*. 2018;7(1). doi:10.1161/JAHA.117.007061
